# Independent and joint effects of lifestyle, human leukocyte antigen polymorphisms on cancer risk: findings from the UK Biobank

**DOI:** 10.3389/fimmu.2026.1899135

**Published:** 2026-09-11

**Authors:** Jiali Zheng, Shuo Jiang, Hongyi Zhu, Feng Zhang, Longgang Zhao, Jingqi Zhou, Chengdong Zhang, Xiaoguang Li, Hui Wang

**Affiliations:** 1 School of Public Health, Shanghai Jiao Tong University School of Medicine, Shanghai, China; 2 University of Texas MD Anderson Cancer Center, Houston, TX, United States; 3 Yale School of Nursing, Yale University, New Haven, CT, United States; 4 State Key Laboratory of Systems Medicine for Cancer, Center for Single-Cell Omics, School of Public Health, Shanghai Jiao Tong University School of Medicine, Shanghai, China; 5 Hainan International Medical Center, Shanghai Jiao Tong University School of Medicine, Qionghai, Hainan, China

**Keywords:** cancer immunology, cancer risk, cohort study, HLA polymorphisms, interaction, lifestyle

## Abstract

**Introduction:**

Immune genetic variation and lifestyle factors jointly influence cancer susceptibility, but their interactions in cancer development remain unclear. We evaluated the independent and joint effects of lifestyle profiles and classical HLA alleles on overall and site-specific cancer risks using sex-stratified analyses.

**Methods:**

Among 159, 744 men and 175, 037 women of European ancestry in the UK Biobank, 50 common HLA alleles from 11 classical HLA genes were imputed. A healthy lifestyle index incorporating diet, smoking, alcohol intake, physical activity, and adiposity classified participants into favorable, intermediate, or poor lifestyle categories. Independent effects of HLA polymorphisms and lifestyle factors on cancer risk were assessed using logistic regression and Cox proportional hazards models, respectively. Additive and multiplicative interactions were evaluated for cancers associated with both lifestyle and HLA alleles using Cox model.

**Results:**

During a median follow-up of 13 years, 35, 132 incident cancers occurred. Poor lifestyle increased overall cancer risk by 38% in men and 44% in women. Several HLA alleles were associated with lung, prostate, and non-Hodgkin lymphoma risks in men and kidney and endometrial cancer risks in women. Significant additive interactions were observed between poor lifestyle and a cluster of AH8.1-related HLA alleles (HLA-B*08:01, DQA1*05:01, DQB1*02:01, and DRB1*03:01) for lung cancer in men (relative excess risk due to interaction (RERI)=2.63–3.24), and between poor lifestyle and HLA-A*24:02 for kidney cancer in women (RERI = 3.24, 95%CI=1.20–5.28).

**Discussion:**

In sex-stratified analyses, unhealthy lifestyles markedly amplified lung cancer risk in men and kidney cancer risk in women among carriers of specific HLA risk alleles. These findings warrant independent replication, particularly in non-European populations and in datasets with directly typed or sequenced HLA alleles.

## 1 Introduction

Cancer remains a leading cause of mortality worldwide, with both lifestyle factors and immune function playing pivotal roles in its pathogenesis (1). Immune competence—particularly mediated by the highly polymorphic human leukocyte antigen (HLA) system—influences cancer susceptibility through the recognition of tumor antigens and immune surveillance (2). HLA genes encode for critical proteins responsible for antigen presentation to the T cells: class I molecules (HLA-A, -B, and -C) present intracellular peptides to CD8^+^ T cells, whereas class II (HLA-DR, -DQ, and -DP) present extracellular peptides to CD4^+^ T cells, thereby orchestrating adaptive immune responses (3). The extreme polymorphism of HLA genes affects peptide-binding repertoire, binding affinity, and T-cell receptor engagement, which in turn may predispose individuals with certain HLA alleles to specific cancers (3).

Lifestyle factors—such as tobacco use, alcohol consumption, diet, physical activity and obesity—can profoundly influence cancer risk, partly through immune modulation (4). For instance, tobacco smoke or dietary carcinogens can induce DNA mutations and generate neoantigens; the immune system’s ability to recognize these neoantigens is largely determined by HLA polymorphisms (5). A poor lifestyle may also exacerbate the detrimental effects of certain immune gene variations by promoting genetic mutations or epigenetic changes while also heightening inflammation and disrupting normal cell process. If an individual’s HLA variants exhibit low affinity for such lifestyle-induced neoantigen, immune surveillance may fail, facilitating malignant outgrowth (6). Conversely, a healthy lifestyle may reduce neoantigen burden and attenuate inflammation, potentially mitigating cancer risk even in the presence of risk-associated HLA genotypes.

However, the interaction between integrated lifestyle patterns and HLA genetic variability in relation to cancer susceptibility remains poorly characterized. Moreover, lifestyle factors tend to cluster, underscoring the importance of analyzing composite lifestyle pattern rather than isolated behaviors. Although previous studies have examined the collective influence of lifestyle on overall cancer risk (7–11) or specific malignancies (12), none have systematically evaluated how these patterns interact with HLA polymorphisms among men and women separately, given the apparent differences in lifestyle characteristics and cancer susceptibility between men and women.

To address this gap, we conducted a large-scale prospective study using UK Biobank data to systematically evaluate the independent and joint effects of composite lifestyle profiles and classical HLA alleles on overall and site-specific cancer risks, with stratification by sex. We further examined whether unhealthy lifestyle behaviors synergized with specific HLA variants to alter cancer risk—thereby identifying subgroups that would benefit most from risk-stratified cancer prevention strategies.

## 2 Materials and methods

### 2.1 Study design and participants

The UK Biobank is a prospective cohort study that included ~500, 000 individuals aged 40–69 recruited from across the nation during 2006-2010, with detailed protocol publicly available (13). Each eligible participant at recruitment provided electronic signed consent and extensive health-related information through extensive baseline questionnaires, interviews, and biological and physical measurements. Blood samples were collected at baseline on all individuals for genome-wide genotyping.

Among participants of European ancestry at baseline (n=409, 551), we excluded participants with baseline malignancies (n=45, 811), had withdrawn their consent to participate (n=12), with errors in incidence cancer dates (n=1), and those with sex chromosome aneuploidy, excessive heterozygosity or missingness rate >5%, genetic relatedness up to the third degree and nonconformity between reported and genetically inferred sex (n=28, 946). Finally, a total of 334, 781 participants were included in the analysis, comprising 159, 744 men and 175, 037 women. The detailed selection procedure was described in **Supplementary Figure 1**. The UK Biobank study was approved by the North West Multicenter Research Ethical Committee. The present study was conducted under application number 83432 of the UK Biobank resource.

### 2.2 HLA genotyping and imputation

Detailed information on genotype calling, quality control and imputation have been described elsewhere (14). Briefly, participants were genotyped using the Applied Biosystems™ UK BiLEVE Axiom™ Array and Applied Biosystems™ UK Biobank Axiom™ Array, which were designed specifically for the UK Biobank genotyping project. They totally cover ~825, 000 markers and share 95% of their marker content to effectively capture genome-wide genetic variation (14, 15). Genotypes were phased and imputed using computationally efficient methods combined with the Haplotype Reference Consortium and UK10K haplotype resource (14). UK Biobank imputed allelic variation at 11 classical HLA genes at two-field resolution for HLA-A, -B, -C, -DRB5, -DRB4, -DRB3, -DRB1, -DQB1, -DQA1, -DPA1, -DPB1 using the specialized HLA*IMP:02 algorithm with a multi-population reference panel (16, 17). A total of 362 HLA alleles were imputed. There are two imputations for each locus, giving the HLA genotype. We imposed posterior thresholding at 0.7 for each HLA allele, following the recommendation by UK Biobank (18), which gave accuracy of imputation above 96.1% and call rate above 95.1% for all 11 loci from five-fold cross-validation (14). Among subset of 409, 724 white British individuals in the UK Biobank, signals of known associations between HLA alleles and common immune-mediated diseases had been replicated, indicating the imputation of HLA alleles is sufficiently accurate for genetic association testing (14).

Allele frequencies at HLA Class I and Class II loci were calculated as the percentage of copies of each allele in the sample. To increase statistical power, we analyzed HLA alleles with allele frequency >5% in this study. **Supplementary Table 1** displayed the frequencies of the 50 HLA alleles included in our study for men and women separately.

### 2.3 Lifestyle categories assessed with Healthy Lifestyle Index

We constructed a Healthy Lifestyle Index (HLI) from five established cancer risk factors assessed at baseline: smoking, alcohol drinking, physical activity, weight, and diet (19). These factors were all self-reported using a touchscreen questionnaire, except for weight as assessed by staff-measured body mass index (BMI) and waist circumference. Based on the 2018 World Cancer Research Fund/American Institute for Cancer Research (WCRF/AICR) cancer prevention recommendations and relevant literature evidence, participants scored 1 point for each of 5 healthy behaviors―non-current smokers (defined as never smokers or former smokers who had quit >30 years), never alcohol drinking, regular physical activity, healthy weight and healthy diet; and 0 point otherwise, with details of definitions and grading criteria described (**Supplementary Table 2**) (11, 19–21). The HLI scores ranged from 0 to 5, with a higher value indicating a healthier lifestyle, and were subsequently categorized as favorable (HLI score 4 to 5), intermediate (HLI score 2 to 3), and poor (HLI score 0 to 1) lifestyle. As an alternative version, we created a six-factor HLI with components of smoking, alcohol drinking, physical activity, weight, vegetables and fruits, red meat and processed meat intake, and we divided individuals into 3 categories for each component (ideal, intermediate, poor) to provide refined classifications of cancer-related lifestyles and better compliance with the 2018 WCRF/AICR recommendations (19). Individuals were subsequently classified to 3 lifestyle groups: favorable (≥4 ideal lifestyle factors), intermediate (between the favorable and poor lifestyles), and poor (≥4 poor lifestyle factors) lifestyle, with detailed grading criteria listed in **Supplementary Table 2**.

### 2.4 Ascertainment of incident cancer outcomes

Incident cancer cases were ascertained through record electronic linkage with national cancer registries. The first-occurred malignant cancer during the follow-up was considered as the incident cancer outcome for an individual, and we censored benign tumors and carcinoma *in situ* at the date of diagnosis because these events often trigger clinical surveillance or treatment that may modify subsequent cancer risk, whereas the present study aimed to evaluate etiological associations with first primary invasive cancers. Based on power considerations, a minimum of 280 cases was required to obtain stable estimates in the Logistic and Cox regression models, thus, only cancer sites meeting this threshold were included in the sex-stratified analyses (22, 23). Consequently, the outcomes in this study comprised a total of 16 individual cancers (14 for men and 10 for women). Additionally, two composite outcomes were analyzed: 1) other cancers (i.e., other malignant cancers beyond the studied cancers), and 2) overall cancer (i.e., diagnosis of any of 342 types of malignant cancers in the UK Biobank). The included cancer types and their incidence rates were listed (**Supplementary Table 3**).

### 2.5 Assessment of covariates

Age at recruitment, sex, and Townsend deprivation index were collected at recruitment. Townsend deprivation index is a composite measure of deprivation based on unemployment, non-car ownership, non-home ownership, and household overcrowding, with a lower score indicating a higher level of socioeconomic status (24). Education, ethnicity, and family history of cancer were self-reported on the baseline touchscreen questionnaires. Family history of cancer was assessed by asking participants whether their mothers or fathers had been diagnosed with breast cancer, bowel cancer, lung cancer, or prostate cancer. Standing height was measured by trained staff via a Seca 202 device. Prevalent chronic diseases—including type 2 diabetes, dyslipidemia, hypertension, cardiovascular disease, respiratory disease, digestive disease, and chronic kidney disease—were defined through self-reports, medication use and hospital inpatient records (**Supplementary Table 4**). Detailed covariates information was available in **Supplementary Methods**.

### 2.6 Statistical analyses

All analyses were conducted separately for men and women with the study design flowchart illustrated in **Supplementary Figure 2**. Participants were followed up from baseline to incident cancer diagnosis, loss to follow-up, death, or end of study (30 Nov 2023), whichever came first. The baseline characteristics were presented with medians and interquartile ranges for continuous variables and number and frequencies for categorical variables according to three lifestyle categories. For each cancer outcome, logistic regression was used to estimate associations between HLA allele dosage and site-specific cancer risk, adjusting for baseline age, assessment center, and population structure (top five genetic principal components) (25). Statistical significance was defined as *P* < 0.001 (=0.05/50) after Bonferroni correction for the 50 common HLA alleles tested within each cancer outcome (26). Independent associations between lifestyle and cancer incidence were evaluated using multivariable Cox proportional hazards models with follow-up time as the underlying time scale, adjusting for baseline age, height, Townsend deprivation index, education, and family history of cancer. Hazard ratios (HRs) and 95% confidence intervals (CIs) were estimated for intermediate and poor lifestyle categories relative to the favorable group (11, 27). Missing covariates were treated as a separate category. Linear trends were tested using median lifestyle index values, and continuous associations were assessed after confirming linearity via natural cubic splines with 5 knots at 5th, 25th, 50th, 75th, and 95th percentiles (28). For analyses of associations between HLI or individual lifestyle factors and cancer outcomes, statistical significance was defined using a Bonferroni-corrected threshold of *P* < 0.05/N, where N represented the number of cancer outcomes evaluated separately in men and women. Individual lifestyle factors were analyzed in the mutually adjusted models. Proportional hazards assumptions were verified using Schoenfeld residuals. For cancers significantly associated with the lifestyle index, cumulative incidence curves up to age 75 stratified by lifestyle groups were estimated.

HLA–lifestyle interactions were evaluated in a two-stage analytical framework. First, a comprehensive screening of all possible HLA–lifestyle interactions was conducted for each cancer outcome using multivariable-adjusted Cox models, with individuals having a favorable lifestyle and cancer-protective HLA status as the reference. Both multiplicative and additive interactions were evaluated. Because 50 common HLA alleles were tested in interaction with HLI for each cancer outcome, statistical significance in this comprehensive interaction screening was defined as *P* < 0.001 (=0.05/50) after Bonferroni correction. Multiplicative interaction was tested by incorporating an interaction term (lifestyle categories×binary HLA carrier status), and additive interaction was assessed using the relative excess risk due to interaction (RERI) with 95% CIs estimated by delta method (29). Second, among the interactions identified in the screening stage, we conducted targeted follow-up analyses restricted to cancer outcomes for which both the corresponding HLA–cancer and lifestyle–cancer associations reached their predefined significance thresholds in the primary analyses and for which a positive additive or multiplicative interaction was observed in the screening stage. As no significant multiplicative interactions were detected, subsequent follow-up analyses focused on the identified significant additive interactions. Given the substantially reduced number of analyses after this prespecified selection step, statistical significance for these targeted follow-up additive interaction analyses was evaluated using a two-sided *P* < 0.05. For significant additive interactions, we evaluated joint effects by estimating adjusted HRs with 95% CIs for each exposure combination, as well as extrapolating the 5-year absolute risk differences via bootstrapping (1, 000 resamples) (30). To further aid interpretation of the observed additive interactions, we performed additive interaction analyses between each lifestyle factor and the specific HLA allele that exhibited the notable interaction for the corresponding cancer outcome, using a two-sided *P* < 0.05. Of note, given that the investigated cancer outcomes were relatively rare and no meaningful violations of the proportional hazards assumption were found for the joint exposure groups involved in the identified additive interactions, Cox-model-derived RERI estimates were considered reasonable approximations for assessing additive interactions in the analyses.

Several sensitivity analyses were performed. Firstly, we treated HLA allele as a binary variable (carrier/non-carrier) in the analyses regarding with their independent effects on cancer risk. Secondly, we replaced HLI score with six-factor HLI score throughout the analyses. Third, we supplemented incident cancer cases from sources of hospital inpatient records and death registries. Forth, we reconstructed COX models by additionally adjusting for prevalent diseases at baseline; Fifth, for each cancer outcome, competing risk models were used in the site-specific cancer associations to account for the competing causes of other incident cancers. Sixth, we excluded incident cancer cases occurred in the first year of follow-up to minimize reverse causality. Seventh, the primary HLA–cancer association analyses were repeated using Cox proportional hazards models, consistent with the time-to-event modeling framework used for the lifestyle and HLA–lifestyle interaction analyses. Eighth, to assess the potential influence of the small number of cases in several joint-exposure strata on the observed significant additive interactions, we conducted additional stability analyses including stratified non-parametric bootstrap analyses with 1, 000 resamples, model influence diagnostics using standardized DFBETAs, and sparse-cell case-deletion analyses. Ninth, the significant HLA–lifestyle interaction analyses for lung cancer among men were repeated with additional adjustment for detailed smoking-related variables, including pack-years, age at smoking cessation, and smoking cessation duration, to assess the potential influence of smoking on these observed interactions. Tenth, for female-specific cancers, including breast, ovarian, and endometrial cancers, the corresponding lifestyle–cancer association and interaction models were additionally adjusted for reproductive and hormone-related variables, including age at menarche, menopausal status, number of live births, age at first live birth, ever use of oral contraceptive pills, and ever use of hormone replacement therapy. Lastly, a complete-case analysis was performed to assess the potential impact of missing covariate data. All statistical analyses were performed with SAS (version 9.4, Cary, NC) and R software (version 3.5.1).

## 3 Results

### 3.1 Characteristics of the study population

Among 159, 744 men (mean [SD] age, 56.8 [8.0] years) and 175, 037 women (mean [SD] age, 56.5 [7.9] years), the proportion of favorable, intermediate and poor lifestyle conformers was 7.5%, 64.3%, 28.2% for men and 12.0%, 64.9% and 23.1% for women respectively, with corresponding cancer incidence rates of 8.9, 9.7, 11.9 per 1000 person-years for men and 6.6, 7.0, 9.0 per 1000 person-years for women. During a median follow-up period of 13.05 years (interquartile range: 12.07–13.85), there were a total of 35, 132 incident cancer cases, comprising 19, 290 cases in men and 15, 842 cases in women. Top three incident cancers for men were prostate cancer, colorectal cancer, and lung cancer, and top three cancers for women were breast cancer, colorectal cancer, and lung cancer (**Supplementary Table 3**). For both men and women, individuals with a poor lifestyle were more likely to be socioeconomically deprived, less educated, current or former smokers, heavy drinkers, and to have higher BMI and waist circumference, engage in less physical activity, consume a less healthy diet, and exhibit a higher prevalence of chronic diseases at baseline compared with those adhering to a healthy lifestyle (**Table 1**).

**Table 1:** Baseline characteristics of the study population in the UK Biobank study by sex and lifestyle categories.

Characteristics		Men (N = 159, 744)			Women (N = 175, 037)	
	Lifestyle categories ^a^			Lifestyle categories ^a^		
	Favorable (4-5)	Intermediate (2-3)	Poor (0-1)	Favorable (4-5)	Intermediate (2-3)	Poor (0-1)
N (%)	11925 (7.5)	102727 (64.3)	45092 (28.2)	20953 (12.0)	113720 (64.9)	40364 (23.1)
Crude incidence rate of cancer^b^	8.9	9.7	11.9	6.6	7.0	9.0
Age at recruitment, median (IQR), y	59.0 (51.0, 64.0)	58.0 (50.0, 63.0)	59.0 (52.0, 64.0)	59.0 (52.0, 64.0)	57.0 (50.0, 63.0)	57.0 (50.0, 63.0)
Townsend deprivation index, median (IQR)	-2.7 (-3.9, -0.7)	-2.5 (-3.8, -0.2)	-1.8 (-3.5, 1.2)	-2.7 (-4.0, -0.8)	-2.5 (-3.8, -0.3)	-1.8 (-3.4, 1.1)
Educational level, n (%) ^c^						
College or university degree/vocational qualification	8610 (72.2)	67766 (66.0)	24677 (54.7)	13134 (62.7)	65777 (57.8)	19413 (48.1)
National examination at age 17-18	544 (4.6)	5046 (4.9)	2229 (4.9)	1244 (5.9)	6598 (5.8)	2137 (5.3)
National examination at age 16	1298 (10.9)	13888 (13.5)	7019 (15.6)	3801 (18.1)	22871 (20.1)	8676 (21.5)
Unknown	1473 (12.4)	16027 (15.6)	11167 (24.8)	2774 (13.2)	18474 (16.2)	10138 (25.1)
Smoking status, n (%)						
Never smokers	10593 (88.8)	61142 (59.5)	6891 (15.3)	19592 (93.5)	75859 (66.7)	9588 (23.8)
Past smokers who had quit >30 y	1301 (10.9)	7420 (7.2)	949 (2.1)	1210 (5.8)	4419 (3.9)	572 (1.4)
Past smokers who had quit ≤30 y	13 (0.1)	15848 (15.4)	18708 (41.5)	64 (0.3)	14208 (12.5)	13332 (33.0)
Past smokers with unknown quit years	7 (0.1)	9694 (9.4)	7580 (16.8)	53 (0.3)	12340 (10.9)	8557 (21.2)
Current smokers	10 (0.1)	8417 (8.2)	10630 (23.6)	32 (0.2)	6658 (5.9)	7987 (19.8)
Missing status	1 (0.0)	206 (0.2)	334 (0.7)	2 (0.0)	236 (0.2)	328 (0.8)
Alcohol drinking status, n (%) ^d^						
Never drinkers	1077 (9.0)	1462 (1.4)	98 (0.2)	3127 (14.9)	4093 (3.6)	250 (0.6)
Moderate drinkers	7480 (62.7)	59940 (58.3)	22153 (49.1)	10295 (49.1)	55926 (49.2)	17523 (43.4)
Heavy drinkers	2071 (17.4)	29459 (28.7)	16185 (35.9)	4085 (19.5)	30185 (26.5)	11685 (28.9)
Missing status	1297 (10.9)	11866 (11.6)	6656 (14.8)	3446 (16.4)	23516 (20.7)	10906 (27.0)
Physical activity, median (IQR), MET-min/wk	3066.0 (1902.0, 5119.5)	2186.0 (1104.0, 4198.0)	742.5 (346.5, 1554.0)	2982.0 (1853.0, 4950.0)	1866.0 (930.0, 3546.0)	702.0 (344.0, 1386.0)
BMI, median (IQR), kg/m^2^	25.7 (23.8, 27.6)	26.7(24.7, 28.9)	30.5 (27.0, 33.2)	24.5 (22.4, 26.8)	25.6 (23.2, 28.4)	30.9 (26.1, 34.3)
Waist circumference, median (IQR), cm	91.0 (85.0, 96.0)	94.0 (88.0, 100.0)	105.0 (96.0, 112.0)	78.0 (72.0, 84.0)	81.0 (75.0, 89.0)	94.0 (84.0, 102.0)
Diet components						
Fruits, median (IQR), servings/day	4.0 (3.0, 6.0)	2.0 (1.0, 3.5)	2.0 (1.0, 3.0)	4.0 (3.0, 6.0)	3.0 (2.0, 4.0)	2.0 (1.0, 3.0)
Vegetables, median (IQR), tablespoons/day	5.0 (3.5, 7.0)	4.0 (3.0, 6.0)	4.0 (2.5, 5.0)	5.0 (4.0, 7.0)	4.0 (3.0, 6.0)	4.0 (3.0, 6.0)
Fish, median (IQR), times/wk	2.0 (2.0, 4.0)	2.0 (1.0, 3.5)	1.5 (1.0, 2.0)	2.0 (2.0, 4.0)	2.0 (1.5, 3.5)	1.5 (1.0, 2.0)
Processed meat, median (IQR), times/wk	0.5 (0.5, 1.0)	1.0 (0.5, 3.0)	3.0 (1.0, 3.0)	0.5 (0.5, 1.0)	1.0 (0.5, 1.0)	1.0 (0.5, 3.0)
Red meat, median (IQR), times/wk	1.5 (1.0, 1.5)	2.0 (1.5, 2.5)	2.0 (1.5, 3.0)	1.5 (1.0, 1.5)	1.5 (1.5, 2.5)	2.0 (1.5, 3.0)
Whole grains, median (IQR), servings/day	14.0 (7.0, 22.0)	7.0 (0.0, 18.0)	4.0 (0.0, 14.0)	11.0 (7.0, 17.0)	8.0 (3.0, 14.0)	6.0 (0.0, 12.0)
Height, median (IQR), cm	176.0 (172.0, 181.0)	176.0 (171.5, 180.5)	176.0 (171.0, 180.0)	163.0 (159.0, 167.0)	163.0 (159.0, 167.0)	162.0 (158.0, 166.0)
Family history of cancer, n (%)^e^	3667 (30.8)	31571 (30.7)	13801 (30.6)	6520 (31.1)	35507 (31.2)	12574 (31.2)
Prevalent diseases at baseline						
Type-2 diabetes, n (%)	372 (3.1)	4988 (4.9)	5133 (11.4)	382 (1.8)	3141 (2.8)	2511 (6.2)
Dyslipidemia, n (%)	2345 (19.7)	21730 (21.2)	14848 (32.9)	2348 (11.2)	13936 (12.3)	7967 (19.7)
Hypertension, n (%)	3020 (25.3)	30498 (29.7)	20139 (44.7)	4217 (20.1)	26195 (23.0)	13700 (33.9)
Cardiovascular disease, n (%)	899 (7.5)	8017 (7.8)	6478 (14.4)	599 (2.9)	4065 (3.6)	2574 (6.4)
Respiratory disease, n (%)	93 (0.8)	1609 (1.6)	1890 (4.2)	171 (0.8)	1526 (1.3)	1389 (3.4)
Digestive disease, n (%)	2950 (24.7)	28163 (27.4)	14655 (32.5)	4992 (23.8)	29971 (26.4)	13672 (33.9)
Chronic kidney disease, n (%)	167 (1.4)	1741 (1.7)	1175 (2.6)	154 (0.7)	956 (0.8)	529 (1.3)

^a^ The percentages for categorical variables in each lifestyle group by sex may not add up to 100% because of rounding and lifestyle groups were categorized to ideal, intermediate and poor lifestyles based on HLI scores.

^b^ Incidence rate of total cancer cases was represented as number of cases per 1000 person-years.

^c^ Educational levels were classified as ‘College or university degree/vocational qualification’ (i.e., the highest qualification level including college or university degree’, ‘NVQ or HND or HNC or equivalent’, and ‘other professional qualifications e.g.: nursing, teaching’); ‘National examination at age 17-18’(i.e., the intermediate qualification level including ‘A levels/AS levels or equivalent’); ‘National examination at age 16’(i.e., the lowest qualification level including ‘O levels/GCSEs or equivalent’, and ‘CSEs or equivalent’; and ‘Unknown’(including ‘none of the above’, ‘prefer not to answer’, and missing).

^d^ Alcohol drinking status was categorized into four groups, including ‘never drinkers’, ‘moderate drinkers’ (past drinkers or current drinkers with alcohol consumption ≤28 g/day for men and ≤14 g/day for women), ‘heavy drinkers’ (alcohol consumption >28 g/day for men and >14 g/day for women), and missing status.

^e^ Family history of cancer was measured based on whether the mother or father suffered from breast cancer, bowel cancer, lung cancer or prostate cancer.

BMI, body mass index; HLI, Healthy Lifestyle Index; IQR, interquartile range; MET-min, metabolic equivalent task minutes; PA, physical activity; wk, week; y, year.

### 3.2 Sex-stratified associations between HLA alleles and cancer risk

Among men, significant HLA-cancer associations were present on prostate cancer, lung cancer, and non-Hodgkin lymphoma. Four alleles were significantly associated with prostate cancer risk; only HLA*-*C*03:04 increased risk per allele (OR = 1.11, 95%CI=1.05-1.18) while C*04:01, DQB1*02:01 and DRB1*03:01 showed inverse associations. HLA-DRB1*01:01 was significantly associated with an increased risk of non-Hodgkin lymphoma (OR = 1.35, 95%CI=1.15-1.58). For lung cancer, five alleles (HLA-B*08:01, DQA1*05:01, DQB1*02:01, DRB1*03:01, DRB3*01:01) were positively associated with ORs from 1.19 to 1.30, while DRB1*01:01 decreased risk by 21% per allele (**Table 2**). Given that four of the lung cancer risk–increasing alleles identified in our analyses (HLA-B*08:01, HLA-DQA1*05:01, HLA-DQB1*02:01, and HLA-DRB1*03:01) are well-recognized components or markers of the ancestral haplotype 8.1 (AH8.1), classically defined as HLA-A*01:01~ C*07:01~ B*08:01~DRB1*03:01~DQA1*05:01~ DQB1*02:01 (31), we further examined the correlation structure among these alleles. These identified alleles showed moderate-to-strong pairwise linkage disequilibrium (LD) (r²=0.30–0.97; **Supplementary Figure 4**), suggesting that they represent a shared AH8.1-related genetic signal rather than independent associations. Therefore, we conducted an exploratory analysis of an AH8.1-related genetic signal using a simplified marker-based definition based on the presence of HLA-A*01, HLA-B*08, and HLA-DRB1*03 (32), and evaluated its association with lung cancer risk. This marker-based definition was chosen to reflect the AH8.1-related signal observed in the allele-based analyses, to capture information from both HLA class I and class II regions, and to provide a parsimonious representation of the highly correlated alleles within this LD block. Nevertheless, this marker-based definition should be regarded as a proxy for an AH8.1-related genetic signal rather than as the canonical AH8.1 ancestral haplotype itself. Men carrying this AH8.1-related marker status showed an increased risk of lung cancer compared with non-carriers, with a multivariable-adjusted OR of 1.31 (95%CI=1.13-1.50) (**Supplementary Table 5**).

**Table 2:** Significant associations by sex between HLA allele dosage and incident site-specific cancer risk.

Cancer types ^a^	HLA allele	AF (%) ^b^	Multivariable-adjusted OR (95%CI) ^c^	*P* value ^d^
Men				
Prostate cancer	C*03:04	8.03	1.11 (1.05-1.18)	8.51E-04
	C*04:01	8.24	0.90 (0.85-0.96)	7.51E-05
	DQB1*02:01	14.89	0.92 (0.88-0.96)	3.90E-04
	DRB1*03:01	14.81	0.91 (0.87-0.95)	7.51E-05
Lung cancer	B*08:01	14.47	1.19 (1.08-1.32)	5.99E-04
	DQA1*05:01	23.14	1.22 (1.12-1.33)	5.03E-06
	DQB1*02:01	14.89	1.30 (1.18-1.44)	7.25E-08
	DRB1*01:01	9.31	0.79 (0.68-0.91)	9.39E-04
	DRB1*03:01	14.81	1.30 (1.18-1.43)	1.22E-07
	DRB3*01:01	16.71	1.23 (1.12-1.35)	1.79E-05
non-Hodgkin lymphoma	DRB1*01:01	9.31	1.35 (1.15-1.58)	2.35E-04
Women				
Endometrial cancer	B*07:02	14.78	1.23 (1.09-1.38)	6.28E-04
Kidney cancer	A*24:02	7.38	1.60 (1.25-2.03)	1.59E-04
	DPB1*04:01	43.80	1.30 (1.11-1.51)	9.90E-04

^a^ Cancer types that were significantly associated with HLA allele dosage in the logistic regression model adjusted for age at recruitment, assessment center, and population structure (top 5 principal components of the genotypes).

^b^ The allele frequency was calculated in men and women separately by simple counting methods where the sum of all allele frequencies at each locus was 1.

^c^ The multivariable-adjusted ORs and 95%CIs were computed by using the dosage of each HLA allele as a continuous variable in the logistic model.

^d^ For each cancer outcome, P <0.001 (0.05/50) was considered statistical significance in this analysis after Bonferroni correction. All significant associations were included in this table.

AF, allele frequency; CI, confidence interval; HLA, human leukocyte antigen; OR, odds ratio.

Among women, HLA-A*24:02 and DPB1*04:01 were positively related to kidney cancer with ORs of 1.60 and 1.30, and HLA-B*07:02 was associated with higher endometrial cancer risk (OR = 1.23, 95%CI=1.09-1.38) (**Table 2**). Sex-stratified associations for all HLA alleles and cancer outcomes are presented in **Supplementary Tables 6**–**15**.

### 3.3 Sex-stratified associations between lifestyle and incident cancer risk

**Figure 1:**
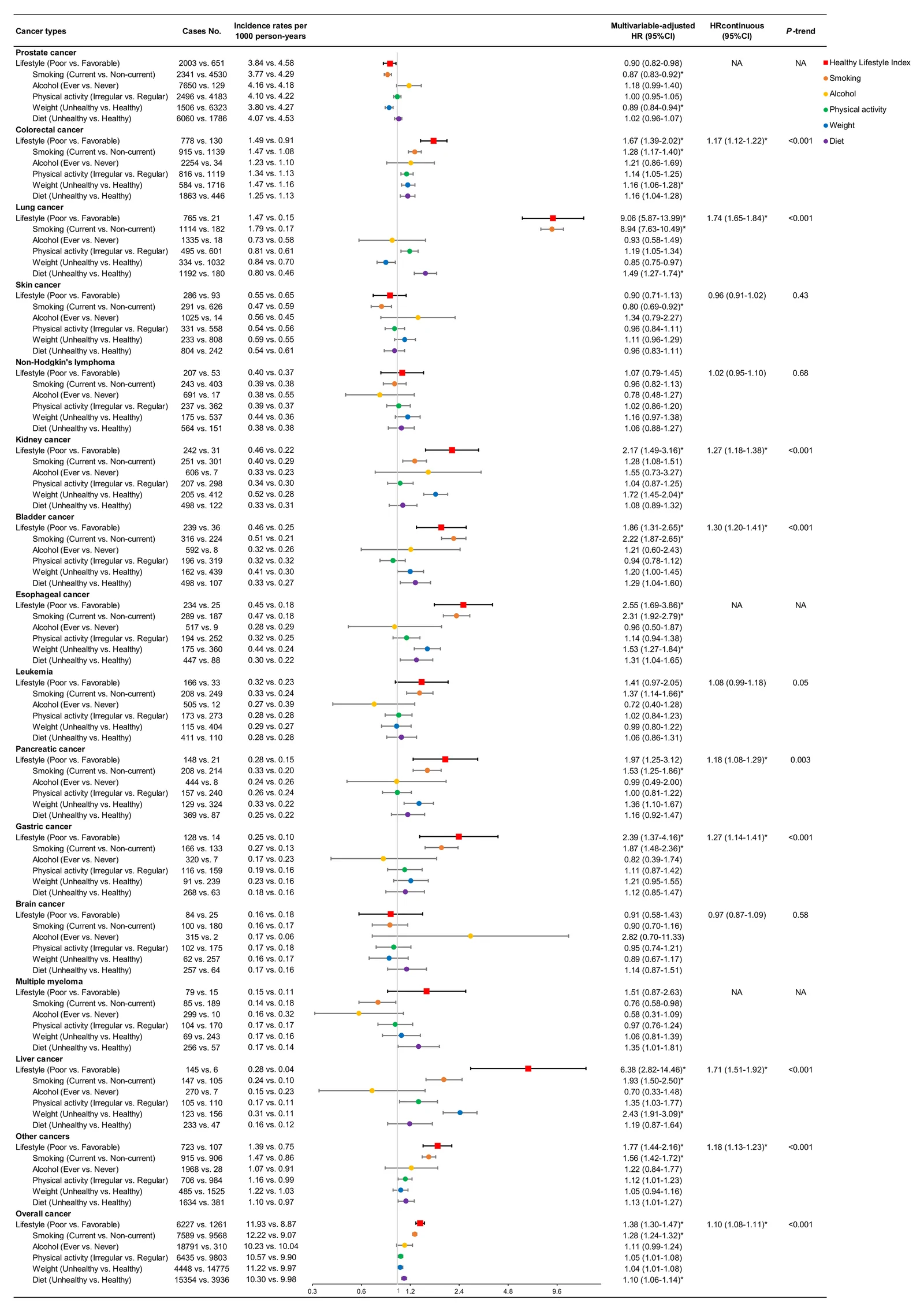
Multivariable-adjusted associations of lifestyle categories assessed with Healthy Lifestyle Index and five individual lifestyle factors with site-specific and overall cancer risks in the UK Biobank study among men. The other cancers included all malignant cancers except for the top 14 listed in this figure, and overall cancer was referred to having any one of 342 types of malignant cancers in the UK Biobank. The multivariable-adjusted HRs and 95%CIs were calculated by Cox regression model with adjustment for baseline age, height, education, Townsend deprivation index, and family history of cancer, treating the favorable lifestyle factor as the respective referent. The HR_continuous_ and 95%CIs were calculated for each unit decrease of the HLI score. *P* < 0.003125 (=0.05/16) was applied to determine statistical significance in all the analyses after Bonferroni correction. “NA” denoted a significant non-linear relationship between HLI score and cancer risk. The horizontal axis is displayed on a logarithmic scale with a base of two. CI, confidence interval; HLI, Healthy Lifestyle Index; HR, hazard ratio; WC, waist circumference. *Statistical significance after Bonferroni correction.

**Figure 2:**
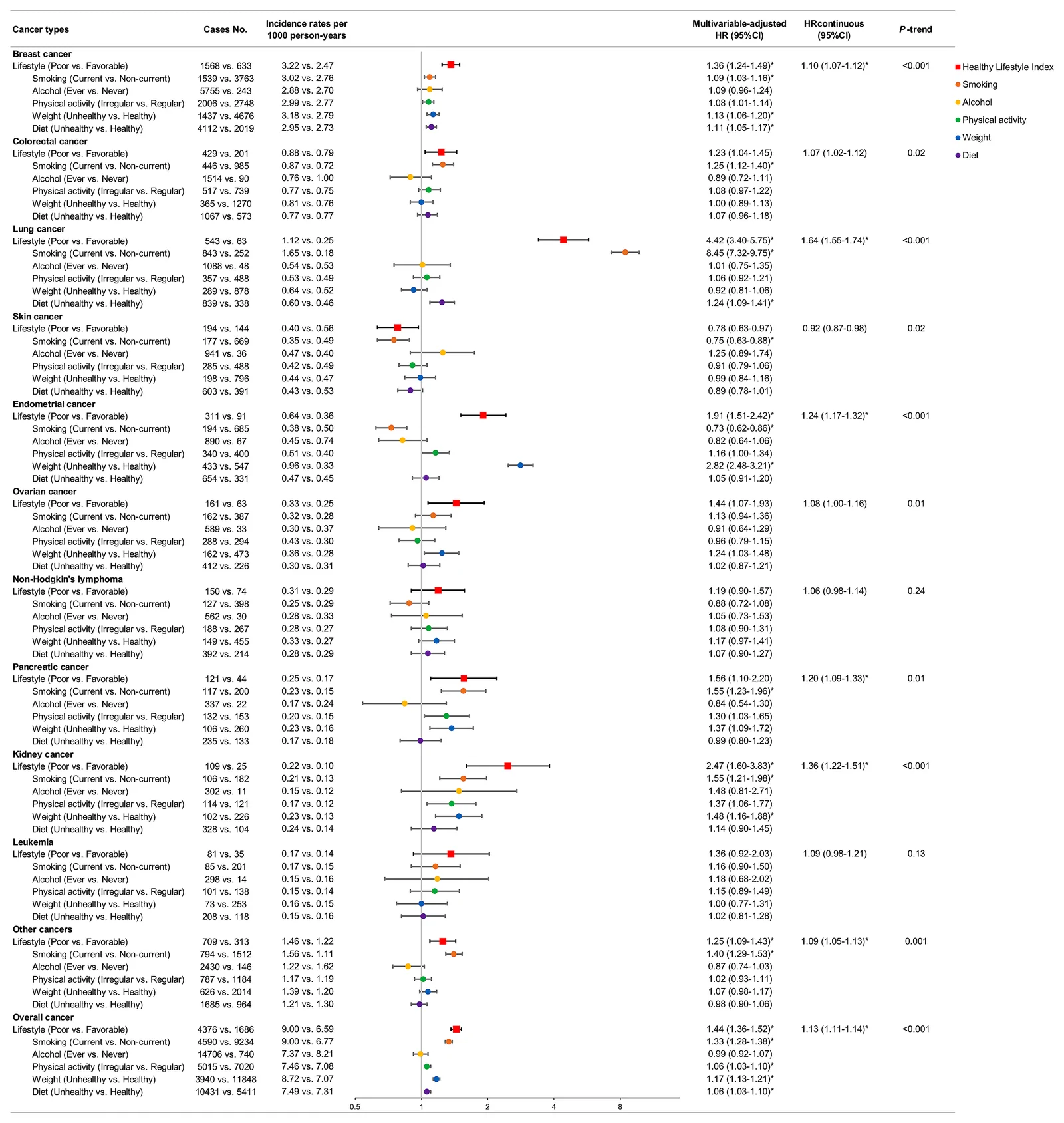
Multivariable-adjusted associations of lifestyle categories assessed with Healthy Lifestyle Index and five individual lifestyle factors with site-specific and overall cancer risks in the UK Biobank study among women. The other cancers included all malignant cancers except for the top 14 listed in the figure, and overall cancer was referred to having any one of 342 types of malignant cancers in the UK Biobank. The multivariable-adjusted HRs and 95%CIs were calculated by Cox regression model with adjustment for baseline age, height, education, Townsend deprivation index and family history of cancer, treating favorable lifestyle factor as the respective referent. The HR_continuous_ and 95%CIs were calculated by multivariable-adjusted Cox model for each unit decrease of the HLI score. *P* < 0.004167 (=0.05/12) was applied to determine statistical significance in all the analyses after Bonferroni correction. The horizontal axis is displayed on a logarithmic scale with a base of two. CI, confidence interval; HLI, Healthy Lifestyle Index; HR, hazard ratio; WC, waist circumference. *Statistical significance after Bonferroni correction.

As assessed with HLI, poor lifestyle was associated with a 38% higher total cancer risk in men (HR = 1.38, 95%CI=1.30-1.47) and a 44% higher risk in women (HR = 1.44, 95%CI=1.36-1.52) compared with a favorable lifestyle. For site-specific cancers, men with a poor lifestyle had significantly increased risks of colorectal, lung, kidney, bladder, esophageal, pancreatic, gastric, liver, and other cancers, with HRs ranging from 1.67 (colorectal) to 9.06 (lung), correlating to a 17%-74% higher risk per additional unhealthy factor (**Figure 1**). For women, poor lifestyle significantly increased the risk of breast, lung, endometrial, kidney, and other cancers, with HRs ranging from 1.25 to 4.42, corresponding to a 9%–64% increase per unhealthy factor (**Figure 2**). A 10% reduced prostate cancer risk (HR = 0.90, 95%CI=0.82-0.98) and 22% reduced skin cancer risk (HR = 0.78, 95%CI=0.63-0.97) was found to be related to poor lifestyle among men and women respectively, although these associations did not meet the Bonferroni-corrected significance threshold. Smoking had the greatest impact on most cancers for both sexes, except breast and endometrial cancer in women, for which unhealthy weight predominated. When looking into the cumulative risk of overall cancer by age 75, the estimated risks among individuals with favorable, intermediate and poor lifestyles were, on average, 0.174, 0.198 and 0.229 for men, and 0.123, 0.141 and 0.181 for women (**Supplementary Figure 3**).

### 3.4 Interactions between lifestyle and HLA alleles on incident cancer risk

**Figure 3:**
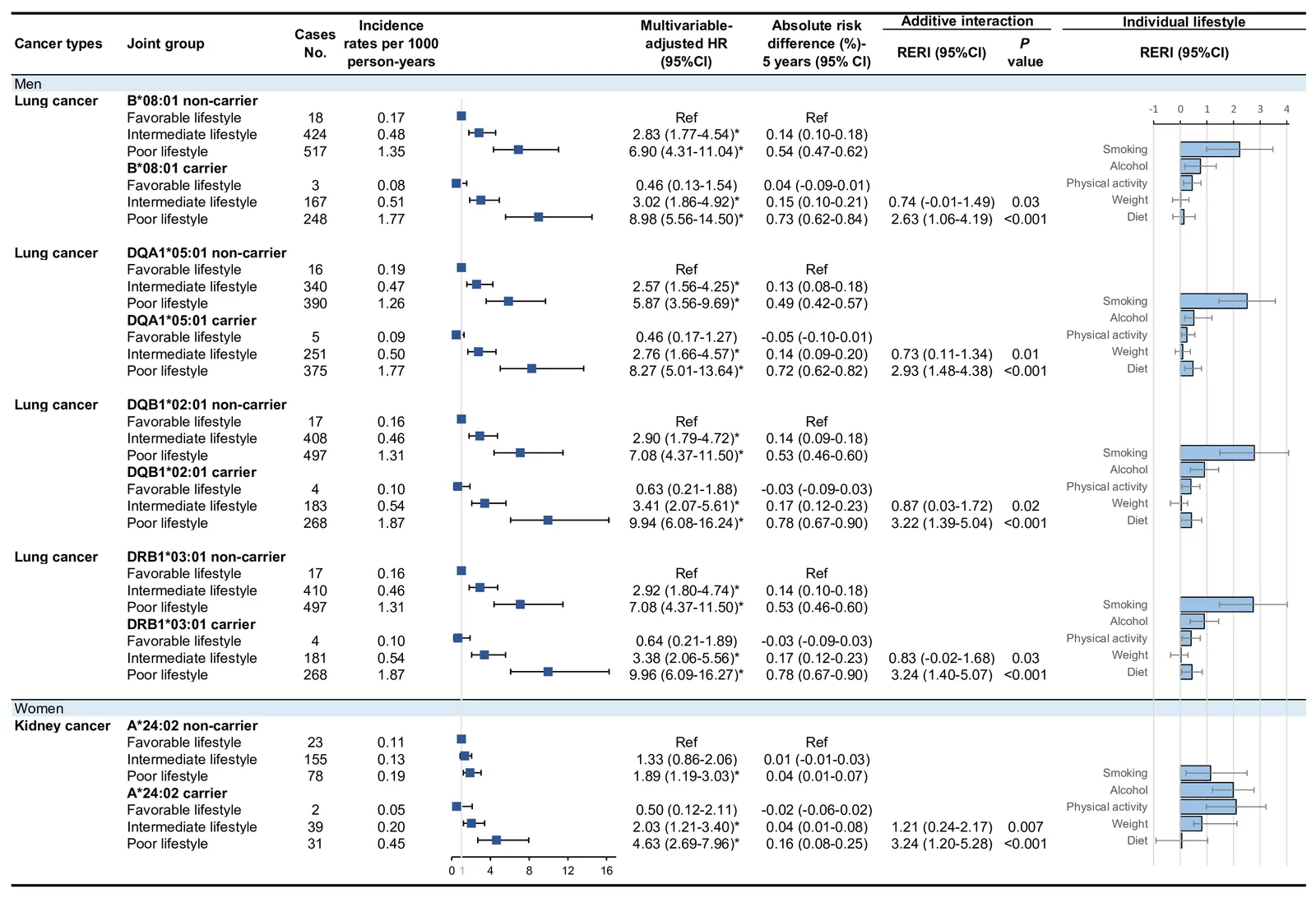
Significant interactions and joint effects between cancer-related HLA alleles and lifestyle categories on specific cancer risk. The selection of the cancer types and HLA alleles in this figure was based on the following conditions: both independent HLA and lifestyle effects reached statistical significance and *P* < 0.001 (=0.05/50) for the interaction (either additive or multiplicative). For each selected HLA in the figure, the allele carriers increased the corresponding cancer risk, the HLA allele non-carriers with the favorable lifestyle were considered as the referent in the joint effect analyses. HRs and 95% CIs were measured in the multivariable-adjusted Cox model, with baseline age, height, assessment center, Townsend deprivation index at recruitment, family history of cancer and population structure (top 5 principal components) adjusted. The absolute cancer incidence difference in 5-year event rates was estimated with 95% CIs from the bootstrapping method (N = 1, 000). CI, confidence interval; HLA, human leukocyte antigen; HR, hazard ratio; RERI, relative excess risk due to interaction. *Statistical significance in the joint effect analyses (*P* < 0.05).

We identified five HLA alleles that exhibited significant additive interactions with unhealthy lifestyles, leading to an increased risk of specific cancers (**Supplementary Tables 16**–**19**). As shown in **Figure 3**, in men, poor lifestyle demonstrated significant positive additive interactions with a cluster of AH8.1-related alleles to promote lung cancer risk, including HLA-B*08:01 (RERI = 2.63, 95%CI=1.06-4.19), DQA1*05:01 (RERI = 2.93, 95%CI=1.48-4.38), DQB1*02:01 (RERI = 3.22, 95%CI=1.39-5.04) and DRB1*03:01 (RERI = 3.24, 95%CI=1.40-5.07). Compared to men with a favorable lifestyle who did not carry these alleles, those carrying any of these AH8.1-related alleles combined with a poor lifestyle exhibited a significantly higher risk of lung cancer, with adjusted HRs ranging from 8.27 to 9.96 and an increased 5-year cancer incidence risk rising by 72% to 78%. Individuals with AH8.1-related marker status and an intermediate (HR = 4.16, 95%CI=2.22-7.83) or poor lifestyle (HR = 11.52, 95%CI=6.15-21.60) exhibited a markedly elevated risk of lung cancer compared with individuals with a favorable lifestyle and no AH8.1-related marker status. Additive interaction analyses further indicated significant interactions between the marker-defined AH8.1-related genetic signal and poor lifestyle (RERI = 2.34, 95%CI=0.48-4.19, *P* = 0.007) (**Supplementary Figure 5**).

In women, the detrimental impact of carrying HLA-A*24:02 on kidney cancer risk was further intensified by an unhealthy lifestyle (RERI = 3.24, 95%CI=1.20-5.28). For women carrying HLA-A*24:02 and maintaining a poor lifestyle, the adjusted HR was 4.63 (95% CI = 2.69–7.96) and the 5-year cancer risk increased by 16% (95% CI = 8%–25%), compared to those in the lowest genetic and lifestyle risk group.

When examining the individual lifestyle factors, smoking showed the most consistent additive interactions with the implicated HLA alleles on lung cancer risk in men, whereas significant additive interactions were also observed for alcohol consumption and physical inactivity with relevant HLA alleles on kidney cancer risk in women (**Supplementary Table 20**).

### 3.5 Results of sensitivity analyses

When reconstructing HLA alleles into binary carrier status, a significant association between HLA-A*11:01 and kidney cancer risk was additionally observed in men (**Supplementary Table 21**); however, no interaction between this allele and lifestyle was identified on any cancer outcome. After applying the six-factor HLI score, the significant associations between lifestyle as well as the individual lifestyle factors in relation to all cancer outcomes were not materially changed (**Supplementary Tables 22**–**24**) and no additional significant interactions were found between this new version of lifestyle and any HLA allele for any cancer outcome. Additional adjustment for prevalent diseases and use of competing risk models did not identify any additional significant interactions and did not change the primary findings. When cancer incident cases were supplemented from hospital inpatient records and death registry, we observed a significant joint effect of being a DQB1*05:01 non-carrier and having a poor lifestyle to enhance lung cancer risk on an additive scale in men, with a RERI of 2.05 (95%CI=0.87-3.23), which was driven mainly by smoking (**Supplementary Table 25**). Among participants with the first-year incident cancers excluded, a significant joint effect of being a DQB1*05:01 non-carrier and having a poor lifestyle was observed for lung cancer risk in men with a RERI of 3.17 (95%CI=1.10-5.24), but interaction between A*24:02 and poor lifestyle on kidney cancer risk in women was not significant. After repeating the HLA–cancer association analyses using Cox proportional hazards models, the results were highly consistent with those from the logistic regression analyses, with all previously significant associations remaining significant with very similar effect estimates and no additional associations identified (**Supplementary Table 26**). Regarding the potential influence of the small number of cases in some joint-exposure strata on the observed significant additive interactions, bootstrap analyses with 1, 000 resamples yielded RERI estimates and 95% CIs highly consistent with those from the primary analyses (**Supplementary Table 27**). Influence diagnostics indicated minimal effects of individual cases, while sparse-cell case-deletion analyses resulted in only slight changes in the corresponding RERI estimates (**Supplementary Table 28**). Together, these findings support the robustness of the identified additive interactions. After additional adjustment for detailed smoking-related variables, all four previously identified additive interactions between HLA allele carriership and poor lifestyle on lung cancer risk in men remained statistically significant, although the corresponding RERI estimates were attenuated (**Supplementary Table 29**). Additional adjustment for reproductive and hormone-related variables yielded results largely unchanged from the primary analyses (**Supplementary Table 30**), with no new significant HLA–lifestyle interactions identified for female-specific cancers. The complete-case sensitivity analysis yielded results highly consistent with the primary analyses, with no changes in the identified significant HLA–lifestyle interactions and only minimal differences in the corresponding joint effect estimates (**Supplementary Table 31**).

## 4 Discussion

This study provides important epidemiological evidence that modifiable lifestyle factors and HLA polymorphisms may jointly influence site-specific cancer risk, which may help inform future risk-stratified cancer prevention strategies. First, we investigated novel, sex-stratified associations of common HLA alleles with site-specific cancers, revealing that in men, HLA-C*03:04 increased prostate cancer risk, while a cluster of correlated HLA alleles comprising HLA-B*08:01, DQA1*05:01, DQB1*02:01, DRB1*03:01, and DRB3*01:01 was linked to lung cancer; in women, HLA-A*24:02 and DPB1*04:01 were associated with kidney cancer and HLA-B*07:02 with endometrial cancer. Second, we confirmed that a composite poor lifestyle profile significantly elevated overall cancer risk by 38% in men and by 44% in women, and was associated with most common cancers. Most importantly, we provided the first epidemiologic evidence of positive additive HLA–lifestyle interactions: men carrying a cluster of AH8.1-related alleles (HLA-B*08:01, DQA1*05:01, DQB1*02:01, or DRB1*03:01) exhibited strong additive interactions with poor lifestyle, amplifying lung cancer risk, while women who were positive for HLA-A*24:02 showed amplified kidney cancer susceptibility when combined with poor lifestyle. These findings suggest that lifestyle factors and HLA polymorphisms may jointly influence cancer risk, although the underlying biological mechanisms remain to be established.

Existing literature supports a connection between an unhealthy lifestyle and higher cancer risk. A recent meta-analysis of 16 prospective cohort studies reported comparable overall cancer HRs of 1.41 (95%CI=1.31-1.52) for extreme lifestyle comparisons, with lung cancer showing the strongest lifestyle association in both sexes, a finding of similar strength to ours (12). When comparing the results from sex-stratified analyses, we observed different lifestyle–cancer association patterns among men and women. Associations between poor lifestyle and digestive cancers (particularly colorectal cancer) as well as lung cancer were generally stronger among men than women, with smoking, poor diet quality, and physical inactivity showing greater associations with these cancer outcomes in men than in women. In contrast, the association between poor lifestyle and kidney cancer was stronger among women, and poor lifestyle was also associated with female-specific cancers, including breast, ovarian and endometrial cancers, with unhealthy adiposity and smoking appearing to be major lifestyle contributors. These different association patterns observed among men and women further support the rationale for conducting sex-stratified analyses in this study. These differences may reflect variations in lifestyle exposure distributions, hormonal and reproductive factors, immune regulation, and cancer susceptibility pathways between men and women (33–35). Intriguingly, we observed negative associations of unhealthy lifestyle with skin cancer and prostate cancer, reflecting histological and methodological nuances: smoking could reduce basal cell carcinoma risk (RR = 0.85, 95%CI=0.75-0.96) while increasing squamous cell carcinoma risk (36), and prostate cancer associations may be confounded by screening behaviors and obesity’s dual effects on cancer aggressiveness (37, 38).

Our HLA-cancer associations found robust support in the literature. The identified lung cancer-associated alleles (HLA-B*08:01, DQA1*05:01, DQB1*02:01, or DRB1*03:01) are components of the AH8.1 ancestral haplotype, which has been previously linked to lung squamous carcinoma (OR = 1.30, 95%CI=1.18-1.42) (2) and many autoimmune disorders (39). Our findings further suggest that this AH8.1-related genetic background may interact with lifestyle factors in influencing lung cancer risk. Specifically, men carrying AH8.1-related marker status and having an intermediate or poor lifestyle displayed a substantially higher risk of lung cancer, with significant positive additive interactions observed. This suggests that immunogenetic background may not only confer inherent cancer susceptibility but may also exacerbate the detrimental impact of lifestyle factors, particularly by amplifying the effect of smoking on lung cancer among men. It should be noted that our AH8.1-related analyses were intended as supportive evidence for a shared AH8.1-related genetic signal rather than formal haplotype analyses. Future studies using phased HLA haplotypes may help clarify whether the canonical AH8.1 ancestral haplotype independently contributes to the risk of lung cancer and other cancers, and whether it interacts with lifestyle factors to influence cancer risk. Currently, the mechanisms through which these identified HLA loci contribute to lung cancer are not well understood. Experimental studies on other cancer types have suggested that HLA-DR-mediated signaling may activate ILK/AKT, FAK/PAX/AKT, and BRAF/ERK pathways, as well as PD-L1 expression, which enhance cell motility and invasiveness, promoting tumor growth and aggressiveness while contributing to immune escape (40), However, whether these mechanisms are relevant to lung carcinogenesis or to the specific HLA alleles identified in our study warrants further investigation. In addition, the interaction between HLA-DR and the TCR leads to the activation of c-Jun N-terminal kinase, a member of the MAPK family that has been shown to promote tumor proliferation, as demonstrated in non-small cell lung cancer (41). Similarly, HLA-DRB1*01:01’s association with non-Hodgkin lymphoma aligned with the reported ORs of 2.14 (95%CI=1.40-3.26) for follicular lymphoma, potentially through impaired B-cell clone control (42). For kidney cancer in women, HLA-A*24:02 may compromise γδT cell surveillance and engage NK inhibitory receptors (43), creating an immune-permissive microenvironment. Intriguingly, in our analyses of HLA–cancer associations, we observed opposite associations of the same HLA allele across different cancer types: HLA-DQB1*02:01 was associated with an increased risk of lung cancer but a reduced risk of prostate cancer, and HLA-DRB1*01:01 was associated with an increased risk of non-Hodgkin lymphoma but a reduced risk of lung cancer. Although the precise biological mechanisms underlying these cancer-specific HLA effects remain unclear, accumulating evidence suggests that immune surveillance mediated by HLA class II alleles may vary across tumor types due to differences in tumor-specific HLA class II expression, antigen presentation activity, mutational profiles, and immune microenvironments (44, 45). Therefore, the same HLA allele may enhance immune recognition in one cancer context while exerting limited or even opposite effects in another. Future studies integrating HLA peptide-binding profiles, tumor-specific antigen landscapes, and tumor immune microenvironment characteristics are warranted to elucidate these cancer-specific HLA effects.

In men, an AH8.1-related genetic signal represented by HLA-B*08:01, DQA1*05:01, DQB1*02:01 and DRB1*03:01 significantly amplified lung cancer risk when paired with poor lifestyle. Although smoking is an important contributor to both the lifestyle–lung cancer association and the observed HLA–lifestyle interaction in men, the interaction remained statistically significant after additional adjustment for detailed smoking-related variables. Moreover, significant additive interactions were also observed for alcohol consumption and physical inactivity for three of the four implicated alleles. Together, these findings suggest that smoking does not fully explain the observed interaction and support a broader lifestyle-profile interpretation rather than an HLA–smoking interaction alone. Nevertheless, we cannot exclude the possibility that sex differences in smoking patterns or other lifestyle behaviors contributed to the apparent male specificity of the association. Smoking may influence these interactions by both generating neoantigens and inducing chronic inflammation, which disrupts immune homeostasis (46). Alcohol may impair anti-tumor immune responses through multiple mechanisms, including alterations in immune-cell function and epigenetic regulation pathways, although its specific effects on HLA-mediated antigen presentation remain incompletely understood (47). Additionally, the cumulative burden of multiple unhealthy lifestyle factors can promote oxidative stress, insulin resistance, and metabolic disturbances, triggering epigenetic modifications in developing innate immune cells and ultimately impairing immune surveillance (6). Similarly, in women carrying HLA-A*24:02, a poor lifestyle aggravated kidney cancer risk, with alcohol consumption and physical inactivity being the most influential factors. HLA-A*24:02 may modulate immune responses by interacting with γδTCR or NK inhibitory receptors (43), although evidence in kidney cancer remains limited. Alcohol may contribute to kidney carcinogenesis through acetaldehyde-induced DNA damage and suppression of immune function, including impaired T cell activity and reduced NK cell effectiveness (48). Physical inactivity further elevates kidney cancer risk by increasing insulin and estrogen levels, which promote tumor growth, and by enhancing pro-inflammatory signaling pathways (49). However, this finding should be interpreted cautiously because the interaction between HLA-A*24:02 carriership and poor lifestyle was no longer statistically significant after excluding cancers developed in the first year of follow-up, and the number of kidney cancer cases was limited across the joint-exposure strata, particularly among HLA-A*24:02 carriers with a favorable lifestyle (n = 2). Therefore, this finding should be considered preliminary and warrants further validation. This study comprehensively evaluated both independent and interactive effects of composite lifestyle patterns and HLA polymorphisms across classical HLA alleles on extensive cancer outcomes. Key strengths included the prospective design, large sample size with integrated genetic and phenotypic data, and sex-stratified pan-cancer analyses, which allowed us to account for potential sex differences in lifestyle patterns and HLA-dependent immune surveillance. Findings remained robust across sensitivity analyses, including alternative lifestyle scoring schemes and HLA alleles analytical approaches. Several limitations should be acknowledged. First, self-reported lifestyle and covariates are susceptible to measurement error. Second, we focused on individual classical HLA alleles imputed from European reference datasets. Given the high degree of HLA polymorphisms and the complex linkage disequilibrium patterns, future studies should validate and extend our findings using whole-genome sequencing data across diverse ethnic groups and consider haplotype-based analyses. Additionally, this study focused on common classical HLA alleles with sufficient frequencies to ensure statistical power. Rare HLA alleles, which may also contribute to cancer susceptibility and could be enriched among individuals with specific cancer types, were not evaluated and warrant further investigation in future studies. Third, although family history of cancer was adjusted for in our analyses, the UK Biobank family cancer history questionnaire does not capture germline pathogenic variants in cancer predisposition genes, which may influence cancer susceptibility independently or modify associations between lifestyle, HLA polymorphisms, and cancer risk. Future studies incorporating germline variant information are warranted to further investigate their potential contributions to lifestyle–immune genetic interactions in cancer development. Beyond HLA polymorphisms and rare germline pathogenic variants, other cancer-related genetic factors, such as polygenic risk scores (PRS) capturing the cumulative effects of multiple common genetic variants, may also interact with lifestyle factors to influence cancer development. Future investigations incorporating a more comprehensive spectrum of cancer-related genetic determinants, including HLA variation, PRS, and rare pathogenic variants, may provide a deep understanding of gene–lifestyle interactions in cancer development. Fourth, sample size constraints limited investigation of HLA–lifestyle interactions in cancer subtypes or in further stratified subgroups. Larger studies will be needed to enable these analyses. Additionally, we used Cox-model-derived RERI to evaluate additive interactions. Future studies may consider absolute-risk-based approaches that account for competing risks to further validate and complement our findings. Fifth, residual or unmeasured confounding may persist. Finally, as the UK Biobank comprises predominantly European-ancestry individuals, generalizability to other ethnic populations requires further investigation.

## 5 Conclusion

In conclusion, individuals carrying specific HLA polymorphisms—particularly men carrying AH8.1-related alleles(HLA-B*08:01, DQA1*05:01, DQB1*02:01 or DRB1*03:01) and women carrying HLA-A*24:02—showed positive additive interactions between genetic susceptibility and unhealthy lifestyle patterns on lung cancer and kidney cancer, respectively. These observations underscore the importance of lifestyle modification as a core strategy for immune-informed cancer prevention, particularly among high-risk individuals carrying certain HLA polymorphisms. However, these findings require independent replication in larger and more ethnically diverse populations, particularly in non-European populations and in datasets with directly typed or sequenced HLA alleles. Importantly, integrative epidemiological and functional research is needed to elucidate how these specific HLA alleles confer heightened vulnerability when combined with adverse lifestyle factors.

## Data Availability

The original contributions presented in the study are included in the article/**Supplementary Material**. Further inquiries can be directed to the corresponding authors.

## References

[1] Cheong A Nagel ZD . Human variation in DNA repair, immune function, and cancer risk. Front Immunol. (2022) 13:899574. doi: 10.3389/fimmu.2022.899574 35935942 PMC9354717

[2] Ferreiro-Iglesias A Lesseur C McKay J Hung RJ Han Y Zong X . Fine mapping of MHC region in lung cancer highlights independent susceptibility loci by ethnicity. Nat Commun. (2018) 9:3927. doi: 10.1038/s41467-018-05890-2 30254314 PMC6156406

[3] Janeway CA Jr Travers P Walport M . The major histocompatibility complex and its functions. In: Immunobiology: The Immune System in Health and Disease, 5th Edition. Garland Science, New York (2001). Available online at: https://www.ncbi.nlm.nih.gov/books/NBK27156/ (Accessed April 6, 2026).

[4] Monye I Adelowo AB . Strengthening immunity through healthy lifestyle practices: Recommendations for lifestyle interventions in the management of COVID-19. Lifestyle Med. (2020) 1:e7. doi: 10.1002/lim2.7 38607804 PMC7646052

[5] Yoshida K Gowers KH Lee-Six H Chandrasekharan DP Coorens T Maughan EF . Tobacco smoking and somatic mutations in human bronchial epithelium. Nature. (2020) 578:266–72. doi: 10.1038/s41586-020-1961-1 31996850 PMC7021511

[6] Janeway CA Jr Travers P Walport M Shlomchik MJ . “ The major histocompatibility complex and its functions”. In: Immunobiology: The Immune System in Health and Disease. 5th ed. New York, NY: Garland Science. (2001).33330597

[7] Dartois L Fagherazzi G Boutron-Ruault M-C Mesrine S Clavel-Chapelon F . Association between five lifestyle habits and cancer risk: Results from the E3N cohort. Cancer Prev Res. (2014) 7:516–25. doi: 10.1158/1940-6207.capr-13-0325 24574508

[8] Sasazuki S Inoue M Iwasaki M Sawada N Shimazu T Yamaji T . Combined impact of five lifestyle factors and subsequent risk of cancer: The Japan Public Health Center Study. Prev Med. (2012) 54:112–6. doi: 10.1016/j.ypmed.2011.11.003 22155160

[9] Cerhan JR Potter JD Gilmore JM Janney CA Kushi LH Lazovich D . Adherence to the AICR cancer prevention recommendations and subsequent morbidity and mortality in the Iowa Women's Health Study cohort. Cancer epidemiology Biomarkers Prev A Publ Am Assoc For Cancer Research cosponsored by Am Soc Prev Oncol. (2004) 13:1114–20. doi: 10.1158/1055-9965.1114.13.7 15247121

[10] Romaguera D Vergnaud AC Peeters PH van Gils CH Chan DS Ferrari P . Is concordance with World Cancer Research Fund/American Institute for Cancer Research guidelines for cancer prevention related to subsequent risk of cancer? Results from the EPIC study. Am J Clin Nutr. (2012) 96:150–63. doi: 10.3945/ajcn.111.031674 22592101

[11] Zhu M Wang T Huang Y Zhao X Ding Y Zhu M . Genetic risk for overall cancer and the benefit of adherence to a healthy lifestyle. Cancer Res. (2021) 81:4618–27. doi: 10.1158/0008-5472.can-21-0836 34321244

[12] Zhang Y-B Pan X-F Chen J Cao A Zhang Y-G Xia L . Combined lifestyle factors, incident cancer, and cancer mortality: a systematic review and meta-analysis of prospective cohort studies. Br J Cancer. (2020) 122:1085–93. doi: 10.1038/s41416-020-0741-x 32037402 PMC7109112

[13] Sudlow C Gallacher J Allen N Beral V Burton P Danesh J . UK Biobank: an open access resource for identifying the causes of a wide range of complex diseases of middle and old age. PloS Med. (2015) 12:e1001779. doi: 10.1371/journal.pmed.1001779 25826379 PMC4380465

[14] Bycroft C Freeman C Petkova D Band G Elliott LT Sharp K . The UK Biobank resource with deep phenotyping and genomic data. Nature. (2018) 562:203–9. doi: 10.1038/s41586-018-0579-z 30305743 PMC6786975

[15] Wain LV Shrine N Miller S Jackson VE Ntalla I Artigas MS . Novel insights into the genetics of smoking behaviour, lung function, and chronic obstructive pulmonary disease (UK BiLEVE): a genetic association study in UK Biobank. Lancet Respir Med. (2015) 3:769–81. doi: 10.1016/s2213-2600(15)00283-0 26423011 PMC4593935

[16] Motyer A Vukcevic D Dilthey A Donnelly P McVean G Leslie S . Practical use of methods for imputation of HLA alleles from SNP genotype data. BioRxiv. (2016), 091009. doi: 10.1101/091009.

[17] Dilthey A Leslie S Moutsianas L Shen J Cox C Nelson MR . Multi-population classical HLA type imputation. PloS Comput Biol. (2013) 9:e1002877. doi: 10.1371/journal.pcbi.1002877 23459081 PMC3572961

[18] Imputation of Classical HLA Types From UK Biobank Genotype Data. Available online at: https://biobank.ndph.ox.ac.uk/showcase/refer.cgi?tk=5b2L2kUV1xPcbXCBFCTqSzda58NZP4O3437074&sid=182 (Accessed April 6, 2026).

[19] Shams-White MM Brockton NT Mitrou P Romaguera D Brown S Bender A . Operationalizing the 2018 World Cancer Research Fund/American Institute for Cancer Research (WCRF/AICR) cancer prevention recommendations: a standardized scoring system. Nutrients. (2019) 11:1572. doi: 10.3390/nu11071572 31336836 PMC6682977

[20] Wu E Ni JT Chen X Zhu ZH Xu HQ Tao L . Genetic risk, incident colorectal cancer, and the benefits of adhering to a healthy lifestyle: A prospective study using data from UK Biobank and FinnGen. Front Oncol. (2022) 12:894086. doi: 10.3389/fonc.2022.894086 36276143 PMC9582975

[21] Pirie K Peto R Reeves GK Green J Beral V . The 21st century hazards of smoking and benefits of stopping: a prospective study of one million women in the UK. Lancet. (2013) 381:133–41. doi: 10.1016/j.jvs.2013.03.032 23107252 PMC3547248

[22] Peduzzi P Concato J Kemper E Holford TR Feinstein AR . A simulation study of the number of events per variable in logistic regression analysis. J Clin Epidemiol. (1996) 49:1373–9. doi: 10.1016/s0895-4356(96)00236-3 8970487

[23] Hsieh FY Lavori PW . Sample-size calculations for the Cox proportional hazards regression model with nonbinary covariates. Controlled Clin trials. (2000) 21:552–60. doi: 10.1016/s0197-2456(00)00104-5 11146149

[24] Townsend P . Deprivation. J Soc Policy. (1987) 16:125–46. doi: 10.1017/s004727940002034141292463

[25] Butler-Laporte G Kreuzer D Nakanishi T Harroud A Forgetta V Richards JB . Genetic determinants of antibody-mediated immune responses to infectious diseases agents: a genome-wide and HLA association study. Open Forum Infect Dis. (2020) 7:ofaa450. doi: 10.1093/ofid/ofaa450 33204752 PMC7641500

[26] Gordon A Glazko G Qiu X Yakovlev A . Control of the mean number of false discoveries, Bonferroni and stability of multiple testing. Ann Appl Stat. (2007) 1:179–90. doi: 10.1214/07-aoas10242669646

[27] Carr PR Weigl K Jansen L Walter V Erben V Chang-Claude J . Healthy lifestyle factors associated with lower risk of colorectal cancer irrespective of genetic risk. Gastroenterology. (2018) 155:1805–15.e5. doi: 10.1053/j.gastro.2018.08.044 30201362 PMC6279591

[28] Desquilbet L Mariotti F . Dose-response analyses using restricted cubic spline functions in public health research. Stat Med. (2010) 29:1037–57. doi: 10.1002/sim.3841 20087875

[29] Hosmer DW Lemeshow S . Confidence interval estimation of interaction. Epidemiology. (1992) 3:452–6. doi: 10.1097/00001648-199209000-00012 1391139

[30] Austin PC . Absolute risk reductions and numbers needed to treat can be obtained from adjusted survival models for time-to-event outcomes. J Clin Epidemiol. (2010) 63:46–55. doi: 10.1016/j.jclinepi.2009.03.012 19595575

[31] Miller FW Chen W O'Hanlon TP Cooper RG Vencovsky J Rider LG . Genome-wide association study identifies HLA 8.1 ancestral haplotype alleles as major genetic risk factors for myositis phenotypes. Genes Immun. (2015) 16:470–80. doi: 10.1038/gene.2015.28 26291516 PMC4840953

[32] Stahl E Roda G Dobbyn A Hu J Zhang Z Westerlind H . Collagenous colitis is associated with HLA signature and shares genetic risks with other immune-mediated diseases. Gastroenterology. (2020) 159:549–61.e8. doi: 10.1053/j.gastro.2020.04.063 32371109 PMC7483815

[33] Haupt S Caramia F Klein SL Rubin JB Haupt Y . Sex disparities matter in cancer development and therapy. Nat Rev Cancer. (2021) 21:393–407. doi: 10.1038/s41568-021-00348-y 33879867 PMC8284191

[34] Zhao J Wang Q Tan AF Loh CJL Toh HC . Sex differences in cancer and immunotherapy outcomes: the role of androgen receptor. Front Immunol. (2024) 15:1416941. doi: 10.3389/fimmu.2024.1416941 38863718 PMC11165033

[35] Xiao T Lee J Gauntner TD Velegraki M Lathia JD Li Z . Hallmarks of sex bias in immuno-oncology: mechanisms and therapeutic implications. Nat Rev Cancer. (2024) 24:338–55. doi: 10.1038/s41568-024-00680-z 38589557

[36] Arafa A Mostafa A Navarini AA Dong JY . The association between smoking and risk of skin cancer: a meta-analysis of cohort studies. Cancer causes control CCC. (2020) 31:787–94. doi: 10.1007/s10552-020-01319-8 32458137

[37] Allott EH Masko EM Freedland SJ . Obesity and prostate cancer: weighing the evidence. Eur Urol. (2013) 63:800–9. doi: 10.1016/j.eururo.2012.11.013 23219374 PMC3597763

[38] Huncharek M Haddock KS Reid R Kupelnick B . Smoking as a risk factor for prostate cancer: a meta-analysis of 24 prospective cohort studies. Am J Public Health. (2010) 100:693–701. doi: 10.2105/ajph.2008.150508 19608952 PMC2836346

[39] Candore G Lio D Romano GC Caruso C . Pathogenesis of autoimmune diseases associated with 8.1 ancestral haplotype: effect of multiple gene interactions. Autoimmun Rev. (2002) 1:29–35. doi: 10.1016/s1568-9972(01)00004-0 12849055

[40] Costantini F Barbieri G . The HLA-DR mediated signalling increases the migration and invasion of melanoma cells, the expression and lipid raft recruitment of adhesion receptors, PD-L1 and signal transduction proteins. Cell Signalling. (2017) 36:189–203. doi: 10.1016/j.cellsig.2017.05.008 28495591

[41] Okada M Shibuya K Sato A Seino S Watanabe E Suzuki S . Specific role of JNK in the maintenance of the tumor-initiating capacity of A549 human non-small cell lung cancer cells. Oncol Rep. (2013) 30:1957–64. doi: 10.3892/or.2013.2655 23912840

[42] Wang SS Abdou AM Morton LM Thomas R Cerhan JR Gao X . Human leukocyte antigen class I and II alleles in non-Hodgkin lymphoma etiology. Blood. (2010) 115:4820–3. doi: 10.1182/blood-2010-01-266775 20385791 PMC2890176

[43] Mihaylova A Mihailova S Naumova E . NK cell receptor and MHC gene polymorphisms, potential relevance in Malignancies. Curr Cancer Ther Rev. (2008) 4:130–6. doi: 10.2174/157339408784310070

[44] Yang K Halima A Chan TA . Antigen presentation in cancer — mechanisms and clinical implications for immunotherapy. Nat Rev Clin Oncol. (2023) 20:604–23. doi: 10.1038/s41571-023-00789-4 37328642

[45] Seliger B Kloor M Ferrone S . HLA class II antigen-processing pathway in tumors: Molecular defects and clinical relevance. Oncoimmunology. (2017) 6:e1171447. doi: 10.1080/2162402x.2016.1171447 28344859 PMC5353941

[46] Addissouky TA El Sayed IET Ali MM Wang Y El Baz A Elarabany N . Oxidative stress and inflammation: elucidating mechanisms of smoking-attributable pathology for therapeutic targeting. Bull Natl Res Cent. (2024) 48:16. doi: 10.1186/s42269-024-01174-638164791

[47] Rumgay H Murphy N Ferrari P Soerjomataram I . Alcohol and cancer: epidemiology and biological mechanisms. Nutrients. (2021) 13:3173. doi: 10.3390/nu13093173 34579050 PMC8470184

[48] Simet SM Sisson JH . Alcohol's effects on lung health and immunity. Alcohol Res. (2015) 37:199–209. doi: 10.35946/arcr.v37.2.05 26695745 PMC4590617

[49] Lynch BM Leitzmann MF . An evaluation of the evidence relating to physical inactivity, sedentary behavior, and cancer incidence and mortality. Curr Epidemiol Rep. (2017) 4:221–31. doi: 10.1007/s40471-017-0119-730311153

