# Supplementary material for: Independent and joint effects of lifestyle, human leukocyte antigen polymorphisms on cancer risk: findings from the UK Biobank
Source: Front Immunol. 2026 Sep 11;17:1899135. doi: 10.3389/fimmu.2026.1899135 (PMC13612265; doi:10.3389/fimmu.2026.1899135)
Supplement: Supplementary file 1 [file DataSheet1.pdf]

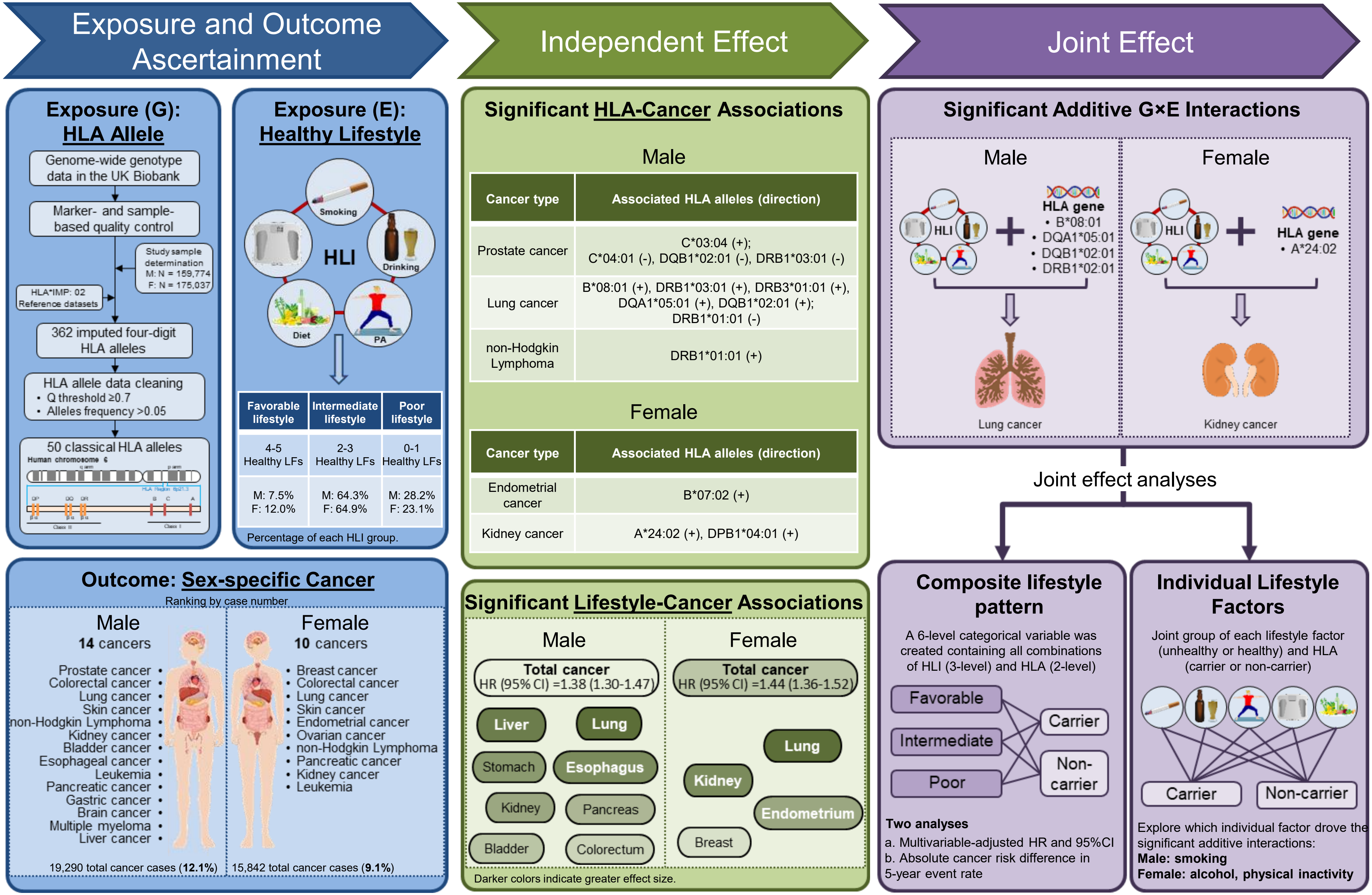

Supplementary Figure S1. Flowchart of the study design. Abbreviations: CI, confidence interval; E, environment; F, female; G, gene; HLA, human leukocyte antigen; HLI, Healthy Lifestyle Index; HR, hazard ratio; LF, lifestyle factor; M, male; PA, physical activity

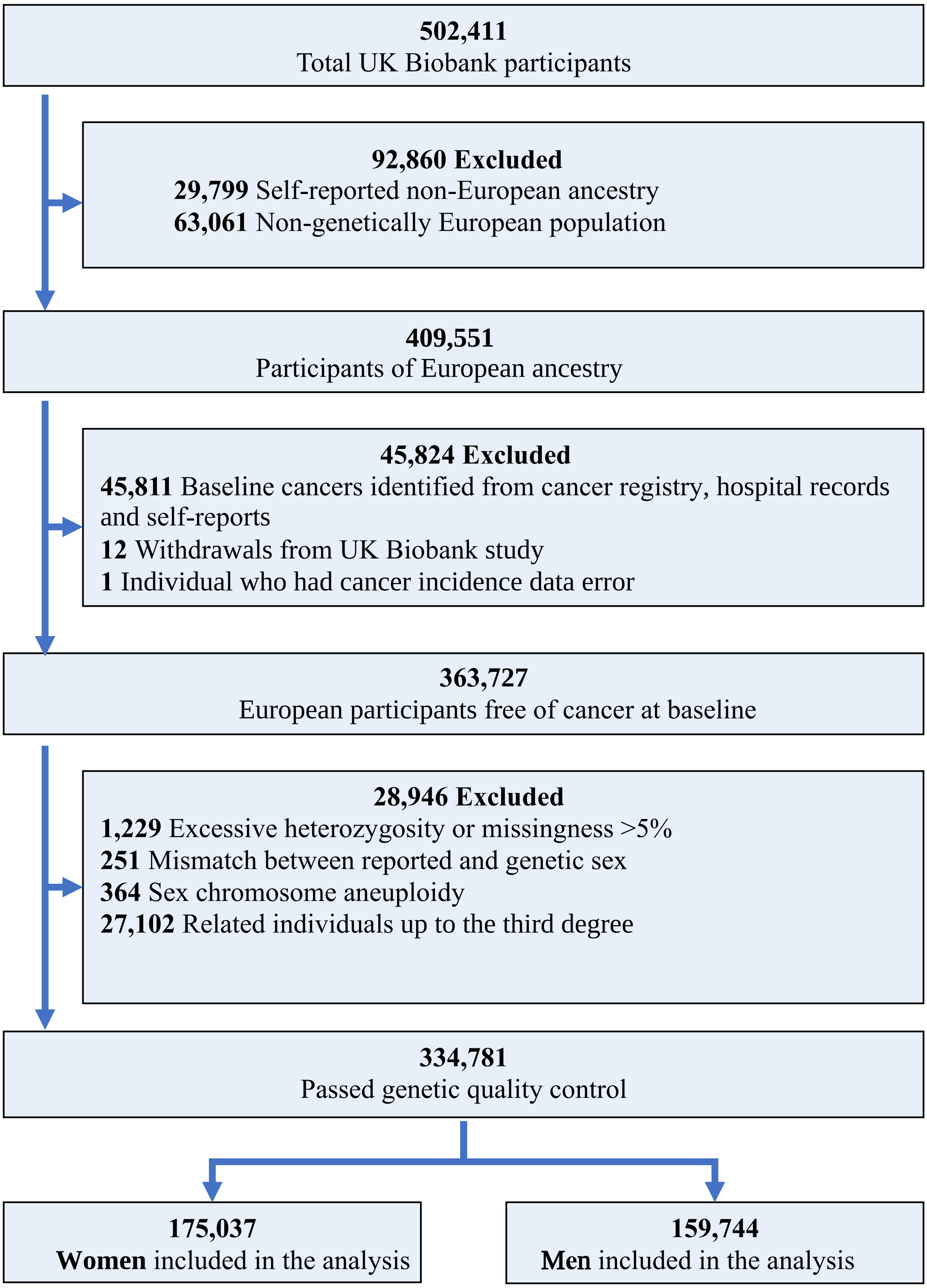

Supplementary Figure S2. Flowchart of study population selection in the UK Biobank study

A MEN

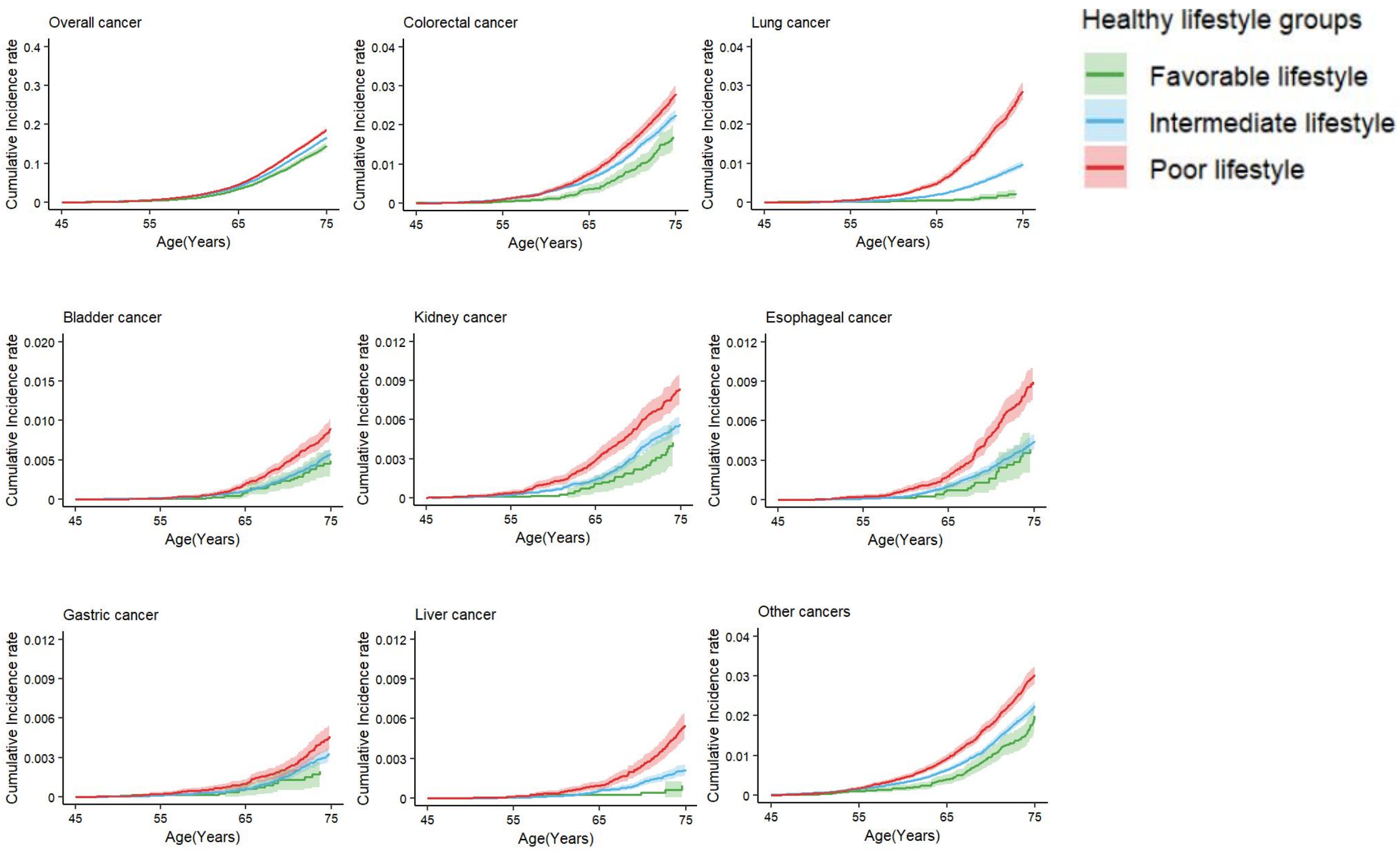

B WOMEN

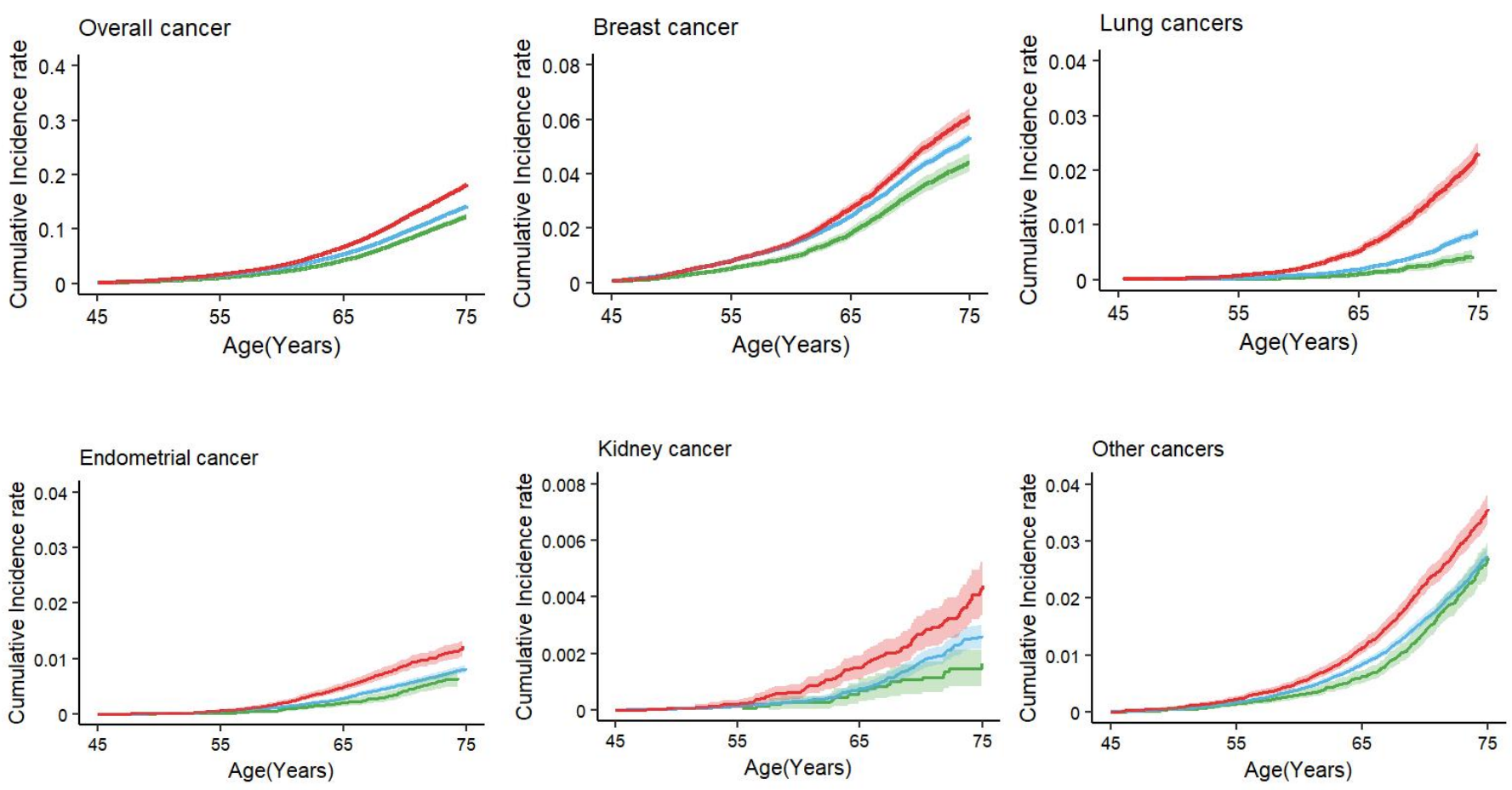

Supplementary Figure S3. Cumulative cancer incidence curve by age 75 for the selected cancer outcomes according to lifestyle groups assessed with Healthy Lifestyle Index in the UK Biobank study (A)among men and (B)among women. The cumulative incidence rates were estimated using the weighted cohort by inverse probability weighting, adjusted for baseline age, height,assessment center,Townsend deprivation index at recruitment, and family history of cancer.These selected cancers were significantly associated with the Healthy Lifestyle Index score.

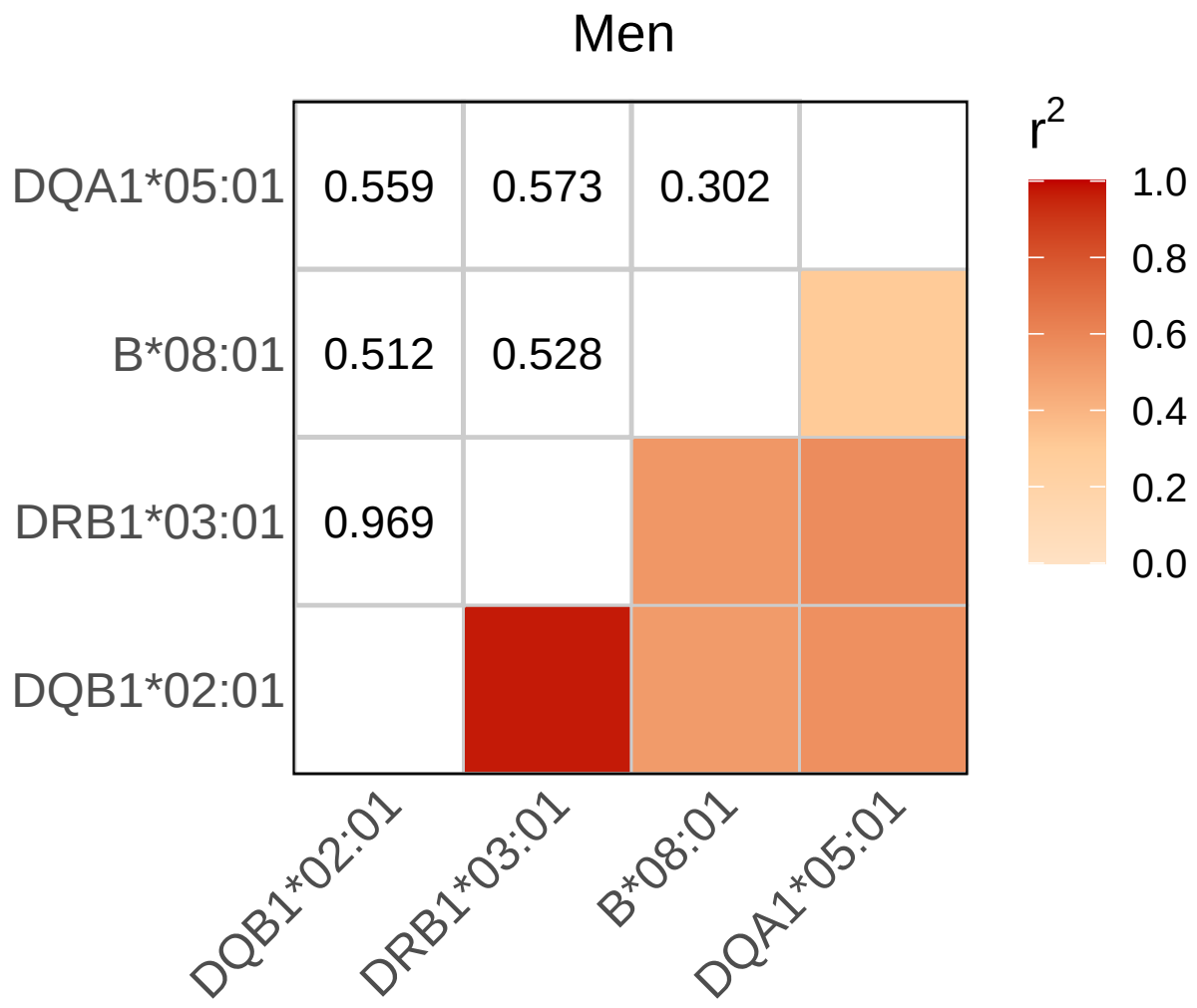

Supplementary Figure S4. Pairwise linkage disequilibrium (r^2) among four HLA alleles involved in significant HLA–lifestyle interactions for lung cancer in men and related to the AH8.1 ancestral haplotype in the UK Biobank. The four HLA alleles (HLA-B*08:01, HLA-DQA1*05:01, HLA-DQB1*02:01, and HLA-DRB1*03:01) were identified as significantly associated with lung cancer and showing significant HLA–lifestyle interactions in men and are recognized components or markers of the AH8.1 ancestral haplotype. Pairwise r^2 values, calculated from allelic dosages using Pearson correlation coefficients, ranged from 0.30 to 0.97, supporting these alleles as a shared AH8.1-related haplotypic signal rather than independent associations.

Cancer types	Joint group	Cases No.	Incidence rates per 1000 person-years	Multivariable-adjusted HR (95%CI)	Additive interaction	
					RERI (95%CI)	*P* value
Men						
**Lung cancer**	**AH8.1-**					
	Favorable lifestyle	10	0.13	Ref		
	Intermediate	307	0.46	3.68 (1.96-6.91)*		
	Poor lifestyle	367	1.29	8.84 (4.72-16.58)*		
	**AH8.1+**					
	Favorable lifestyle	11	0.17	1.34 (0.57-3.16)		
	Intermediate	284	0.52	4.16 (2.22-7.83)*	0.14 (-1.01-1.29)	0.41
	Poor lifestyle	396	1.68	11.52 (6.15-21.60)*	2.34 (0.48-4.19)	0.007

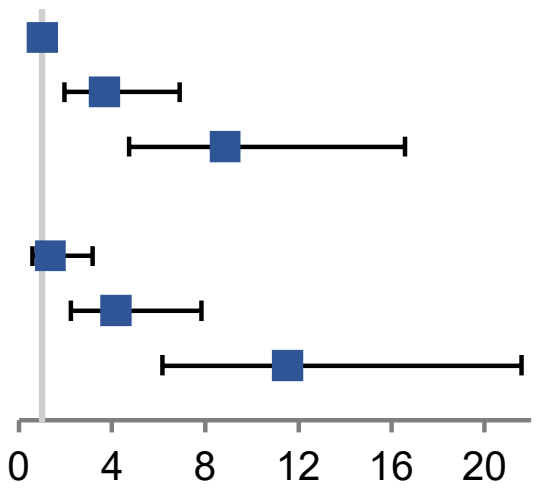

Supplementary Figure S5. Significant interactions and joint effects between AH8.1-related marker status and lifestyle groups on lung cancer risk in men. AH8.1-related marker status was defined using a simplified marker-based definition based on the presence of HLA-A*01, HLA-B*08, and HLA-DRB1*03. This definition was used as a proxy for the shared AH8.1-related genetic signal identified in the allele-based analyses and should not be interpreted as the canonical 8.1 ancestral haplotype itself. Individuals without AH8.1-related marker status (i.e.,carrying none of the three defining markers) and with a favorable lifestyle were used as the reference group in the joint-effect analyses. HRs and 95% CIs were estimated using multivariable-adjusted Cox proportional hazards models adjusted for baseline age, height, assessment center, Townsend deprivation index, family history of cancer,and population structure (top five genetic principal components). Abbreviations: AH8.1,ancestral haplotype 8.1; CI,confidence interval; HLA, human leukocyte antigen; HR, hazard ratio; RERI, relative excess risk due to interaction.

* Statistical significance in the joint effect analyses (P<0.05)
