# Supplementary material for: Independent and joint effects of lifestyle, human leukocyte antigen polymorphisms on cancer risk: findings from the UK Biobank
Source: Front Immunol. 2026 Sep 11;17:1899135. doi: 10.3389/fimmu.2026.1899135 (PMC13612265; doi:10.3389/fimmu.2026.1899135)
Supplement: Supplementary file 2 [file DataSheet2.pdf]

Supplementary Methods and Tables

Supplementary methods. covariates assessment and categorization

Age at recruitment

Age at recruitment (Field 21003) was derived by UK Biobank based on date of birth and date of attending the assessment center and referred to the age on the day the participant attended an Assessment Center, treated as a continuous variable in our analyses.

Townsend deprivation index

Townsend deprivation index (Field 22189) was calculated prior to participant joining UK Biobank based on the preceding national census output areas in which participants' postcodes were located. It was an area-based proxy measure for material deprivation composed of data on car ownership, household overcrowding, household owner-occupation, and unemployment. Negative values indicate relative affluence with low-level deprivation, while positive values indicate areas with high-level deprivation. It was treated as a continuous variable in our analyses.

Educational level

Education was assessed on the baseline touchscreen questionnaire. Participants were asked 'Which of the following qualifications do you have?' (Field 6138). Education qualification contained four groups in our analyses: 'College or university degree/vocational qualification' (i.e., the highest qualification level which included 'college or university degree', 'NVQ or HND or HNC or equivalent', and 'other professional qualifications eg.: nursing, teaching'); 'National examination at age 17-18'(i.e., the intermediate qualification level including 'A levels/AS levels or equivalent'); 'National examination at age 16'(the lowest qualification level including 'O levels/GCSEs or equivalent', and 'CSEs or equivalent'; and 'Unknown'(including 'none of the above', 'prefer not to answer', and missing).

Height

Height referred to standing height (Field 50) which was measured by trained staff using a Seca 202 device during the interviews. Height was treated as a continuous variable in our analyses with units of centimeters.

Family history of cancer

Family history of cancer was measured based on whether the mother or father suffered from breast cancer, bowel cancer, lung cancer or prostate cancer (Field 20107 and 20110). Participants were asked, "Has/did your father ever suffers from? (You can select more than one answer)" as well as "Has/did your mother ever suffers from? (You can select more than one answer)" for different illnesses shown on the touchscreen at the Assessment Centre. Family history of cancer was considered only for related parents, not adopted parents, grandparents, or collateral relatives. A binary variable was used in the analyses, with 1 representing a family history of cancer and 0 representing none.

Prevalent diseases

Information regarding prevalent diseases at enrollment was obtained from (1) the self-report collected via an in-person interview or touchscreen questionnaire (2) self-reported medication use for type-2 diabetes, dyslipidemia, hypertension, cardiovascular disease and respiratory disease (3) disease diagnoses based on hospital in-patient records and recorded using the ICD-9 or ICD-10 coding system. Details were shown in the Supplementary Table S4.

Detailed smoking variables

Pack-years of smoking (Field 20161) were used to quantify cumulative tobacco exposure. Never smokers and participants with missing pack-years were coded as 0. Age at smoking cessation (Field

2897) and whether the participant had ever stopped smoking for 6 months or longer (Field 2907) were also extracted from the baseline touchscreen questionnaire. Smoking cessation duration was calculated as the difference between age at recruitment and age at smoking cessation among former smokers who reported having stopped for at least 6 months. These detailed smoking variables were included as covariates in sensitivity analyses of the HLA-lifestyle interaction on lung cancer risk.

Reproductive and hormonal factors

For female-specific cancers (breast, endometrial, and ovarian), reproductive and hormonal variables were additionally considered. Age at menarche (Field 2714), menopausal status (Field 2724), number of live births (Field 2734), age at first live birth (Field 2754), and ever use of oral contraceptive pills (Field 2784) were extracted from the touchscreen questionnaire. Menopausal status was categorized as postmenopausal (yes) versus premenopausal or uncertain (no). Number of live births and age at first live birth were treated as continuous variables. Age at menarche was treated as continuous. Ever use of oral contraceptive pills was treated as binary. These variables were included in sensitivity analyses of lifestyle associations and HLA-lifestyle interactions for female-specific cancers.

Supplementary Table S1. Frequencies of HLA Class I and Class II alleles included in the UK Biobank study

Class I (N=18)			Class II (N=32)		
HLA-	Allele frequency (%) [a]		HLA-	Allele frequency (%) [a]	
	Men	Women		Men	Women
A*01:01	19.42	19.41	DPA1*01:03	81.49	81.54
A*02:01	27.62	27.54	DPA1*02:01	14.69	14.62
A*03:01	14.49	14.48	DPB1*01:01	5.98	5.93
A*11:01	6.01	6.01	DPB1*02:01	11.09	11.05
A*24:02	7.35	7.38	DPB1*03:01	9.89	10.03
B*07:02	14.82	14.78	DPB1*04:01	43.89	43.80
B*08:01	14.47	14.49	DPB1*04:02	10.81	10.76
B*15:01	6.24	6.23	DQA1*01:01	14.06	14.21
B*40:01	5.59	5.59	DQA1*01:02	19.25	19.07
B*44:02	11.24	11.20	DQA1*01:03	5.50	5.51
B*44:03	5.85	5.90	DQA1*02:01	14.55	14.68
C*03:03	5.64	5.57	DQA1*03:01	20.41	20.31
C*03:04	8.03	8.04	DQA1*05:01	23.14	23.17
C*04:01	8.24	8.28	DQB1*02:01	14.89	15.04
C*05:01	11.38	11.33	DQB1*02:02	10.35	10.47
C*06:02	9.03	8.96	DQB1*03:01	17.57	17.48
C*07:01	17.65	17.71	DQB1*03:02	10.44	10.30
C*07:02	15.81	15.80	DQB1*03:03	5.23	5.26
			DQB1*05:01	11.89	12.07
			DQB1*06:02	14.38	14.15
			DQB1*06:03	5.13	5.19
			DRB1*01:01	9.31	9.45
			DRB1*03:01	14.81	14.96
			DRB1*04:01	11.46	11.36
			DRB1*07:01	14.53	14.65
			DRB1*13:01	5.06	5.09
			DRB1*15:01	14.60	14.36
			DRB3*01:01	16.71	16.84
			DRB3*02:02	13.38	13.32
			DRB4*01:01	8.22	8.30
			DRB4*01:03	25.93	25.85
			DRB5*01:01	14.55	14.31

Abbreviations: HLA, human leukocyte antigen.

[a] The allele frequency was calculated in men and women separately by simple counting methods where the sum of all allele frequencies at each locus was 1. This table only presents the 50 alleles with frequencies ≥5%. We extracted each participant's allelic dosage by thresholding posterior allele probability at 0.7 as suggested by UK Biobank.

Supplementary Table S2. Grading criteria for Healthy Lifestyle Index (HLI) and definitions of lifestyle categories in the UK Biobank study

Individual lifestyle factor	Grading criteria for HLI in the main analysis		Score	Field ID	Grading criteria in the sensitivity analysis (Six-factor HLI)	
**Smoking**	Healthy	Non-current smokers: never smokers OR smokers who had quit >30 y	1	20116, 2897	Ideal	Never smokers or smokers who had quit >30 y
	Unhealthy	Current smokers: current smoking OR smokers who had quit ≤30 y	0		Intermediate	Past smokers who had quit ≤30 y
					Poor	Current smoking
**Alcohol consumption**	Healthy	Never drinkers	1	20117, 1558, 1568, 1578, 1588, 1598, 1608, 5364, 4407, 4418, 4429, 4440, 4451, 4462	Ideal	Never drinkers
	Unhealthy	Ever drinkers	0		Intermediate	Past drinkers or current drinkers with alcohol consumption ≤28 g/day for men and ≤14 g/day for women
					Poor	Alcohol consumption >28 g/day for men and >14 g/day for women
**Physical activity**	Healthy	≥150 min/wk moderate-to-vigorous PA	1	22038, 22039	Ideal	≥150 min/wk moderate-to-vigorous PA
	Unhealthy	<150 min/wk moderate to vigorous PA	0		Intermediate	75-<150 PA
					Poor	<75 min/wk moderate-to-vigorous PA
**Weight**	Healthy	BMI 18.5-<30 kg/m² OR WC<102 cm for men<br>BMI 18.5-<30 kg/m² OR WC<88 cm for women	1	21001, 48	Ideal	BMI 18.5-<30 kg/m² AND WC <94 cm for men<br>BMI 18.5-<30 kg/m² AND WC <80 cm for women
	Unhealthy	BMI <18.5 or ≥30 kg/m² AND WC≥102 cm for men<br>BMI <18.5 or ≥30 kg/m² AND WC≥88 cm for women	0		Intermediate	Between the Ideal and the Poor
					Poor	BMI<18.5 or ≥30 kg/m² AND WC≥102 cm for men<br>BMI<18.5 or ≥30 kg/m² AND WC≥88 cm for women
**Diet**	Healthy	≥4 of the 6 dietary recommendations<br>① Fruits: ≥ 4 servings/day<br>② Vegetables: ≥ 12 tablespoons/day<br>③ Fish: ≥2 times/wk<br>④ Processed meat: ≤ 1 times/wk<br>⑤ Unprocessed red meat : ≤ 1.5 times/wk<br>⑥ Whole grains: ≥ 3 servings/wk	1	1289, 1299, 1309, 1319, 1329, 1339, 1349, 1369, 1379, 1389, 1438, 1448, 1458, 1468	I. Fruits and vegetables	
					Ideal	≥400 g/day
	Unhealthy	<4 of the 6 dietary recommendations	0		Intermediate	200-<400 g/day
					Poor	<200 g/day
					II. Red meat and processed meat	
					Ideal	Unprocessed red meat ≤3 times/wk and processed meat ≤1 times/wk
					Intermediate	Unprocessed red meat ≤3 times/wk and processed meat >1-3 times/wk
					Poor	Unprocessed red meat >3 times/wk OR processed meat >3 times/wk
**Categorization of lifestyle groups**					**Categorization of lifestyle groups**	
Favorable	Adherence to 4-5 healthy lifestyle factors				Favorable	≥4 ideal lifestyle factors
Intermediate	Adherence to 2-3 healthy lifestyle factors				Intermediate	Between the Favorable and the Poor
Poor	Adherence to 0-1 healthy lifestyle factor				Poor	≥4 poor lifestyle factors

Abbreviations: BMI, body mass index; HLI, Healthy Lifestyle Index; PA, physical activity; WC, waist circumference; wk, week; y, year.

Supplementary Table S3. Information of included sex-specific cancers in the UK Biobank study

Cancer types [a]	Incident Cases No.	Incidence rates per 1000 person-years	ICD-10
**Men**			
**Prostate cancer**	7846	4.16	C61
**Colorectal cancer**	2309	1.22	C18.0, C18.1, C18.2, C18.3, C18.4, C18.5, C18.6, C18.7, C18.8, C18.9, C19, C20
**Lung cancer**	1377	0.73	C34.0, C34.1, C34.2, C34.3, C34.8, C34.9
**Skin cancer**	1046	0.55	C43.0, C43.1, C43.2, C43.3, C43.4, C43.5, C43.6, C43.7, C43.9
**non-Hodgkin lymphoma**	715	0.38	C82.0, C82.1, C82.2, C82.3, C82.9, C83.0, C83.1, C83.3, C83.4, C83.5, C83.7, C83.8, C85.1, C85.7, C85.9
**Kidney cancer**	620	0.33	C64
**Bladder cancer**	605	0.32	C67.0, C67.1, C67.2, C67.3, C67.4, C67.5, C67.6, C67.7, C67.8, C67.9
**Esophageal cancer**	535	0.28	C15.0, C15.1, C15.2, C15.3, C15.4, C15.5, C15.8, C15.9
**Leukemia**	521	0.28	C91.0, C91.1, C91.3, C91.4, C91.5, C91.9, C92.0, C92.1, C92.4, C92.5, C92.7, C92.8, C92.9, C93.0, C93.1, C94.0, C94.6, C95.0, C95.9
**Pancreatic cancer**	456	0.24	C25.0, C25.1, C25.2, C25.3, C25.4, C25.7, C25.8, C25.9
**Gastric cancer**	331	0.18	C16.0, C16.1, C16.2, C16.3, C16.4, C16.5, C16.6, C16.8, C16.9
**Brain cancer**	321	0.17	C71.0, C71.1, C71.2, C71.3, C71.4, C71.5, C71.6, C71.7, C71.8, C71.9
**Multiple myeloma**	313	0.17	C90.0
**Liver cancer**	280	0.15	C22.0, C22.1, C22.3, C22.4, C22.7, C22.9
**Other cancers** [b]	2015	1.07	
**Overall cancer** [c]	19290	10.23	C00.0-C97 except C44.0-C44.9
**Women**			
**Breast cancer**	6131	3.25	C50.0, C50.1, C50.2, C50.3, C50.4, C50.5, C50.6, C50.8, C50.9
**Colorectal cancer**	1640	0.87	C18.0, C18.1, C18.2, C18.3, C18.4, C18.5, C18.6, C18.7, C18.8, C18.9, C19, C20
**Lung cancer**	1177	0.62	C34.0, C34.1, C34.2, C34.3, C34.8, C34.9
**Skin cancer**	994	0.53	C43.0, C43.1, C43.2, C43.3, C43.4, C43.5, C43.6, C43.7, C43.9
**Endometrial cancer**	985	0.52	C54.1
**Ovarian cancer**	638	0.34	C56
**non-Hodgkin lymphoma**	606	0.32	C82.0, C82.1, C82.2, C82.3, C82.9, C83.0, C83.1, C83.3, C83.4, C83.5, C83.7, C83.8, C85.1, C85.7, C85.9
**Pancreatic cancer**	368	0.20	C25.0, C25.1, C25.2, C25.3, C25.4, C25.7, C25.8, C25.9
**Kidney cancer**	328	0.17	C64
**Leukemia**	326	0.17	C91.0, C91.1, C91.3, C91.4, C91.5, C91.9, C92.0, C92.1, C92.4, C92.5, C92.7, C92.8, C92.9, C93.0, C93.1, C94.0, C94.6, C95.0, C95.9
**Other cancers** [b]	2649	1.41	
**Overall cancer** [c]	15842	8.40	C00.0-C97 except C44.0-C44.9

Abbreviations: HLA, human leukocyte antigen; ICD, International Classification of Diseases.

[a] Cancers with no less than 280 incident cases in men or women were selected as studied outcomes based on sample size calculations for the associations of HLA alleles and Healthy Lifestyle Index score with cancer incidence. There are 14 cancer types for men and 10 for women.

[b] In men, other cancers included all malignant cancers except the top 14 listed in the table which were studied in this study, of which the top three were mesothelioma (12.21%), oral cancer (11.81%), and tonsil cancer (6.50%). Among women, other cancers included all malignant cancers except the top 10 ranked in the table which were studied in this study, with the top three being multiple myeloma (8.42%), brain cancer (8.23%), and esophageal cancer (7.17%).

[c] Overall cancer was referred to having any one of 342 types of malignant cancers in the UK Biobank.

Supplementary Table S4. Codes used in the UK Biobank study to define prevalent diseases

Prevalent diseases	UKB field IDs and values for self-reported medical conditions and medication use	ICD codes for hospital inpatient records (fields 41270 and 41271)	
		ICD-9	ICD-10
**Type 2 diabetes**	2443(1), 2976, 6153(3), 6177(3), 20002(1220, 1222, 1223)	250	E10-E14
**Dyslipidemia**	20002 (1473), 6153 (1), 6177 (1)	272	E78
**Hypertension**	6150 (4), 20002 (1065,1072), 6153 (2), 6177 (2)	401-405	I10-I13, I15, O10
**Cardiovascular disease**	6150 (1,2,3), 20002 (1074,1075,1081,1583,1086, 1491)	410-414, 430-434, 436	I20-I25, I60-I64
**Respiratory disease**	20002 (1112, 1113, 1472), 6152 (6)	491, 492, 496	J40-J44, J47
**Digestive disease**	20002 (1134, 1135, 1136, 1137, 1138, 1139, 1140, 1141, 1142, 1143, 1154, 1155, 1156, 1157, 1158, 1159, 1160, 1161, 1162, 1163, 1164, 1165, 1190, 1191, 1400, 1432, 1456, 1457, 1458, 1459, 1460, 1461, 1462, 1463, 1475, 1501, 1503, 1504, 1505, 1506, 1509, 1510, 1511, 1512, 1513, 1562, 1599, 1600, 1601, 1602, 1603, 1604)	520-579	K00-K93
**Chronic kidney disease**	20002 (1427 ,1607, 1192, 1193, 1194, 1519, 1520)	75315, 584, 585, 586, 587, 588;	I12.0, I13.1, I13.2, N028, N03, N06, N08, N11, N12, N13, N14, N15, N16, N17, N18, N19, N20, N21, E112, E85.3, Q60.1, Q611, Q612, Q613, T82.4, T86.1, Y60.2, Y61.2, Y62.2, Y84.1, Z49.0, Z49.1, Z49.2, Z94.0, Z99.2

Abbreviations: ICD, International Classification of Diseases.

Supplementary Table S5. Associations between AH8.1-related marker status and incident lung cancer risk in men

**HLA allele**	**AF (%)** [a]	**Multivariable-adjusted OR (95%CI)** [b]	***P* value**
A1-B8-DR3 [c]			
AH8.1-related marker status-	54.9	Ref	Ref
AH8.1-related marker status+	45.1	1.23 (1.11-1.37)	**1.46E-04**[*]

Abbreviations: HLA, human leukocyte antigen; AF, allele frequency; OR: odds ratio

[a] The allele frequency was calculated in men by simple counting methods where the sum of all allele frequencies at each locus was 1

[b] Multivariable-adjusted ORs and 95%CIs were computed from the multivariable-adjusted logistic regression model adjusted for age at recruitment, assessment center, and population structure (top 5 principal components of the genotypes). Individuals without AH8.1-related marker status (i.e., carrying none of the three defining markers) were used as the reference group.

[c] AH8.1-related marker status was defined by the presence of HLA-A*01, HLA-B*08 and HLA-DRB1*03 and was used as a marker-based proxy for the AH8.1-related genetic signal identified in the allele-level analyses. It does not represent formal identification of the canonical AH8.1 ancestral haplotype.

* $P<0.001$ was considered statistical significance to be consistent with HLA allele-cancer analyses

Supplementary Table S6. Multivariable-adjusted associations between each HLA allele and incident risk of overall cancer

	Men		Women	
HLA alleles	Adjusted OR (95%CI) [a]	*P* value [b]	Adjusted OR (95%CI) [a]	*P* value [b]
**A*01:01**	1.00 (0.97-1.02)	0.7914	1.01 (0.98-1.05)	0.3972
**A*02:01**	1.01 (0.98-1.03)	0.5753	0.99 (0.96-1.03)	0.6858
**A*03:01**	0.98 (0.95-1.01)	0.2570	0.99 (0.95-1.03)	0.6128
**A*11:01**	1.03 (0.98-1.08)	0.2333	0.98 (0.93-1.03)	0.3576
**A*24:02**	1.00 (0.96-1.04)	0.9564	0.99 (0.94-1.04)	0.6525
**B*07:02**	1.01 (0.97-1.04)	0.7431	1.04 (1.00-1.08)	0.0318
**B*08:01**	1.01 (0.98-1.04)	0.5249	1.03 (0.99-1.07)	0.1421
**B*15:01**	1.03 (0.98-1.08)	0.2015	0.96 (0.92-1.01)	0.1555
**B*40:01**	1.01 (0.96-1.06)	0.6494	1.00 (0.95-1.06)	0.9224
**B*44:02**	0.99 (0.96-1.03)	0.7542	0.97 (0.93-1.01)	0.1764
**B*44:03**	0.97 (0.93-1.02)	0.2047	1.01 (0.96-1.07)	0.5781
**C*03:03**	1.02 (0.97-1.07)	0.3819	0.95 (0.90-1.00)	0.0690
**C*03:04**	1.03 (0.99-1.07)	0.1919	1.01 (0.96-1.05)	0.7500
**C*04:01**	0.98 (0.94-1.02)	0.2361	0.96 (0.91-1.00)	0.0538
**C*05:01**	0.99 (0.96-1.02)	0.5457	0.98 (0.94-1.02)	0.4127
**C*06:02**	1.02 (0.99-1.06)	0.2170	1.03 (0.98-1.07)	0.2590
**C*07:01**	1.01 (0.98-1.04)	0.6111	1.01 (0.98-1.05)	0.4340
**C*07:02**	1.01 (0.98-1.04)	0.6522	1.03 (0.99-1.06)	0.1657
**DPA1*01:03**	1.04 (1.01-1.07)	0.0117	0.97 (0.89-1.06)	0.4914
**DPA1*02:01**	0.96 (0.93-0.99)	0.0053	1.01 (0.98-1.05)	0.5033
**DPB1*01:01**	0.98 (0.93-1.02)	0.2882	1.01 (0.96-1.06)	0.8225
**DPB1*02:01**	0.99 (0.96-1.02)	0.5502	1.00 (0.96-1.04)	0.8794
**DPB1*03:01**	1.02 (0.98-1.06)	0.2590	1.00 (0.96-1.04)	0.8782
**DPB1*04:01**	1.02 (0.99-1.04)	0.0793	0.99 (0.95-1.02)	0.4802
**DPB1*04:02**	0.96 (0.92-0.99)	0.0137	0.96 (0.92-0.99)	0.0467
**DQA1*01:01**	1.00 (0.97-1.03)	0.9033	1.00 (0.96-1.03)	0.8010
**DQA1*01:02**	0.98 (0.95-1.01)	0.1759	1.01 (0.97-1.04)	0.6214
**DQA1*01:03**	0.98 (0.93-1.03)	0.3549	1.01 (0.96-1.06)	0.7519
**DQA1*02:01**	1.00 (0.97-1.03)	0.8319	1.01 (0.97-1.05)	0.6356
**DQA1*03:01**	1.01 (0.99-1.04)	0.3415	0.97 (0.94-1.01)	0.1210
**DQA1*05:01**	1.02 (0.99-1.05)	0.1007	1.00 (0.96-1.03)	0.8535
**DQB1*02:01**	1.02 (0.99-1.05)	0.1633	1.02 (0.99-1.06)	0.2388
**DQB1*02:02**	0.98 (0.94-1.01)	0.1908	1.02 (0.98-1.06)	0.3434
**DQB1*03:01**	1.01 (0.99-1.04)	0.3124	0.97 (0.93-1.00)	0.0667
**DQB1*03:02**	1.01 (0.97-1.04)	0.7558	0.97 (0.93-1.01)	0.1063
**DQB1*03:03**	1.04 (0.99-1.09)	0.1398	1.00 (0.94-1.05)	0.8840
**DQB1*05:01**	0.98 (0.95-1.02)	0.3497	0.99 (0.96-1.03)	0.7972
**DQB1*06:02**	0.99 (0.96-1.02)	0.6368	1.00 (0.96-1.04)	0.9914
**DQB1*06:03**	0.98 (0.93-1.03)	0.3366	1.00 (0.94-1.05)	0.9295
**DRB1*01:01**	0.98 (0.94-1.02)	0.3226	1.00 (0.96-1.04)	0.9030
**DRB1*03:01**	1.02 (0.98-1.05)	0.3269	1.02 (0.98-1.06)	0.2508
**DRB1*04:01**	1.01 (0.98-1.05)	0.5040	0.98 (0.94-1.02)	0.3174
**DRB1*07:01**	0.99 (0.96-1.02)	0.5911	1.01 (0.97-1.05)	0.5397
**DRB1*13:01**	0.98 (0.93-1.03)	0.3529	1.01 (0.95-1.06)	0.7802
**DRB1*15:01**	0.99 (0.96-1.03)	0.7175	1.00 (0.97-1.04)	0.9036

HLA alleles	Men		Women	
	Adjusted OR (95%CI) [a]	*P* value [b]	Adjusted OR (95%CI) [a]	*P* value [b]
**DRB3*01:01**	1.01 (0.98-1.04)	0.4844	1.02 (0.99-1.06)	0.1944
**DRB3*02:02**	1.01 (0.98-1.05)	0.3992	0.97 (0.93-1.01)	0.1074
**DRB4*01:01**	0.97 (0.93-1.01)	0.1527	1.01 (0.96-1.05)	0.7393
**DRB4*01:03**	1.02 (0.99-1.05)	0.0687	0.98 (0.95-1.02)	0.2845
**DRB5*01:01**	0.99 (0.96-1.03)	0.7195	1.00 (0.96-1.04)	0.9580

Abbreviations: CI, confidence interval; HLA, human leukocyte antigen; OR, odds ratio.

[a] Adjusted ORs and 95%CIs were computed from the multivariable-adjusted logistic regression model adjusted for age at recruitment, assessment center, and population structure (top 5 principal components of the genotypes) by using dosage of each HLA allele as a continuous variable.

[b] $P<0.001$ (0.05/50) was considered statistical significance in this analysis after Bonferroni correction.

Supplementary Table S7. Multivariable-adjusted associations between each HLA allele and incident risk of urinary and male reproductive system cancers

HLA alleles	Prostate cancer		Kidney cancer				Bladder cancer	
	Men		Men		Women		Men	
	Adjusted OR (95%CI) [a]	*P* value	Adjusted OR (95%CI) [a]	*P* value	Adjusted OR (95%CI) [a]	*P* value	Adjusted OR (95%CI) [a]	*P* value
**A*01:01**	0.94 (0.90-0.98)	0.0064	0.91 (0.79-1.06)	0.2282	0.91 (0.74-1.11)	0.3424	1.07 (0.93-1.24)	0.3208
**A*02:01**	1.05 (1.01-1.09)	0.0094	1.00 (0.88-1.13)	0.9980	0.97 (0.81-1.15)	0.6878	1.00 (0.88-1.13)	0.9768
**A*03:01**	0.97 (0.93-1.02)	0.2488	1.05 (0.90-1.23)	0.5437	1.15 (0.94-1.42)	0.1819	0.99 (0.84-1.16)	0.8996
**A*11:01**	1.01 (0.94-1.08)	0.7454	1.40 (1.14-1.71)	0.0013	1.01 (0.73-1.39)	0.9607	1.06 (0.84-1.34)	0.6279
**A*24:02**	1.01 (0.95-1.07)	0.7801	0.91 (0.73-1.14)	0.4128	1.60 (1.25-2.03)	**0.0002**[*]	0.91 (0.72-1.14)	0.3994
**B*07:02**	1.01 (0.97-1.06)	0.6612	1.09 (0.94-1.27)	0.2525	1.08 (0.87-1.33)	0.4801	1.07 (0.91-1.25)	0.4193
**B*08:01**	0.92 (0.88-0.97)	0.0012	1.00 (0.85-1.17)	0.9986	0.75 (0.59-0.95)	0.0196	1.00 (0.85-1.17)	0.9657
**B*15:01**	1.10 (1.03-1.17)	0.0041	0.93 (0.73-1.18)	0.5426	0.76 (0.53-1.09)	0.1316	1.18 (0.95-1.46)	0.1298
**B*40:01**	1.08 (1.01-1.16)	0.0259	0.92 (0.72-1.19)	0.5280	1.25 (0.92-1.69)	0.1489	0.96 (0.75-1.23)	0.7562
**B*44:02**	1.00 (0.95-1.06)	0.9256	1.00 (0.84-1.19)	0.9776	0.84 (0.65-1.09)	0.1882	0.95 (0.79-1.14)	0.5550
**B*44:03**	1.00 (0.94-1.07)	0.9281	0.77 (0.59-1.01)	0.0555	0.81 (0.57-1.16)	0.2442	0.89 (0.69-1.14)	0.3537
**C*03:03**	1.05 (0.98-1.13)	0.1592	1.06 (0.84-1.34)	0.6266	0.77 (0.53-1.13)	0.1787	1.19 (0.95-1.50)	0.1255
**C*03:04**	1.11 (1.05-1.18)	**0.0003**[*]	1.02 (0.84-1.26)	0.8124	1.20 (0.92-1.56)	0.1692	1.01 (0.82-1.24)	0.9331
**C*04:01**	0.90 (0.85-0.96)	**0.0009**[*]	1.03 (0.84-1.25)	0.8040	1.05 (0.80-1.38)	0.7227	0.83 (0.67-1.04)	0.1091
**C*05:01**	0.98 (0.93-1.03)	0.3653	0.92 (0.77-1.11)	0.3864	0.92 (0.72-1.18)	0.5155	0.93 (0.77-1.11)	0.4225
**C*06:02**	1.03 (0.97-1.08)	0.3843	0.79 (0.64-0.98)	0.0298	1.31 (1.03-1.66)	0.0278	1.27 (1.06-1.52)	0.0102
**C*07:01**	0.94 (0.90-0.98)	0.0053	0.99 (0.85-1.15)	0.8788	0.77 (0.62-0.96)	0.0182	1.02 (0.88-1.18)	0.7903
**C*07:02**	1.02 (0.98-1.07)	0.3516	1.05 (0.90-1.22)	0.5171	1.13 (0.92-1.38)	0.2502	1.05 (0.90-1.22)	0.5535
**DPA1*01:03**	1.04 (0.99-1.08)	0.0676	1.07 (0.93-1.24)	0.3405	1.31 (1.05-1.62)	0.0152	0.91 (0.79-1.05)	0.2159
**DPA1*02:01**	0.95 (0.91-1.00)	0.0455	0.86 (0.73-1.02)	0.0782	0.72 (0.57-0.93)	0.0098	1.06 (0.91-1.24)	0.4298
**DPB1*01:01**	0.97 (0.90-1.04)	0.3479	0.88 (0.69-1.13)	0.3131	0.63 (0.43-0.95)	0.0253	1.17 (0.93-1.46)	0.1734
**DPB1*02:01**	0.97 (0.92-1.03)	0.3087	1.02 (0.86-1.22)	0.7992	0.87 (0.67-1.12)	0.2851	0.92 (0.76-1.11)	0.3885
**DPB1*03:01**	1.01 (0.95-1.06)	0.8184	1.14 (0.96-1.36)	0.1384	1.00 (0.77-1.28)	0.9721	1.06 (0.88-1.28)	0.5132
**DPB1*04:01**	1.02 (0.98-1.05)	0.3175	0.99 (0.89-1.11)	0.9061	1.30 (1.11-1.51)	**0.0009**[*]	0.98 (0.87-1.10)	0.7135
**DPB1*04:02**	0.95 (0.90-0.99)	0.0672	1.06 (0.89-1.26)	0.5339	0.92 (0.71-1.19)	0.5249	0.96 (0.80-1.16)	0.6888
**DQA1*01:01**	1.00 (0.96-1.05)	0.8619	1.05 (0.90-1.23)	0.5613	1.17 (0.95-1.44)	0.1396	1.05 (0.90-1.23)	0.5494
**DQA1*01:02**	0.99 (0.95-1.03)	0.6256	1.12 (0.98-1.29)	0.0982	1.17 (0.97-1.40)	0.1058	0.92 (0.79-1.07)	0.2638
**DQA1*01:03**	1.02 (0.95-1.10)	0.5416	1.07 (0.85-1.36)	0.5518	0.91 (0.64-1.29)	0.6073	0.97 (0.75-1.24)	0.7838

HLA alleles	Prostate cancer		Kidney cancer				Bladder cancer	
	Men		Men		Women		Men	
	Adjusted OR (95%CI) [a]	*P* value	Adjusted OR (95%CI) [a]	*P* value	Adjusted OR (95%CI) [a]	*P* value	Adjusted OR (95%CI) [a]	*P* value
**DQA1*02:01**	0.99 (0.95-1.04)	0.7254	0.86 (0.73-1.02)	0.0790	1.02 (0.82-1.26)	0.8576	1.13 (0.97-1.32)	0.1219
**DQA1*03:01**	1.06 (1.02-1.10)	0.0055	1.00 (0.87-1.15)	0.9596	1.07 (0.89-1.29)	0.4783	1.00 (0.86-1.15)	0.9471
**DQA1*05:01**	0.95 (0.91-0.99)	0.0091	1.01 (0.88-1.15)	0.9258	0.72 (0.59-0.88)	0.0013	0.95 (0.83-1.09)	0.4764
**DQB1*02:01**	0.92 (0.88-0.96)	**0.0004***	0.99 (0.85-1.16)	0.9301	0.78 (0.62-0.99)	0.0411	1.05 (0.89-1.22)	0.5785
**DQB1*02:02**	0.96 (0.91-1.01)	0.1112	0.87 (0.72-1.06)	0.1578	0.94 (0.72-1.21)	0.6103	1.08 (0.90-1.29)	0.3969
**DQB1*03:01**	1.02 (0.98-1.07)	0.3212	1.01 (0.88-1.17)	0.8542	0.90 (0.73-1.10)	0.3029	0.88 (0.76-1.03)	0.1160
**DQB1*03:02**	1.07 (1.02-1.13)	0.0079	0.98 (0.82-1.18)	0.8378	1.09 (0.85-1.39)	0.4859	1.10 (0.92-1.32)	0.2798
**DQB1*03:03**	1.07 (1.00-1.15)	0.0459	0.89 (0.68-1.16)	0.3883	1.05 (0.75-1.48)	0.7586	1.07 (0.84-1.37)	0.5752
**DQB1*05:01**	0.99 (0.94-1.04)	0.7065	1.01 (0.85-1.20)	0.9142	1.19 (0.96-1.48)	0.1147	0.97 (0.81-1.16)	0.7400
**DQB1*06:02**	1.00 (0.95-1.04)	0.8773	1.12 (0.97-1.31)	0.1303	1.31 (1.07-1.60)	0.0080	0.92 (0.78-1.09)	0.3350
**DQB1*06:03**	1.02 (0.95-1.09)	0.6369	1.11 (0.87-1.41)	0.4119	0.88 (0.61-1.26)	0.4817	0.98 (0.76-1.27)	0.8991
**DRB1*01:01**	0.99 (0.94-1.05)	0.7365	0.98 (0.81-1.19)	0.8738	1.34 (1.06-1.69)	0.0140	1.01 (0.83-1.22)	0.9507
**DRB1*03:01**	0.91 (0.87-0.95)	**0.0001***	0.97 (0.83-1.14)	0.7509	0.79 (0.63-1.00)	0.0492	1.03 (0.88-1.21)	0.6930
**DRB1*04:01**	1.03 (0.98-1.09)	0.1937	1.08 (0.91-1.28)	0.3637	1.12 (0.89-1.41)	0.3289	0.96 (0.80-1.14)	0.6273
**DRB1*07:01**	0.99 (0.94-1.03)	0.6022	0.86 (0.72-1.01)	0.0688	1.01 (0.81-1.25)	0.9324	1.13 (0.96-1.31)	0.1327
**DRB1*13:01**	1.01 (0.94-1.09)	0.7934	1.07 (0.84-1.37)	0.5860	0.87 (0.60-1.26)	0.4542	0.96 (0.74-1.25)	0.7717
**DRB1*15:01**	1.00 (0.96-1.05)	0.9592	1.12 (0.96-1.30)	0.1503	1.30 (1.06-1.59)	0.0100	0.94 (0.80-1.10)	0.4383
**DRB3*01:01**	0.94 (0.90-0.98)	0.0039	1.03 (0.88-1.19)	0.7295	0.73 (0.58-0.91)	0.0065	1.02 (0.87-1.18)	0.8256
**DRB3*02:02**	1.00 (0.96-1.05)	0.9227	1.07 (0.91-1.26)	0.4071	0.81 (0.63-1.03)	0.0906	0.99 (0.84-1.17)	0.9003
**DRB4*01:01**	0.97 (0.91-1.03)	0.2985	0.86 (0.69-1.07)	0.1721	0.91 (0.68-1.22)	0.5386	1.01 (0.83-1.24)	0.8966
**DRB4*01:03**	1.05 (1.02-1.09)	0.0051	0.97 (0.86-1.11)	0.6889	1.07 (0.90-1.28)	0.4168	1.04 (0.91-1.18)	0.5648
**DRB5*01:01**	1.00 (0.96-1.05)	0.9768	1.11 (0.95-1.29)	0.1908	1.31 (1.07-1.60)	0.0088	0.92 (0.78-1.09)	0.3344

Abbreviations: CI, confidence interval; HLA, human leukocyte antigen; OR, odds ratio.

[a] Adjusted ORs and 95%CIs were computed from the multivariable-adjusted logistic model adjusted for age at recruitment, assessment center, and population structure (top 5 principal components of the genotypes) by using dosage of each HLA allele as a continuous variable.

*P<0.001 (0.05/50) was considered statistically significance in this analysis after Bonferroni correction, which were highlighted in bold.

Supplementary Table S8. Multivariable-adjusted associations between each HLA allele and incident risk of breast cancer

HLA alleles	Breast cancer	
	Adjusted OR (95%CI) [a]	*P* value [b]
**A*01:01**	1.01 (0.97-1.06)	0.6171
**A*02:01**	1.01 (0.97-1.05)	0.7303
**A*03:01**	0.95 (0.91-0.99)	0.0732
**A*11:01**	1.00 (0.93-1.08)	0.9708
**A*24:02**	0.98 (0.92-1.06)	0.6541
**B*07:02**	0.98 (0.93-1.03)	0.4058
**B*08:01**	1.02 (0.96-1.07)	0.5572
**B*15:01**	0.99 (0.92-1.07)	0.8472
**B*40:01**	0.97 (0.90-1.05)	0.5103
**B*44:02**	0.98 (0.92-1.03)	0.3921
**B*44:03**	1.05 (0.97-1.13)	0.2321
**C*03:03**	0.95 (0.88-1.03)	0.2332
**C*03:04**	1.00 (0.94-1.07)	0.9283
**C*04:01**	0.95 (0.89-1.01)	0.1211
**C*05:01**	1.00 (0.95-1.06)	0.9199
**C*06:02**	1.07 (1.00-1.14)	0.0345
**C*07:01**	1.01 (0.96-1.06)	0.6643
**C*07:02**	0.98 (0.93-1.03)	0.3973
**DPA1*01:03**	0.97 (0.92-1.01)	0.1458
**DPA1*02:01**	1.06 (1.00-1.11)	0.0317
**DPB1*01:01**	1.04 (0.96-1.12)	0.3671
**DPB1*02:01**	0.98 (0.93-1.04)	0.5794
**DPB1*03:01**	1.00 (0.94-1.06)	0.9729
**DPB1*04:01**	0.99 (0.95-1.03)	0.5633
**DPB1*04:02**	1.01 (0.95-1.07)	0.7894
**DQA1*01:01**	0.97 (0.92-1.02)	0.2004
**DQA1*01:02**	1.00 (0.96-1.05)	0.9311
**DQA1*01:03**	1.02 (0.95-1.11)	0.5513
**DQA1*02:01**	1.04 (0.99-1.10)	0.0966
**DQA1*03:01**	0.97 (0.93-1.02)	0.2084
**DQA1*05:01**	1.01 (0.96-1.05)	0.7723
**DQB1*02:01**	1.02 (0.97-1.07)	0.4137
**DQB1*02:02**	1.05 (0.99-1.11)	0.1081
**DQB1*03:01**	1.00 (0.95-1.04)	0.8527
**DQB1*03:02**	0.95 (0.90-1.01)	0.1142
**DQB1*03:03**	0.99 (0.91-1.07)	0.8097
**DQB1*05:01**	0.97 (0.92-1.02)	0.2683
**DQB1*06:02**	0.97 (0.92-1.02)	0.1876
**DQB1*06:03**	1.01 (0.93-1.09)	0.8630
**DRB1*01:01**	0.98 (0.92-1.04)	0.5611
**DRB1*03:01**	1.02 (0.97-1.07)	0.4956
**DRB1*04:01**	1.01 (0.95-1.06)	0.8569
**DRB1*07:01**	1.04 (0.99-1.10)	0.1125
**DRB1*13:01**	1.02 (0.94-1.11)	0.5621
**DRB1*15:01**	0.97 (0.92-1.02)	0.2650

HLA alleles	Breast cancer	
	**Adjusted OR (95%CI) [a]**	***P* value [b]**
**DRB3*01:01**	1.03 (0.98-1.08)	0.2201
**DRB3*02:02**	0.97 (0.92-1.03)	0.3314
**DRB4*01:01**	1.04 (0.97-1.11)	0.2688
**DRB4*01:03**	0.99 (0.95-1.03)	0.5245
**DRB5*01:01**	0.97 (0.92-1.02)	0.2239

Abbreviations: CI, confidence interval; HLA, human leukocyte antigen; OR, odds ratio.

[a] Adjusted ORs and 95%CIs were computed from the multivariable-adjusted logistic regression model adjusted for age at recruitment, assessment center, and population structure (top 5 principal components of the genotypes) by using dosage of each HLA allele as a continuous variable.

[b] $P<0.001$ (0.05/50) was considered statistically significance in this analysis after Bonferroni correction.

Supplementary Table S9. Multivariable-adjusted associations between each HLA allele and incident risk of lung cancer

HLA alleles	Lung cancer			
	Men		Women	
	Adjusted OR (95%CI) [a]	*P* value	Adjusted OR (95%CI) [a]	*P* value
**A*01:01**	1.16 (1.06-1.27)	0.0015	1.09 (0.99-1.20)	0.0939
**A*02:01**	0.89 (0.82-0.98)	0.0116	1.00 (0.91-1.09)	0.9843
**A*03:01**	1.10 (0.99-1.22)	0.0800	1.05 (0.94-1.18)	0.3803
**A*11:01**	0.90 (0.76-1.06)	0.2208	0.94 (0.79-1.13)	0.5243
**A*24:02**	0.99 (0.86-1.15)	0.9024	0.98 (0.84-1.14)	0.7823
**B*07:02**	1.00 (0.90-1.11)	0.9434	0.96 (0.86-1.08)	0.5204
**B*08:01**	1.19 (1.08-1.32)	**0.0006***	1.11 (0.99-1.24)	0.0658
**B*15:01**	1.00 (0.85-1.17)	0.9916	1.00 (0.85-1.19)	0.9571
**B*40:01**	0.85 (0.71-1.01)	0.0696	1.00 (0.84-1.20)	0.9836
**B*44:02**	0.96 (0.85-1.08)	0.4919	1.02 (0.89-1.16)	0.8011
**B*44:03**	0.82 (0.69-0.98)	0.0286	0.93 (0.78-1.11)	0.4380
**C*03:03**	0.96 (0.81-1.14)	0.6501	1.01 (0.84-1.20)	0.9536
**C*03:04**	0.89 (0.77-1.03)	0.1324	1.03 (0.89-1.20)	0.6626
**C*04:01**	0.89 (0.77-1.02)	0.1027	0.97 (0.84-1.13)	0.6996
**C*05:01**	0.99 (0.88-1.11)	0.8577	1.05 (0.93-1.20)	0.4071
**C*06:02**	1.17 (1.04-1.32)	0.0122	1.10 (0.96-1.26)	0.1597
**C*07:01**	1.15 (1.05-1.27)	0.0028	1.08 (0.97-1.19)	0.1694
**C*07:02**	0.98 (0.88-1.09)	0.7150	0.98 (0.88-1.10)	0.7205
**DPA1*01:03**	1.03 (0.94-1.14)	0.4936	0.96 (0.87-1.07)	0.4866
**DPA1*02:01**	0.97 (0.87-1.08)	0.5550	0.95 (0.84-1.07)	0.3859
**DPB1*01:01**	1.05 (0.90-1.22)	0.5597	0.96 (0.81-1.15)	0.6624
**DPB1*02:01**	0.95 (0.84-1.07)	0.4008	0.93 (0.81-1.06)	0.2534
**DPB1*03:01**	0.94 (0.83-1.07)	0.3786	1.05 (0.92-1.20)	0.4518
**DPB1*04:01**	1.06 (0.98-1.14)	0.1443	1.05 (0.96-1.13)	0.2808
**DPB1*04:02**	1.01 (0.89-1.14)	0.9270	0.92 (0.80-1.05)	0.2028
**DQA1*01:01**	0.87 (0.78-0.97)	0.0156	0.93 (0.83-1.05)	0.2483
**DQA1*01:02**	0.95 (0.86-1.05)	0.3121	1.00 (0.90-1.11)	0.9659
**DQA1*01:03**	0.96 (0.81-1.14)	0.6687	0.96 (0.80-1.15)	0.6526
**DQA1*02:01**	0.98 (0.88-1.09)	0.7229	0.97 (0.87-1.09)	0.6448
**DQA1*03:01**	0.97 (0.88-1.06)	0.4925	1.02 (0.92-1.13)	0.7360
**DQA1*05:01**	1.22 (1.12-1.33)	**<0.0001***	1.06 (0.97-1.17)	0.2154
**DQB1*02:01**	1.30 (1.18-1.44)	**<0.0001***	1.12 (1.00-1.25)	0.0485
**DQB1*02:02**	0.97 (0.86-1.10)	0.6460	0.97 (0.85-1.11)	0.6377
**DQB1*03:01**	1.05 (0.95-1.16)	0.3061	0.99 (0.89-1.10)	0.8299
**DQB1*03:02**	0.84 (0.73-0.95)	0.0080	1.01 (0.88-1.15)	0.9409
**DQB1*03:03**	1.04 (0.88-1.23)	0.6313	1.00 (0.84-1.21)	0.9589
**DQB1*05:01**	0.82 (0.72-0.93)	0.0018	0.91 (0.80-1.03)	0.1442
**DQB1*06:02**	1.01 (0.91-1.12)	0.8775	0.99 (0.89-1.12)	0.9292
**DQB1*06:03**	0.95 (0.80-1.13)	0.5698	0.92 (0.76-1.12)	0.4214
**DRB1*01:01**	0.79 (0.68-0.91)	**0.0009***	0.92 (0.80-1.06)	0.2510
**DRB1*03:01**	1.30 (1.18-1.43)	**<0.0001***	1.12 (1.01-1.25)	0.0380
**DRB1*04:01**	1.01 (0.90-1.13)	0.8989	1.03 (0.91-1.17)	0.6342
**DRB1*07:01**	0.97 (0.87-1.08)	0.6179	0.98 (0.87-1.10)	0.6802
**DRB1*13:01**	0.99 (0.83-1.18)	0.9055	0.94 (0.77-1.13)	0.5004

HLA alleles	Lung cancer			
	Men		Women	
	Adjusted OR (95%CI) [a]	*P* value	Adjusted OR (95%CI) [a]	*P* value
**DRB1*15:01**	1.00 (0.90-1.11)	0.9300	0.99 (0.88-1.11)	0.8883
**DRB3*01:01**	1.23 (1.12-1.35)	**<0.0001** *	1.10 (0.99-1.22)	0.0842
**DRB3*02:02**	1.06 (0.95-1.18)	0.3030	0.95 (0.84-1.07)	0.4173
**DRB4*01:01**	0.95 (0.82-1.09)	0.4259	0.89 (0.77-1.04)	0.1456
**DRB4*01:03**	0.98 (0.90-1.07)	0.6809	1.04 (0.94-1.14)	0.4563
**DRB5*01:01**	1.00 (0.90-1.11)	0.9916	1.00 (0.89-1.12)	0.9752

Abbreviations: CI, confidence interval; HLA, human leukocyte antigen; OR, odds ratio.

[a] Adjusted ORs and 95%CIs were computed from the multivariable-adjusted logistic regression model adjusted for age at recruitment, assessment center, and population structure (top 5 principal components of the genotypes) by using dosage of each HLA allele as a continuous variable.

* P<0.001 (0.05/50) was considered statistically significance in this analysis after Bonferroni correction, which were highlighted in bold.

Supplementary Table S10. Multivariable-adjusted associations between each HLA allele and incident risk of digestive cancers

HLA alleles	Colorectal cancer				Esophageal cancer		Gastric cancer	
	Men		Women		Men		Men	
	Adjusted OR (95%CI) [a]	*P* value [b]	Adjusted OR (95%CI) [a]	*P* value [b]	Adjusted OR (95%CI) [a]	*P* value [b]	Adjusted OR (95%CI) [a]	*P* value [b]
**A*01:01**	1.02 (0.95-1.10)	0.5503	1.07 (0.98-1.16)	0.1270	0.95 (0.81-1.11)	0.5095	1.02 (0.84-1.23)	0.8635
**A*02:01**	1.00 (0.94-1.07)	0.9232	1.05 (0.98-1.14)	0.1693	1.03 (0.90-1.18)	0.6279	1.11 (0.94-1.31)	0.2174
**A*03:01**	0.95 (0.87-1.03)	0.2047	0.99 (0.90-1.09)	0.8310	1.03 (0.87-1.22)	0.7301	0.87 (0.69-1.09)	0.2173
**A*11:01**	1.10 (0.98-1.24)	0.1135	0.86 (0.73-0.99)	0.0510	1.16 (0.92-1.47)	0.2109	0.79 (0.56-1.13)	0.2001
**A*24:02**	1.11 (1.00-1.24)	0.0462	0.86 (0.75-0.99)	0.0329	0.85 (0.66-1.09)	0.1939	0.79 (0.57-1.09)	0.1537
**B*07:02**	1.04 (0.96-1.12)	0.3700	0.97 (0.88-1.07)	0.5448	1.01 (0.85-1.19)	0.9163	0.91 (0.73-1.13)	0.3975
**B*08:01**	1.02 (0.94-1.11)	0.6457	1.12 (1.02-1.23)	0.0212	1.06 (0.90-1.25)	0.5006	1.13 (0.92-1.39)	0.2468
**B*15:01**	0.99 (0.87-1.11)	0.8189	1.07 (0.94-1.23)	0.3056	0.76 (0.57-1.01)	0.0543	0.81 (0.57-1.15)	0.2395
**B*40:01**	0.99 (0.87-1.13)	0.9177	0.96 (0.82-1.12)	0.5862	1.02 (0.78-1.32)	0.8887	1.07 (0.78-1.48)	0.6702
**B*44:02**	1.05 (0.96-1.15)	0.2794	1.05 (0.95-1.17)	0.3350	0.95 (0.79-1.16)	0.6205	1.09 (0.87-1.38)	0.4474
**B*44:03**	0.87 (0.77-1.00)	0.0437	0.93 (0.80-1.08)	0.3577	1.21 (0.96-1.53)	0.1056	1.30 (0.97-1.73)	0.0753
**C*03:03**	1.01 (0.89-1.14)	0.8859	1.02 (0.88-1.18)	0.8329	0.93 (0.71-1.21)	0.5795	0.80 (0.55-1.15)	0.2325
**C*03:04**	0.97 (0.87-1.08)	0.5781	0.98 (0.86-1.11)	0.7196	0.91 (0.73-1.15)	0.4506	0.95 (0.72-1.27)	0.7523
**C*04:01**	1.02 (0.92-1.13)	0.6976	1.00 (0.89-1.14)	0.9598	1.12 (0.91-1.38)	0.2832	1.14 (0.88-1.49)	0.3263
**C*05:01**	1.04 (0.95-1.14)	0.3507	1.08 (0.97-1.20)	0.1547	0.96 (0.79-1.16)	0.6838	1.05 (0.83-1.33)	0.6749
**C*06:02**	1.06 (0.96-1.17)	0.2217	1.03 (0.91-1.15)	0.6816	0.80 (0.63-0.99)	0.0533	1.00 (0.77-1.31)	0.9819
**C*07:01**	1.00 (0.93-1.08)	0.9136	1.09 (0.99-1.19)	0.0509	1.02 (0.88-1.20)	0.7693	1.11 (0.92-1.35)	0.2764
**C*07:02**	1.03 (0.96-1.12)	0.3922	0.93 (0.84-1.02)	0.1391	0.98 (0.83-1.16)	0.8527	0.90 (0.72-1.11)	0.3261
**DPA1*01:03**	1.06 (0.98-1.14)	0.1575	1.02 (0.94-1.12)	0.6066	0.94 (0.81-1.09)	0.4249	1.01 (0.83-1.23)	0.8978
**DPA1*02:01**	0.95 (0.87-1.03)	0.2041	0.97 (0.88-1.07)	0.5699	1.02 (0.86-1.21)	0.8147	0.97 (0.78-1.21)	0.8143
**DPB1*01:01**	1.02 (0.90-1.15)	0.7561	1.00 (0.87-1.16)	0.9737	1.16 (0.91-1.47)	0.2259	0.99 (0.71-1.36)	0.9357
**DPB1*02:01**	0.95 (0.87-1.05)	0.3084	1.08 (0.97-1.20)	0.1764	1.02 (0.84-1.23)	0.8341	1.01 (0.80-1.29)	0.9120
**DPB1*03:01**	0.96 (0.87-1.06)	0.4696	0.92 (0.82-1.04)	0.1697	0.93 (0.76-1.14)	0.4790	1.10 (0.86-1.40)	0.4618
**DPB1*04:01**	1.05 (0.99-1.11)	0.1332	1.04 (0.97-1.12)	0.2411	0.99 (0.87-1.11)	0.8187	0.96 (0.83-1.12)	0.6391
**DPB1*04:02**	1.01 (0.92-1.11)	0.8940	0.92 (0.82-1.03)	0.1475	0.98 (0.80-1.19)	0.8119	1.12 (0.88-1.41)	0.3631
**DQA1*01:01**	0.96 (0.88-1.04)	0.3304	1.06 (0.96-1.16)	0.2723	1.10 (0.93-1.30)	0.2721	0.96 (0.76-1.19)	0.6885
**DQA1*01:02**	1.02 (0.95-1.10)	0.6001	0.94 (0.86-1.03)	0.1678	1.06 (0.92-1.23)	0.4171	1.07 (0.89-1.29)	0.4718

HLA alleles	Colorectal cancer				Esophageal cancer		Gastric cancer	
	Men		Women		Men		Men	
	Adjusted OR (95%CI) [a]	*P* value [b]	Adjusted OR (95%CI) [a]	*P* value [b]	Adjusted OR (95%CI) [a]	*P* value [b]	Adjusted OR (95%CI) [a]	*P* value [b]
**DQA1*01:03**	0.92 (0.81-1.05)	0.2380	1.10 (0.96-1.28)	0.1780	0.87 (0.66-1.15)	0.3337	0.74 (0.50-1.08)	0.1221
**DQA1*02:01**	0.97 (0.89-1.05)	0.4279	1.02 (0.92-1.12)	0.7524	1.03 (0.87-1.22)	0.7451	1.11 (0.90-1.37)	0.3246
**DQA1*03:01**	1.04 (0.96-1.11)	0.3289	0.98 (0.90-1.07)	0.7278	0.83 (0.70-0.97)	0.0179	0.97 (0.80-1.17)	0.7181
**DQA1*05:01**	1.04 (0.97-1.11)	0.3113	1.04 (0.95-1.12)	0.4024	1.09 (0.95-1.25)	0.2408	1.01 (0.85-1.21)	0.8801
**DQB1*02:01**	1.03 (0.95-1.11)	0.5131	1.09 (0.99-1.20)	0.0735	1.05 (0.89-1.24)	0.5493	0.98 (0.79-1.21)	0.8324
**DQB1*02:02**	0.96 (0.87-1.06)	0.4362	1.03 (0.92-1.15)	0.6188	1.13 (0.94-1.37)	0.1911	1.03 (0.81-1.32)	0.7876
**DQB1*03:01**	1.03 (0.95-1.11)	0.4430	0.91 (0.83-1.00)	0.0467	0.91 (0.77-1.07)	0.2452	1.11 (0.92-1.35)	0.2743
**DQB1*03:02**	1.01 (0.92-1.11)	0.8171	1.02 (0.91-1.14)	0.7459	0.90 (0.73-1.10)	0.3083	0.84 (0.65-1.10)	0.2128
**DQB1*03:03**	0.99 (0.87-1.13)	0.9092	1.03 (0.89-1.20)	0.6846	0.77 (0.57-1.04)	0.0885	1.12 (0.81-1.55)	0.5076
**DQB1*05:01**	0.95 (0.87-1.04)	0.2880	1.06 (0.95-1.17)	0.2806	1.09 (0.91-1.30)	0.3596	1.01 (0.80-1.28)	0.9425
**DQB1*06:02**	1.00 (0.92-1.09)	0.9929	0.94 (0.85-1.04)	0.2076	1.02 (0.86-1.21)	0.8188	1.00 (0.81-1.24)	0.9971
**DQB1*06:03**	0.96 (0.84-1.09)	0.5176	1.17 (1.01-1.35)	0.0373	0.82 (0.61-1.11)	0.2055	0.73 (0.49-1.09)	0.1199
**DRB1*01:01**	0.96 (0.87-1.06)	0.4203	1.00 (0.89-1.12)	0.9583	1.14 (0.94-1.39)	0.1882	0.96 (0.74-1.26)	0.7939
**DRB1*03:01**	1.03 (0.95-1.12)	0.4630	1.09 (0.99-1.20)	0.0595	1.07 (0.90-1.26)	0.4463	0.98 (0.79-1.22)	0.8570
**DRB1*04:01**	1.05 (0.96-1.14)	0.3254	0.98 (0.88-1.09)	0.7434	0.82 (0.67-0.99)	0.0514	1.03 (0.81-1.30)	0.8334
**DRB1*07:01**	0.96 (0.88-1.04)	0.3395	1.02 (0.93-1.12)	0.6753	1.01 (0.86-1.20)	0.8665	1.11 (0.90-1.36)	0.3405
**DRB1*13:01**	0.94 (0.82-1.08)	0.3893	1.13 (0.98-1.32)	0.0962	0.83 (0.62-1.12)	0.2274	0.74 (0.50-1.10)	0.1388
**DRB1*15:01**	1.02 (0.94-1.10)	0.6979	0.95 (0.86-1.05)	0.2740	1.02 (0.87-1.21)	0.7883	1.00 (0.80-1.24)	0.9829
**DRB3*01:01**	1.02 (0.95-1.10)	0.5690	1.13 (1.04-1.24)	0.0059	1.01 (0.86-1.18)	0.9446	1.02 (0.83-1.24)	0.8805
**DRB3*02:02**	1.00 (0.92-1.09)	0.9992	0.95 (0.85-1.05)	0.3050	1.06 (0.89-1.26)	0.5402	0.86 (0.67-1.09)	0.2025
**DRB4*01:01**	0.94 (0.85-1.05)	0.2900	1.06 (0.94-1.20)	0.3196	1.15 (0.94-1.41)	0.1788	1.10 (0.84-1.43)	0.4858
**DRB4*01:03**	1.03 (0.97-1.11)	0.3062	0.99 (0.92-1.08)	0.8934	0.80 (0.69-0.93)	0.0030	1.00 (0.84-1.19)	0.9786
**DRB5*01:01**	1.02 (0.94-1.10)	0.6629	0.95 (0.86-1.05)	0.2990	1.02 (0.86-1.20)	0.8613	1.01 (0.82-1.25)	0.9227

HLA alleles	Pancreatic cancer				Liver cancer	
	Men		Women		Men	
	Adjusted OR (95%CI) [a]	*P* value [b]	Adjusted OR (95%CI) [a]	*P* value [b]	Adjusted OR (95%CI) [a]	*P* value [b]
**A*01:01**	1.02 (0.86-1.20)	0.8518	1.09 (0.91-1.31)	0.3299	0.98 (0.79-1.21)	0.8520
**A*02:01**	0.87 (0.75-1.01)	0.0627	0.90 (0.76-1.06)	0.1965	1.04 (0.87-1.26)	0.6418
**A*03:01**	1.14 (0.96-1.36)	0.1398	1.06 (0.87-1.30)	0.5461	0.97 (0.76-1.23)	0.8052
**A*11:01**	1.04 (0.80-1.36)	0.7612	0.76 (0.54-1.07)	0.1119	0.97 (0.68-1.38)	0.8562
**A*24:02**	1.10 (0.86-1.39)	0.4512	1.09 (0.83-1.42)	0.5402	0.84 (0.60-1.19)	0.3301
**B*07:02**	0.91 (0.75-1.10)	0.3165	0.90 (0.72-1.11)	0.3058	0.89 (0.70-1.13)	0.3485
**B*08:01**	1.12 (0.94-1.34)	0.2193	1.21 (1.00-1.47)	0.0488	0.97 (0.76-1.23)	0.7904
**B*15:01**	1.13 (0.87-1.46)	0.3592	0.83 (0.60-1.16)	0.2779	1.23 (0.89-1.68)	0.2109
**B*40:01**	0.84 (0.62-1.15)	0.2788	1.07 (0.79-1.45)	0.6668	1.12 (0.79-1.59)	0.5110
**B*44:02**	0.99 (0.81-1.22)	0.9241	1.06 (0.85-1.33)	0.5954	1.00 (0.77-1.29)	0.9743
**B*44:03**	1.00 (0.76-1.32)	0.9930	0.99 (0.72-1.34)	0.9300	1.00 (0.71-1.43)	0.9789
**C*03:03**	1.08 (0.82-1.42)	0.5946	1.06 (0.78-1.44)	0.7163	1.38 (1.01-1.89)	0.0454
**C*03:04**	0.89 (0.69-1.15)	0.3804	0.96 (0.73-1.26)	0.7818	0.92 (0.67-1.27)	0.6136
**C*04:01**	1.17 (0.94-1.46)	0.1674	0.76 (0.56-1.02)	0.0652	0.99 (0.73-1.34)	0.9321
**C*05:01**	1.07 (0.88-1.31)	0.4830	1.05 (0.84-1.31)	0.6955	1.11 (0.87-1.43)	0.4035
**C*06:02**	1.00 (0.80-1.26)	0.9892	0.92 (0.71-1.19)	0.5258	1.09 (0.83-1.44)	0.5435
**C*07:01**	1.03 (0.87-1.22)	0.7238	1.22 (1.02-1.46)	0.0300	1.03 (0.83-1.27)	0.8102
**C*07:02**	0.97 (0.81-1.16)	0.7346	0.93 (0.76-1.13)	0.4560	0.97 (0.77-1.22)	0.7770
**DPA1*01:03**	1.09 (0.92-1.30)	0.3035	0.94 (0.78-1.12)	0.4758	0.98 (0.80-1.22)	0.8789
**DPA1*02:01**	0.89 (0.73-1.07)	0.2136	1.06 (0.87-1.30)	0.5619	1.02 (0.81-1.29)	0.8349
**DPB1*01:01**	0.66 (0.48-0.92)	0.0146	0.89 (0.65-1.23)	0.4815	0.99 (0.69-1.40)	0.9375
**DPB1*02:01**	0.87 (0.70-1.08)	0.2066	1.23 (0.99-1.52)	0.0584	1.20 (0.94-1.54)	0.1477
**DPB1*03:01**	1.17 (0.95-1.43)	0.1407	1.07 (0.85-1.35)	0.5501	1.03 (0.78-1.35)	0.8511
**DPB1*04:01**	1.11 (0.98-1.27)	0.1078	0.88 (0.76-1.02)	0.0854	0.89 (0.75-1.05)	0.1704
**DPB1*04:02**	0.88 (0.70-1.10)	0.2491	0.96 (0.76-1.22)	0.7437	0.93 (0.70-1.23)	0.6147
**DQA1*01:01**	1.02 (0.85-1.23)	0.8534	0.82 (0.65-1.02)	0.0767	0.75 (0.57-0.98)	0.0343
**DQA1*01:02**	0.93 (0.79-1.10)	0.4159	0.95 (0.79-1.15)	0.6138	0.89 (0.72-1.11)	0.3076
**DQA1*01:03**	0.98 (0.73-1.31)	0.8900	1.28 (0.96-1.70)	0.0873	1.09 (0.77-1.55)	0.6253
**DQA1*02:01**	0.94 (0.78-1.14)	0.5371	0.87 (0.70-1.08)	0.2105	1.33 (1.07-1.64)	0.0095
**DQA1*03:01**	1.04 (0.89-1.22)	0.6091	1.12 (0.94-1.33)	0.2169	1.05 (0.86-1.29)	0.6100

HLA alleles	Pancreatic cancer				Liver cancer	
	Men		Women		Men	
	Adjusted OR (95%CI) [a]	*P* value [b]	Adjusted OR (95%CI) [a]	*P* value [b]	Adjusted OR (95%CI) [a]	*P* value [b]
**DQA1*05:01**	1.09 (0.93-1.26)	0.2907	1.12 (0.95-1.32)	0.1872	0.97 (0.79-1.18)	0.7381
**DQB1*02:01**	1.09 (0.91-1.30)	0.3359	1.09 (0.89-1.33)	0.3934	1.05 (0.84-1.32)	0.6530
**DQB1*02:02**	0.88 (0.70-1.10)	0.2539	0.80 (0.62-1.04)	0.0924	1.22 (0.95-1.57)	0.1219
**DQB1*03:01**	1.05 (0.89-1.24)	0.5806	1.08 (0.90-1.30)	0.4124	1.02 (0.82-1.26)	0.8826
**DQB1*03:02**	0.99 (0.80-1.22)	0.9135	1.16 (0.93-1.46)	0.1861	0.91 (0.69-1.21)	0.5237
**DQB1*03:03**	1.13 (0.85-1.49)	0.3948	1.07 (0.78-1.46)	0.6872	1.37 (0.99-1.90)	0.0566
**DQB1*05:01**	0.92 (0.75-1.14)	0.4492	0.82 (0.65-1.05)	0.1128	0.68 (0.50-0.92)	0.0112
**DQB1*06:02**	0.93 (0.77-1.13)	0.4743	0.96 (0.78-1.19)	0.7259	0.81 (0.63-1.04)	0.0990
**DQB1*06:03**	0.96 (0.71-1.30)	0.7893	1.25 (0.93-1.68)	0.1421	1.10 (0.77-1.58)	0.5965
**DRB1*01:01**	0.91 (0.72-1.15)	0.4504	0.82 (0.62-1.07)	0.1380	0.72 (0.52-0.99)	0.0507
**DRB1*03:01**	1.06 (0.89-1.27)	0.5076	1.10 (0.90-1.34)	0.3431	1.04 (0.83-1.31)	0.7225
**DRB1*04:01**	1.05 (0.86-1.28)	0.6556	1.08 (0.87-1.35)	0.4679	1.12 (0.87-1.44)	0.3670
**DRB1*07:01**	0.95 (0.79-1.15)	0.6093	0.88 (0.71-1.09)	0.2570	1.36 (1.10-1.68)	0.0045
**DRB1*13:01**	1.00 (0.74-1.34)	0.9855	1.37 (1.03-1.82)	0.0329	1.08 (0.75-1.56)	0.6814
**DRB1*15:01**	0.90 (0.74-1.09)	0.2708	0.96 (0.78-1.19)	0.7198	0.83 (0.65-1.07)	0.1520
**DRB3*01:01**	1.07 (0.90-1.27)	0.4326	1.22 (1.02-1.46)	0.0309	0.93 (0.74-1.16)	0.5157
**DRB3*02:02**	1.10 (0.92-1.33)	0.2975	1.07 (0.87-1.32)	0.5400	1.15 (0.91-1.45)	0.2507
**DRB4*01:01**	0.99 (0.79-1.26)	0.9625	0.75 (0.55-0.99)	0.0509	1.25 (0.95-1.64)	0.1137
**DRB4*01:03**	1.03 (0.89-1.20)	0.6482	1.10 (0.93-1.29)	0.2638	1.19 (0.99-1.42)	0.0638
**DRB5*01:01**	0.90 (0.75-1.09)	0.3008	0.96 (0.78-1.19)	0.7274	0.84 (0.65-1.07)	0.1613

Abbreviations: CI, confidence interval; HLA, human leukocyte antigen; OR, odds ratio.

[a] Adjusted ORs and 95%CIs were computed from the multivariable-adjusted logistic regression model adjusted for age at recruitment, assessment center, and population structure (top 5 principal components of the genotypes) by using dosage of each HLA allele as a continuous variable.

[b] P<0.001 (0.05/50) was considered statistically significance in this analysis after Bonferroni correction.

Supplementary Table S11. Multivariable-adjusted associations between each HLA allele and incident risk of skin cancer

HLA alleles	Skin cancer			
	Men		Women	
	Adjusted OR (95%CI) [a]	*P* value [b]	Adjusted OR (95%CI) [a]	*P* value [b]
**A*01:01**	1.00 (0.90-1.11)	0.9876	0.84 (0.74-0.94)	0.0032
**A*02:01**	0.96 (0.87-1.06)	0.3901	0.96 (0.86-1.06)	0.3672
**A*03:01**	1.00 (0.89-1.13)	0.9886	1.03 (0.91-1.16)	0.6670
**A*11:01**	1.02 (0.85-1.22)	0.8468	1.11 (0.93-1.32)	0.2597
**A*24:02**	1.05 (0.89-1.24)	0.5424	0.95 (0.80-1.13)	0.5985
**B*07:02**	0.99 (0.88-1.12)	0.8908	1.12 (0.99-1.26)	0.0591
**B*08:01**	1.00 (0.89-1.13)	0.9687	0.89 (0.78-1.02)	0.0934
**B*15:01**	0.91 (0.75-1.09)	0.3100	0.97 (0.81-1.17)	0.7580
**B*40:01**	1.03 (0.86-1.24)	0.7371	1.06 (0.88-1.28)	0.5255
**B*44:02**	0.93 (0.81-1.07)	0.3210	0.96 (0.83-1.11)	0.5726
**B*44:03**	0.92 (0.76-1.12)	0.4057	0.99 (0.82-1.20)	0.9322
**C*03:03**	0.94 (0.78-1.14)	0.5485	1.03 (0.85-1.24)	0.7864
**C*03:04**	1.02 (0.87-1.20)	0.7963	1.02 (0.87-1.20)	0.7700
**C*04:01**	1.04 (0.89-1.21)	0.6606	0.95 (0.80-1.11)	0.5081
**C*05:01**	0.93 (0.81-1.07)	0.3381	0.99 (0.86-1.13)	0.8403
**C*06:02**	1.04 (0.90-1.21)	0.5760	0.85 (0.72-1.01)	0.0593
**C*07:01**	1.01 (0.91-1.13)	0.8048	0.89 (0.79-1.01)	0.0645
**C*07:02**	0.99 (0.88-1.11)	0.8682	1.09 (0.97-1.23)	0.1485
**DPA1*01:03**	1.09 (0.97-1.22)	0.1370	1.07 (0.96-1.21)	0.2287
**DPA1*02:01**	0.94 (0.83-1.06)	0.3073	0.93 (0.82-1.06)	0.2652
**DPB1*01:01**	0.90 (0.74-1.09)	0.2677	0.82 (0.67-0.99)	0.0548
**DPB1*02:01**	1.01 (0.88-1.15)	0.9306	1.01 (0.88-1.16)	0.8637
**DPB1*03:01**	1.08 (0.94-1.24)	0.2648	1.09 (0.95-1.25)	0.2398
**DPB1*04:01**	1.06 (0.97-1.15)	0.1949	1.00 (0.92-1.09)	0.9761
**DPB1*04:02**	0.88 (0.76-1.02)	0.0917	0.94 (0.81-1.09)	0.4043
**DQA1*01:01**	1.03 (0.91-1.17)	0.6058	1.04 (0.92-1.18)	0.5272
**DQA1*01:02**	1.02 (0.92-1.14)	0.6711	1.17 (1.06-1.31)	0.0032
**DQA1*01:03**	0.90 (0.74-1.10)	0.2946	0.87 (0.71-1.07)	0.1796
**DQA1*02:01**	0.97 (0.86-1.10)	0.6419	0.84 (0.74-0.96)	0.0120
**DQA1*03:01**	0.93 (0.83-1.04)	0.1963	1.00 (0.90-1.12)	0.9979
**DQA1*05:01**	1.10 (0.99-1.21)	0.0729	1.01 (0.91-1.12)	0.8767
**DQB1*02:01**	1.06 (0.94-1.20)	0.3143	0.98 (0.86-1.10)	0.6911
**DQB1*02:02**	0.95 (0.83-1.10)	0.5174	0.89 (0.76-1.03)	0.1184
**DQB1*03:01**	1.02 (0.91-1.14)	0.7091	1.07 (0.96-1.20)	0.2197
**DQB1*03:02**	0.94 (0.82-1.09)	0.4362	0.96 (0.82-1.11)	0.5452
**DQB1*03:03**	1.08 (0.90-1.30)	0.4250	0.82 (0.66-1.02)	0.0774
**DQB1*05:01**	1.05 (0.92-1.20)	0.4438	1.06 (0.92-1.20)	0.4266
**DQB1*06:02**	1.09 (0.97-1.23)	0.1454	1.17 (1.04-1.32)	0.0103
**DQB1*06:03**	0.86 (0.70-1.06)	0.1560	0.88 (0.72-1.09)	0.2447
**DRB1*01:01**	1.04 (0.90-1.20)	0.6051	1.12 (0.97-1.29)	0.1228
**DRB1*03:01**	1.07 (0.95-1.20)	0.2750	0.98 (0.87-1.11)	0.7933
**DRB1*04:01**	0.87 (0.76-1.01)	0.0625	0.97 (0.85-1.12)	0.6969
**DRB1*07:01**	0.97 (0.85-1.09)	0.5832	0.85 (0.75-0.97)	0.0184
**DRB1*13:01**	0.90 (0.73-1.11)	0.3207	0.87 (0.70-1.08)	0.2070

HLA alleles	Skin cancer			
	Men		Women	
	Adjusted OR (95%CI) [a]	*P* value [b]	Adjusted OR (95%CI) [a]	*P* value [b]
**DRB1*15:01**	1.08 (0.96-1.22)	0.1797	1.13 (1.00-1.28)	0.0418
**DRB3*01:01**	1.02 (0.91-1.15)	0.6988	0.88 (0.78-0.99)	0.0508
**DRB3*02:02**	1.07 (0.94-1.21)	0.3100	1.09 (0.96-1.23)	0.2029
**DRB4*01:01**	0.86 (0.73-1.02)	0.0817	0.89 (0.76-1.05)	0.1820
**DRB4*01:03**	0.98 (0.89-1.09)	0.7389	0.91 (0.83-1.01)	0.0893
**DRB5*01:01**	1.09 (0.97-1.23)	0.1475	1.15 (1.02-1.29)	0.0266

Abbreviations: CI, confidence interval; HLA, human leukocyte antigen; OR, odds ratio.

[a] Adjusted ORs and 95%CIs were computed from the multivariable-adjusted logistic regression model adjusted for age at recruitment, assessment center, and population structure (top 5 principal components of the genotypes) by using dosage of each HLA allele as a continuous variable.

[b] P<0.001 (0.05/50) was considered statistically significance in this analysis after Bonferroni correction.

Supplementary Table S12. Multivariable-adjusted associations between each HLA allele and incident risk of hematologic cancers

HLA alleles	non-Hodgkin lymphoma				Leukemia				Multiple myeloma	
	Men		Women		Men		Women		Men	
	Adjusted OR (95%CI) [a]	*P* value	Adjusted OR (95%CI) [a]	*P* value	Adjusted OR (95%CI) [a]	*P* value	Adjusted OR (95%CI) [a]	*P* value	Adjusted OR (95%CI) [a]	*P* value
**A*01:01**	1.12 (0.98-1.27)	0.0848	1.05 (0.91-1.21)	0.4869	0.93 (0.80-1.09)	0.4003	1.10 (0.91-1.33)	0.3251	1.05 (0.86-1.28)	0.6386
**A*02:01**	1.06 (0.95-1.19)	0.2904	0.95 (0.84-1.08)	0.4535	1.02 (0.89-1.17)	0.7459	1.08 (0.91-1.28)	0.3656	1.00 (0.84-1.20)	0.9804
**A*03:01**	0.92 (0.79-1.07)	0.3035	1.10 (0.94-1.28)	0.2416	0.90 (0.76-1.08)	0.2682	1.08 (0.87-1.34)	0.4734	0.98 (0.78-1.22)	0.8324
**A*11:01**	1.08 (0.87-1.33)	0.4789	1.09 (0.87-1.37)	0.4571	1.12 (0.87-1.42)	0.3821	0.89 (0.63-1.25)	0.5067	0.79 (0.55-1.14)	0.2086
**A*24:02**	0.88 (0.71-1.09)	0.2348	1.17 (0.96-1.44)	0.1202	0.90 (0.71-1.15)	0.4125	0.91 (0.67-1.23)	0.5277	1.01 (0.75-1.36)	0.9369
**B*07:02**	0.92 (0.79-1.07)	0.2838	1.08 (0.93-1.26)	0.3186	1.10 (0.93-1.30)	0.2652	1.02 (0.83-1.27)	0.8309	1.31 (1.07-1.60)	0.0092
**B*08:01**	1.20 (1.04-1.38)	0.0102	1.20 (1.03-1.39)	0.0208	1.18 (0.99-1.39)	0.0537	1.25 (1.02-1.53)	0.0333	1.01 (0.80-1.26)	0.9540
**B*15:01**	1.23 (1.01-1.49)	0.0434	0.80 (0.62-1.03)	0.0860	0.66 (0.49-0.89)	0.0071	1.03 (0.75-1.41)	0.8486	1.29 (0.96-1.72)	0.0864
**B*40:01**	0.95 (0.76-1.20)	0.6845	0.89 (0.69-1.16)	0.3995	1.02 (0.78-1.32)	0.8976	0.90 (0.64-1.28)	0.5644	0.61 (0.40-0.94)	0.0241
**B*44:02**	0.99 (0.84-1.16)	0.8707	0.92 (0.77-1.11)	0.3904	0.91 (0.75-1.12)	0.3763	0.87 (0.67-1.12)	0.2780	0.76 (0.58-1.01)	0.0552
**B*44:03**	1.02 (0.82-1.27)	0.8311	1.00 (0.79-1.27)	0.9968	0.99 (0.77-1.29)	0.9541	1.01 (0.73-1.39)	0.9604	0.95 (0.67-1.33)	0.7556
**C*03:03**	0.97 (0.77-1.22)	0.7838	0.66 (0.49-0.89)	0.0058	0.74 (0.54-1.00)	0.0467	0.82 (0.57-1.19)	0.2943	1.08 (0.78-1.51)	0.6294
**C*03:04**	1.08 (0.89-1.30)	0.4350	0.87 (0.70-1.08)	0.2151	1.01 (0.80-1.26)	0.9642	0.95 (0.71-1.27)	0.7286	0.92 (0.69-1.24)	0.5983
**C*04:01**	1.22 (1.02-1.45)	0.0291	1.22 (1.01-1.47)	0.0380	1.00 (0.80-1.25)	0.9962	0.91 (0.69-1.22)	0.5452	0.98 (0.73-1.30)	0.8853
**C*05:01**	1.02 (0.87-1.20)	0.7891	0.91 (0.76-1.09)	0.3167	0.93 (0.76-1.13)	0.4693	0.91 (0.71-1.17)	0.4718	0.77 (0.58-1.01)	0.0592
**C*06:02**	1.04 (0.87-1.24)	0.6792	0.97 (0.80-1.19)	0.8022	1.02 (0.82-1.25)	0.8898	0.83 (0.62-1.11)	0.2058	0.82 (0.61-1.11)	0.1979
**C*07:01**	1.14 (1.00-1.30)	0.0493	1.10 (0.95-1.27)	0.1906	1.15 (0.98-1.34)	0.0816	1.20 (0.99-1.45)	0.0635	1.04 (0.85-1.27)	0.7132
**C*07:02**	0.91 (0.79-1.06)	0.2235	1.07 (0.92-1.25)	0.3558	1.10 (0.94-1.29)	0.2490	1.00 (0.81-1.23)	0.9997	1.26 (1.03-1.54)	0.0239
**DPA1*01:03**	0.97 (0.85-1.11)	0.6389	1.01 (0.87-1.17)	0.8742	1.11 (0.94-1.30)	0.2111	1.01 (0.83-1.23)	0.9161	1.08 (0.88-1.32)	0.4845
**DPA1*02:01**	1.02 (0.89-1.18)	0.7443	0.98 (0.83-1.15)	0.7945	0.89 (0.75-1.07)	0.2176	0.95 (0.76-1.19)	0.6480	0.94 (0.75-1.18)	0.5730
**DPB1*01:01**	1.11 (0.90-1.36)	0.3480	1.12 (0.89-1.41)	0.3201	0.85 (0.65-1.12)	0.2627	1.18 (0.87-1.59)	0.2951	1.10 (0.80-1.51)	0.5553
**DPB1*02:01**	0.92 (0.78-1.10)	0.3647	0.87 (0.72-1.06)	0.1669	1.25 (1.04-1.49)	0.0146	1.03 (0.81-1.31)	0.8349	0.98 (0.76-1.26)	0.8847
**DPB1*03:01**	1.00 (0.84-1.19)	0.9751	0.98 (0.81-1.18)	0.8256	0.93 (0.75-1.14)	0.4788	1.00 (0.78-1.29)	0.9822	1.15 (0.90-1.47)	0.2578
**DPB1*04:01**	1.05 (0.95-1.16)	0.3686	1.07 (0.95-1.20)	0.2536	1.02 (0.90-1.15)	0.7609	1.03 (0.88-1.20)	0.7542	1.00 (0.86-1.18)	0.9611
**DPB1*04:02**	0.92 (0.77-1.09)	0.3188	1.11 (0.93-1.32)	0.2630	1.00 (0.82-1.22)	0.9925	0.90 (0.70-1.17)	0.4337	1.04 (0.81-1.33)	0.7739
**DQA1*01:01**	1.15 (0.99-1.33)	0.0545	1.15 (0.99-1.34)	0.0691	0.93 (0.78-1.12)	0.4511	0.90 (0.72-1.13)	0.3634	1.03 (0.82-1.29)	0.8039
**DQA1*01:02**	0.89 (0.78-1.02)	0.1077	0.97 (0.84-1.12)	0.6525	0.91 (0.78-1.07)	0.2402	0.88 (0.72-1.08)	0.2252	1.03 (0.85-1.25)	0.7656
**DQA1*01:03**	0.98 (0.78-1.23)	0.8440	0.68 (0.50-0.91)	0.0090	0.98 (0.75-1.28)	0.8770	1.03 (0.74-1.44)	0.8513	1.33 (0.98-1.80)	0.0656
**DQA1*02:01**	1.06 (0.91-1.22)	0.4526	1.07 (0.92-1.25)	0.3873	0.96 (0.80-1.14)	0.6370	0.99 (0.80-1.23)	0.9348	0.92 (0.73-1.16)	0.4862
**DQA1*03:01**	0.91 (0.80-1.04)	0.1572	0.80 (0.69-0.93)	0.0042	1.09 (0.94-1.26)	0.2514	1.07 (0.89-1.29)	0.4688	0.82 (0.67-1.01)	0.0595
**DQA1*05:01**	1.10 (0.97-1.24)	0.1261	1.18 (1.04-1.34)	0.0116	1.13 (0.98-1.29)	0.0979	1.08 (0.90-1.29)	0.3958	1.08 (0.90-1.30)	0.3969
**DQB1*02:01**	1.24 (1.08-1.42)	0.0024	1.19 (1.03-1.38)	0.0209	1.17 (0.99-1.38)	0.0563	1.20 (0.98-1.47)	0.0847	1.08 (0.87-1.34)	0.4689
**DQB1*02:02**	1.01 (0.86-1.20)	0.8806	1.01 (0.84-1.21)	0.9413	0.94 (0.76-1.15)	0.5238	1.01 (0.79-1.30)	0.9139	0.93 (0.71-1.21)	0.5862

HLA alleles	non-Hodgkin lymphoma				Leukemia				Multiple myeloma	
	Men		Women		Men		Women		Men	
	Adjusted OR (95%CI) [a]	*P* value	Adjusted OR (95%CI) [a]	*P* value	Adjusted OR (95%CI) [a]	*P* value	Adjusted OR (95%CI) [a]	*P* value	Adjusted OR (95%CI) [a]	*P* value
**DQB1*03:01**	0.87 (0.76-1.01)	0.0598	0.96 (0.82-1.11)	0.5642	1.12 (0.96-1.31)	0.1433	0.93 (0.76-1.15)	0.5124	0.94 (0.76-1.16)	0.5814
**DQB1*03:02**	0.90 (0.76-1.08)	0.2680	0.81 (0.66-0.99)	0.0363	0.94 (0.77-1.16)	0.5719	1.06 (0.83-1.36)	0.6451	0.91 (0.70-1.19)	0.4842
**DQB1*03:03**	1.10 (0.88-1.37)	0.4243	1.09 (0.86-1.39)	0.4756	0.98 (0.74-1.29)	0.8689	1.03 (0.73-1.44)	0.8800	0.82 (0.56-1.21)	0.3258
**DQB1*05:01**	1.27 (1.09-1.47)	0.0017	1.26 (1.07-1.48)	0.0044	0.90 (0.74-1.09)	0.2905	0.96 (0.75-1.21)	0.7085	0.98 (0.77-1.25)	0.8667
**DQB1*06:02**	0.96 (0.82-1.11)	0.5719	1.02 (0.87-1.20)	0.8356	1.00 (0.84-1.19)	0.9912	0.86 (0.68-1.09)	0.2202	1.17 (0.95-1.45)	0.1364
**DQB1*06:03**	0.98 (0.77-1.24)	0.8411	0.72 (0.54-0.97)	0.0286	0.91 (0.68-1.22)	0.5346	1.03 (0.73-1.45)	0.8534	1.30 (0.94-1.78)	0.1093
**DRB1*01:01**	1.35 (1.15-1.58)	**0.0002** [*]	1.16 (0.97-1.40)	0.0986	0.75 (0.60-0.95)	0.0189	0.94 (0.72-1.23)	0.6718	0.96 (0.73-1.27)	0.7815
**DRB1*03:01**	1.22 (1.06-1.40)	0.0048	1.20 (1.03-1.39)	0.0167	1.17 (0.99-1.38)	0.0563	1.22 (1.00-1.50)	0.0494	1.05 (0.85-1.31)	0.6501
**DRB1*04:01**	0.92 (0.78-1.09)	0.3561	0.90 (0.75-1.08)	0.2581	1.17 (0.98-1.40)	0.0824	1.01 (0.80-1.29)	0.9196	0.85 (0.66-1.11)	0.2337
**DRB1*07:01**	1.06 (0.92-1.22)	0.4427	1.08 (0.92-1.26)	0.3346	0.96 (0.81-1.15)	0.6757	0.99 (0.80-1.23)	0.9463	0.92 (0.73-1.16)	0.4924
**DRB1*13:01**	0.96 (0.76-1.22)	0.7470	0.67 (0.49-0.91)	0.0103	0.94 (0.71-1.26)	0.6933	1.00 (0.70-1.42)	0.9893	1.37 (1.01-1.87)	0.0449
**DRB1*15:01**	0.93 (0.80-1.08)	0.3286	1.03 (0.88-1.20)	0.7473	1.00 (0.84-1.18)	0.9787	0.88 (0.70-1.11)	0.2811	1.19 (0.97-1.47)	0.1034
**DRB3*01:01**	1.15 (1.01-1.32)	0.0363	1.06 (0.92-1.23)	0.4230	1.11 (0.95-1.30)	0.1778	1.16 (0.96-1.41)	0.1333	1.11 (0.90-1.36)	0.3279
**DRB3*02:02**	0.85 (0.73-0.99)	0.0540	0.96 (0.81-1.14)	0.6339	1.05 (0.88-1.25)	0.6073	0.82 (0.64-1.04)	0.1037	1.10 (0.88-1.37)	0.4114
**DRB4*01:01**	1.04 (0.87-1.25)	0.6658	1.00 (0.82-1.22)	0.9946	0.90 (0.71-1.13)	0.3647	0.96 (0.72-1.27)	0.7765	0.97 (0.73-1.29)	0.8303
**DRB4*01:03**	0.94 (0.83-1.06)	0.2978	0.90 (0.78-1.02)	0.1021	1.10 (0.96-1.26)	0.1839	1.04 (0.88-1.24)	0.6468	0.82 (0.68-0.99)	0.0343
**DRB5*01:01**	0.93 (0.80-1.08)	0.3171	1.03 (0.88-1.21)	0.7046	1.00 (0.84-1.19)	0.9810	0.86 (0.68-1.09)	0.2067	1.17 (0.95-1.44)	0.1476

Abbreviations: CI, confidence interval; HLA, human leukocyte antigen; OR, odds ratio.

[a] Adjusted ORs and 95%CIs were computed from the multivariable-adjusted logistic regression model adjusted for age at recruitment, assessment center, and population structure (top 5 principal components of the genotypes) by using dosage of each HLA allele as a continuous variable.

* P <0.001 (0.05/50) was considered statistically significance in this analysis after Bonferroni correction, which was shown in bold.

Supplementary Table S13. Multivariable-adjusted associations between each HLA allele and incident risk of female reproductive cancers

HLA alleles	Endometrial cancer		Ovarian cancer	
	Women		Women	
	Adjusted OR (95%CI) [a]	*P* value	Adjusted OR (95%CI) [a]	*P* value
**A*01:01**	0.92 (0.82-1.04)	0.1810	1.03 (0.90-1.18)	0.6625
**A*02:01**	0.96 (0.87-1.06)	0.4613	0.98 (0.87-1.11)	0.7489
**A*03:01**	1.10 (0.98-1.25)	0.1083	0.95 (0.81-1.11)	0.4900
**A*11:01**	0.91 (0.74-1.10)	0.3158	0.99 (0.78-1.25)	0.9103
**A*24:02**	1.08 (0.92-1.27)	0.3507	1.00 (0.81-1.23)	0.9849
**B*07:02**	1.23 (1.09-1.38)	**0.0006**[*]	1.05 (0.90-1.23)	0.5047
**B*08:01**	0.95 (0.84-1.08)	0.4673	0.99 (0.84-1.16)	0.8860
**B*15:01**	0.87 (0.71-1.05)	0.1453	1.24 (1.01-1.53)	0.0384
**B*40:01**	0.97 (0.80-1.18)	0.7928	1.08 (0.85-1.36)	0.5317
**B*44:02**	0.94 (0.81-1.08)	0.3918	0.81 (0.67-0.98)	0.0275
**B*44:03**	0.95 (0.78-1.15)	0.5872	1.08 (0.86-1.35)	0.5167
**C*03:03**	0.86 (0.70-1.06)	0.1583	1.17 (0.94-1.47)	0.1649
**C*03:04**	1.01 (0.86-1.19)	0.9225	1.10 (0.91-1.34)	0.3194
**C*04:01**	1.03 (0.88-1.20)	0.7527	0.95 (0.78-1.16)	0.6224
**C*05:01**	0.87 (0.75-1.01)	0.0614	0.87 (0.72-1.04)	0.1262
**C*06:02**	0.93 (0.79-1.09)	0.3844	0.99 (0.81-1.20)	0.8942
**C*07:01**	0.95 (0.84-1.07)	0.3771	0.94 (0.81-1.09)	0.4189
**C*07:02**	1.19 (1.06-1.34)	0.0027	1.03 (0.89-1.19)	0.7148
**DPA1*01:03**	0.99 (0.88-1.10)	0.8046	0.95 (0.83-1.10)	0.4976
**DPA1*02:01**	1.02 (0.90-1.15)	0.8101	1.09 (0.94-1.27)	0.2606
**DPB1*01:01**	0.90 (0.74-1.09)	0.2797	1.06 (0.85-1.33)	0.6010
**DPB1*02:01**	1.10 (0.96-1.26)	0.1813	0.91 (0.75-1.09)	0.2872
**DPB1*03:01**	0.97 (0.83-1.12)	0.6740	0.85 (0.70-1.03)	0.1021
**DPB1*04:01**	0.96 (0.88-1.05)	0.3562	1.05 (0.94-1.17)	0.3927
**DPB1*04:02**	1.02 (0.89-1.18)	0.7432	0.94 (0.78-1.12)	0.4789
**DQA1*01:01**	1.18 (1.05-1.33)	0.0055	0.89 (0.75-1.05)	0.1541
**DQA1*01:02**	1.12 (1.01-1.25)	0.0351	1.11 (0.97-1.28)	0.1156
**DQA1*01:03**	1.09 (0.90-1.31)	0.3730	0.91 (0.71-1.17)	0.4621
**DQA1*02:01**	0.96 (0.85-1.09)	0.5512	1.09 (0.93-1.26)	0.2793
**DQA1*03:01**	0.84 (0.75-0.95)	0.0043	0.96 (0.84-1.11)	0.6049
**DQA1*05:01**	0.94 (0.84-1.04)	0.2269	1.00 (0.88-1.14)	0.9535
**DQB1*02:01**	0.92 (0.81-1.04)	0.1760	1.07 (0.92-1.25)	0.3597
**DQB1*02:02**	0.97 (0.83-1.12)	0.6487	1.04 (0.87-1.24)	0.6703
**DQB1*03:01**	0.88 (0.78-0.99)	0.0353	0.84 (0.72-0.98)	0.0252
**DQB1*03:02**	0.95 (0.82-1.10)	0.4767	1.03 (0.86-1.23)	0.7600
**DQB1*03:03**	0.92 (0.74-1.13)	0.4006	1.10 (0.87-1.39)	0.4322
**DQB1*05:01**	1.13 (0.99-1.29)	0.0593	0.91 (0.76-1.08)	0.2785
**DQB1*06:02**	1.11 (0.98-1.25)	0.1054	1.10 (0.94-1.28)	0.2363
**DQB1*06:03**	1.11 (0.92-1.35)	0.2653	0.88 (0.67-1.14)	0.3209
**DRB1*01:01**	1.15 (0.99-1.32)	0.0623	0.88 (0.73-1.08)	0.2199
**DRB1*03:01**	0.90 (0.79-1.03)	0.1195	1.09 (0.94-1.26)	0.2769
**DRB1*04:01**	0.83 (0.71-0.96)	0.0146	0.93 (0.78-1.11)	0.4282
**DRB1*07:01**	0.96 (0.84-1.09)	0.5018	1.09 (0.94-1.27)	0.2600
**DRB1*13:01**	1.08 (0.89-1.31)	0.4573	0.93 (0.71-1.20)	0.5658

HLA alleles	Endometrial cancer		Ovarian cancer	
	Women		Women	
	**Adjusted OR (95%CI) [a]**	***P* value**	**Adjusted OR (95%CI) [a]**	***P* value**
**DRB1*15:01**	1.12 (0.99-1.26)	0.0782	1.09 (0.94-1.27)	0.2693
**DRB3*01:01**	0.98 (0.87-1.10)	0.7449	1.02 (0.88-1.18)	0.7752
**DRB3*02:02**	1.01 (0.89-1.15)	0.8741	0.97 (0.82-1.14)	0.6961
**DRB4*01:01**	1.01 (0.86-1.18)	0.9429	1.02 (0.84-1.24)	0.8468
**DRB4*01:03**	0.87 (0.79-0.97)	0.0118	1.02 (0.90-1.15)	0.7687
**DRB5*01:01**	1.11 (0.98-1.25)	0.0999	1.07 (0.92-1.25)	0.3570

Abbreviations: CI, confidence interval; HLA, human leukocyte antigen; OR, odds ratio.

[a] Adjusted ORs and 95%CIs were computed from the multivariable-adjusted logistic regression model adjusted for age at recruitment, assessment center, and population structure (top 5 principal components of the genotypes) by using dosage of each HLA allele as a continuous variable.

* P<0.001 (0.05/50) was considered statistically significance in this analysis after Bonferroni correction, which was shown in bold.

Supplementary Table S14. Multivariable-adjusted associations between each HLA allele and incident risk of brain cancer

HLA alleles	Brain cancer	
	Men	
	Adjusted OR (95%CI) [a]	*P* value [b]
**A*01:01**	1.06 (0.87-1.28)	0.5821
**A*02:01**	0.95 (0.80-1.13)	0.5447
**A*03:01**	1.02 (0.82-1.27)	0.8373
**A*11:01**	1.07 (0.78-1.46)	0.6858
**A*24:02**	1.04 (0.78-1.39)	0.7864
**B*07:02**	0.79 (0.62-0.99)	0.0544
**B*08:01**	1.00 (0.81-1.25)	0.9754
**B*15:01**	0.87 (0.62-1.23)	0.4352
**B*40:01**	1.31 (0.97-1.77)	0.0740
**B*44:02**	0.98 (0.77-1.26)	0.8918
**B*44:03**	0.84 (0.59-1.20)	0.3397
**C*03:03**	1.00 (0.71-1.40)	0.9985
**C*03:04**	1.12 (0.86-1.48)	0.4013
**C*04:01**	1.17 (0.90-1.52)	0.2431
**C*05:01**	1.06 (0.83-1.34)	0.6408
**C*06:02**	0.97 (0.74-1.28)	0.8525
**C*07:01**	1.06 (0.86-1.29)	0.5933
**C*07:02**	0.79 (0.62-0.99)	0.0420
**DPA1*01:03**	1.11 (0.90-1.36)	0.3352
**DPA1*02:01**	0.90 (0.72-1.13)	0.3634
**DPB1*01:01**	0.77 (0.53-1.11)	0.1644
**DPB1*02:01**	1.17 (0.93-1.47)	0.1890
**DPB1*03:01**	0.80 (0.60-1.07)	0.1342
**DPB1*04:01**	0.97 (0.83-1.13)	0.6913
**DPB1*04:02**	1.27 (1.01-1.60)	0.0407
**DQA1*01:01**	1.04 (0.83-1.29)	0.7467
**DQA1*01:02**	1.12 (0.93-1.36)	0.2290
**DQA1*01:03**	1.02 (0.73-1.43)	0.9119
**DQA1*02:01**	0.81 (0.64-1.02)	0.0747
**DQA1*03:01**	0.90 (0.74-1.09)	0.2871
**DQA1*05:01**	1.17 (0.98-1.39)	0.0889
**DQB1*02:01**	0.97 (0.78-1.21)	0.7850
**DQB1*02:02**	0.71 (0.53-0.96)	0.0238
**DQB1*03:01**	1.09 (0.90-1.33)	0.3893
**DQB1*03:02**	1.02 (0.79-1.31)	0.8889
**DQB1*03:03**	1.04 (0.74-1.47)	0.8043
**DQB1*05:01**	1.04 (0.82-1.32)	0.7336
**DQB1*06:02**	1.02 (0.82-1.27)	0.8687
**DQB1*06:03**	1.00 (0.70-1.42)	0.9907
**DRB1*01:01**	1.10 (0.85-1.42)	0.4540
**DRB1*03:01**	0.97 (0.78-1.21)	0.7863
**DRB1*04:01**	0.89 (0.69-1.15)	0.3802
**DRB1*07:01**	0.82 (0.65-1.04)	0.1041
**DRB1*13:01**	0.98 (0.69-1.41)	0.9334

HLA alleles	Brain cancer	
	Men	
	**Adjusted OR (95%CI) [a]**	***P* value [b]**
**DRB1*15:01**	1.01 (0.81-1.26)	0.9123
**DRB3*01:01**	0.96 (0.78-1.19)	0.7373
**DRB3*02:02**	1.30 (1.06-1.61)	0.0120
**DRB4*01:01**	0.72 (0.52-1.00)	0.0471
**DRB4*01:03**	0.97 (0.82-1.16)	0.7745
**DRB5*01:01**	1.03 (0.83-1.28)	0.7948

Abbreviations: CI, confidence interval; HLA, human leukocyte antigen; OR, odds ratio.

[a] Adjusted ORs and 95%CIs were computed from the multivariable-adjusted logistic regression model adjusted for age at recruitment, assessment center, and population structure (top 5 principal components of the genotypes) by using dosage of each HLA allele as a continuous variable.

[b] P<0.001 (0.05/50) was considered statistically significance in this analysis after Bonferroni correction.

Supplementary Table S15. Multivariable-adjusted associations between each HLA allele and incident risk of other cancers*

HLA alleles	Men		Women	
	Adjusted OR (95%CI) [a]	*P* value [b]	Adjusted OR (95%CI) [a]	*P* value [b]
**A*01:01**	1.02 (0.95-1.11)	0.5421	1.05 (0.98-1.12)	0.1851
**A*02:01**	0.95 (0.89-1.02)	0.1677	0.99 (0.93-1.05)	0.6898
**A*03:01**	0.98 (0.89-1.07)	0.6109	0.96 (0.89-1.04)	0.3278
**A*11:01**	0.94 (0.83-1.08)	0.3987	1.03 (0.92-1.15)	0.5894
**A*24:02**	1.02 (0.90-1.15)	0.7743	0.98 (0.88-1.09)	0.7344
**B*07:02**	0.96 (0.88-1.05)	0.3842	1.08 (1.00-1.16)	0.0467
**B*08:01**	1.04 (0.95-1.13)	0.4234	1.01 (0.93-1.09)	0.8603
**B*15:01**	0.93 (0.82-1.07)	0.3075	0.91 (0.81-1.02)	0.1132
**B*40:01**	0.95 (0.83-1.09)	0.4722	1.06 (0.94-1.18)	0.3626
**B*44:02**	1.04 (0.94-1.14)	0.4621	1.03 (0.95-1.12)	0.5048
**B*44:03**	1.08 (0.95-1.23)	0.2395	1.10 (0.98-1.22)	0.1087
**C*03:03**	1.01 (0.88-1.15)	0.9034	0.94 (0.83-1.06)	0.3258
**C*03:04**	0.93 (0.82-1.05)	0.2182	1.00 (0.90-1.10)	0.9498
**C*04:01**	1.06 (0.95-1.19)	0.2656	0.93 (0.84-1.03)	0.1424
**C*05:01**	1.05 (0.95-1.15)	0.3646	0.99 (0.91-1.08)	0.8053
**C*06:02**	0.94 (0.84-1.05)	0.3093	1.02 (0.93-1.13)	0.6077
**C*07:01**	1.03 (0.95-1.11)	0.5003	1.04 (0.97-1.11)	0.3237
**C*07:02**	0.96 (0.88-1.05)	0.3784	1.05 (0.97-1.13)	0.2251
**DPA1*01:03**	0.99 (0.91-1.07)	0.7316	0.97 (0.91-1.04)	0.3987
**DPA1*02:01**	1.02 (0.93-1.11)	0.6598	1.03 (0.96-1.11)	0.4042
**DPB1*01:01**	0.96 (0.84-1.10)	0.5813	1.07 (0.95-1.19)	0.2598
**DPB1*02:01**	1.05 (0.96-1.16)	0.2952	1.00 (0.91-1.09)	0.9193
**DPB1*03:01**	1.11 (1.00-1.23)	0.0410	1.05 (0.96-1.15)	0.2450
**DPB1*04:01**	0.97 (0.91-1.04)	0.3997	0.98 (0.93-1.04)	0.4848
**DPB1*04:02**	0.87 (0.78-0.97)	0.0112	0.92 (0.84-1.00)	0.0597
**DQA1*01:01**	1.03 (0.94-1.12)	0.5361	0.99 (0.92-1.07)	0.8496
**DQA1*01:02**	0.91 (0.84-0.99)	0.0204	0.97 (0.91-1.04)	0.4122
**DQA1*01:03**	0.92 (0.79-1.06)	0.2247	1.01 (0.90-1.14)	0.8294
**DQA1*02:01**	1.06 (0.97-1.15)	0.2173	1.05 (0.97-1.13)	0.2285
**DQA1*03:01**	1.01 (0.94-1.09)	0.7937	1.03 (0.96-1.10)	0.3898
**DQA1*05:01**	1.00 (0.93-1.08)	0.9210	0.97 (0.91-1.04)	0.3754
**DQB1*02:01**	1.04 (0.95-1.13)	0.4173	0.99 (0.92-1.07)	0.8646
**DQB1*02:02**	1.10 (0.99-1.21)	0.0656	1.07 (0.99-1.17)	0.1027
**DQB1*03:01**	1.00 (0.92-1.08)	0.9357	1.02 (0.95-1.09)	0.5991
**DQB1*03:02**	1.02 (0.92-1.13)	0.7343	0.96 (0.88-1.05)	0.3646
**DQB1*03:03**	0.94 (0.82-1.09)	0.4083	1.00 (0.89-1.13)	0.9591
**DQB1*05:01**	1.03 (0.94-1.14)	0.4829	0.99 (0.91-1.07)	0.7411
**DQB1*06:02**	0.91 (0.83-1.00)	0.0399	1.01 (0.93-1.09)	0.7998
**DQB1*06:03**	0.94 (0.81-1.09)	0.3892	0.98 (0.87-1.11)	0.7953
**DRB1*01:01**	1.02 (0.92-1.13)	0.7163	0.95 (0.87-1.05)	0.3156
**DRB1*03:01**	1.04 (0.95-1.13)	0.3728	0.98 (0.91-1.06)	0.6342
**DRB1*04:01**	1.01 (0.92-1.11)	0.8199	0.99 (0.91-1.08)	0.8394
**DRB1*07:01**	1.04 (0.95-1.13)	0.3739	1.05 (0.97-1.13)	0.2000
**DRB1*13:01**	0.94 (0.81-1.08)	0.3804	1.02 (0.90-1.15)	0.8111
**DRB1*15:01**	0.91 (0.83-1.00)	0.0429	1.00 (0.93-1.08)	0.9572

HLA alleles	Men		Women	
	Adjusted OR (95%CI) [a]	*P* value [b]	Adjusted OR (95%CI) [a]	*P* value [b]
**DRB3*01:01**	1.02 (0.93-1.10)	0.7240	0.98 (0.91-1.05)	0.5293
**DRB3*02:02**	0.96 (0.87-1.05)	0.3781	1.00 (0.93-1.09)	0.9156
**DRB4*01:01**	1.07 (0.95-1.19)	0.2574	1.07 (0.97-1.17)	0.1910
**DRB4*01:03**	1.03 (0.96-1.10)	0.4193	1.02 (0.96-1.08)	0.5703
**DRB5*01:01**	0.91 (0.83-1.00)	0.0427	1.00 (0.92-1.08)	0.9808

Abbreviations: CI, confidence interval; HLA, human leukocyte antigen; OR, odds ratio.

[a] Adjusted ORs and 95%CIs were computed from the multivariable-adjusted logistic regression model adjusted for age at recruitment, assessment center, and population structure (top 5 principal components of the genotypes) by using dosage of each HLA allele as a continuous variable.

[b] *P* <0.001 (0.05/50) was considered statistically significance in this analysis after Bonferroni correction.

*Other cancers referred to all malignant cancers except the studied cancers in this study, 14 cancer types for men and 10 for women

Supplementary Table S16. P values of multiplicative interactions between HLA allele carrier status and lifestyle on the incident risk of all cancer outcomes among men [a]

HLA allele	Prostate cancer	Colorectal cancer	Lung cancer	Skin cancer	non-Hodgkin lymphoma	Kidney cancer	Bladder cancer	Esophageal cancer	Leukemia	Pancreatic cancer	Gastric cancer	Brain cancer	Multiple myeloma	Liver cancer	Other cancers	Overall cancer
A*01:01	0.9995	0.8321	0.6292	0.1444	0.9012	0.5008	0.9878	0.7836	0.8865	0.2611	0.6724	0.1467	0.1590	0.6140	0.1272	0.2046
A*02:01	0.0095	0.7878	0.2943	0.8767	0.0765	0.0269	0.3766	0.7881	0.2622	0.6859	0.8334	0.4415	0.1737	0.9544	0.7474	0.4167
A*03:01	0.7879	0.4162	0.6601	0.0092	0.0256	0.1699	0.5344	0.4444	0.3827	0.4219	0.4780	0.7401	0.0767	0.4617	0.7359	0.9106
A*11:01	0.2017	0.2875	0.7930	0.8260	0.5953	0.0477	0.1080	0.4217	0.3919	0.9580	0.7547	0.0541	0.3300	0.9459	0.5020	0.1557
A*24:02	0.0641	0.0903	0.6260	0.4650	0.6761	0.7372	0.1519	0.8059	0.8662	0.9543	0.6497	0.2393	0.7959	0.8505	0.3259	0.4972
B*07:02	0.8392	0.2176	0.9070	0.9873	0.0196	0.3335	0.9526	0.8988	0.1816	0.9014	0.7410	0.6845	0.1376	0.6140	0.4373	0.3406
B*08:01	0.5880	0.9110	0.0775	0.0183	0.0173	0.2340	0.5798	0.9074	0.4034	0.4475	0.2745	0.3301	0.9998	0.8935	0.4039	**0.0004***
B*15:01	0.2693	0.3468	0.7136	0.6004	0.2469	0.8516	0.9154	0.7011	0.7742	0.3075	0.3235	0.6033	0.4858	0.1188	0.4515	0.9731
B*40:01	0.0672	0.9778	0.7338	0.0736	0.5120	0.8024	0.6325	0.4561	0.3127	0.9985	0.1460	0.2707	0.9396	0.4996	0.0563	0.0049
B*44:02	0.1102	0.6766	0.9423	0.7329	0.9839	0.2961	0.4970	0.9792	0.4744	0.4113	0.0657	0.4719	0.8242	0.1441	0.9106	0.3831
B*44:03	0.2911	0.6132	0.7886	0.7137	0.8880	0.9548	0.0421	0.6174	0.5946	0.5574	0.7800	0.7755	0.6077	0.6464	0.8483	0.2964
C*03:03	0.6167	0.1421	0.7971	0.4080	0.4030	0.5930	0.6601	0.8526	0.0692	0.8781	0.7347	0.3489	0.6234	0.0209	0.9355	0.8541
C*03:04	0.2850	0.9724	0.4307	0.1263	0.2428	0.4825	0.3850	0.5889	0.3870	0.8883	0.2547	0.7342	0.4487	0.3723	0.2854	0.0460
C*04:01	0.4366	0.9372	0.9550	0.6624	0.8580	0.2917	0.1796	0.9662	0.2232	0.1130	0.9148	0.6739	0.4695	0.5398	0.5364	0.9011
C*05:01	0.5984	0.3171	0.6924	0.5669	0.5795	0.1381	0.2742	0.7128	0.5068	0.4926	0.2679	0.2085	0.9327	0.1481	0.8781	0.5140
C*06:02	0.2455	0.0666	0.0883	0.3147	0.3490	0.6205	0.7275	0.9598	0.9099	0.3690	0.0827	0.4882	0.2973	0.9965	0.6826	0.8095
C*07:01	0.7208	0.9898	0.0390	0.0320	0.0700	0.4916	0.3658	0.9701	0.2014	0.4176	0.2192	0.4112	0.2325	0.7583	0.4171	0.0056
C*07:02	0.4984	0.1140	0.9272	0.9538	0.0283	0.3871	0.8409	0.7456	0.2173	0.4847	0.6724	0.6550	0.1517	0.6331	0.5656	0.1522
DPA1*01:03	0.5968	0.6880	0.2099	0.1834	0.7877	0.3137	0.5925	0.9555	0.5538	0.7786	0.6018	0.4458	0.9960	0.9988	0.7271	0.1585
DPA1*02:01	0.9052	0.1253	0.5599	0.9068	0.8476	0.5449	0.2453	0.5473	0.2221	0.3546	0.1700	0.4003	0.3277	0.4715	0.9785	0.9558
DPB1*01:01	0.9005	0.2981	0.5575	0.1803	0.5478	0.7719	0.8311	0.3189	0.4454	0.6832	0.5467	0.3330	0.7378	0.9912	0.1895	0.8741
DPB1*02:01	0.9371	0.6352	0.5098	0.4337	0.6042	0.2850	0.9331	0.2469	0.9666	0.3281	0.8125	0.0129	0.2259	0.2955	0.3874	0.9604
DPB1*03:01	0.1048	0.8376	0.4095	0.3135	0.1383	0.2454	0.1428	0.0388	0.9986	0.1005	0.5122	0.7920	0.8666	0.9701	0.8888	0.2876
DPB1*04:01	0.8195	0.1576	0.9491	0.7218	0.4347	0.5876	0.7781	0.4406	0.9361	0.2647	0.9895	0.5283	0.8631	0.5233	0.1221	0.9986
DPB1*04:02	0.5795	0.7959	0.9106	0.2997	0.4087	0.4469	0.6363	0.1513	0.8960	0.0244	0.9195	0.7381	0.2842	0.9670	0.4031	0.3409
DQA1*01:01	0.8119	0.1820	0.2005	0.7385	0.1711	0.2021	0.7446	0.9410	0.3056	0.1180	0.2385	0.4050	0.6686	0.1424	0.3334	0.9467
DQA1*01:02	0.2999	0.1407	0.3677	0.4840	0.6505	0.1731	0.1453	0.9860	0.0854	0.6339	0.8644	0.7253	0.6896	0.8232	0.5336	0.0481
DQA1*01:03	0.1065	0.7858	0.1008	0.0202	0.7979	0.2383	0.1550	0.4438	0.2440	0.2845	0.9652	0.3064	0.6038	0.6590	0.5404	0.3281
DQA1*02:01	0.8431	0.4513	0.8038	0.7372	0.8370	0.1437	0.2935	0.8246	0.9078	0.2591	0.9748	0.4621	0.5279	0.5839	0.8282	0.7565
DQA1*03:01	0.2036	0.2757	0.9206	0.3211	0.8534	0.5023	0.0563	0.7879	0.4277	0.4997	0.3233	0.1297	0.9478	0.5340	0.3331	0.0241
DQA1*05:01	0.4173	0.8014	0.0077	0.0361	0.2084	0.5177	0.3939	0.5026	0.9653	0.1224	0.0621	0.4103	0.1652	0.4287	0.5253	0.0479

HLA allele	Prostate cancer	Colorectal cancer	Lung cancer	Skin cancer	non-Hodgkin lymphoma	Kidney cancer	Bladder cancer	Esophageal cancer	Leukemia	Pancreatic cancer	Gastric cancer	Brain cancer	Multiple myeloma	Liver cancer	Other cancers	Overall cancer
DQB1*02:01	0.7526	0.4139	0.1377	0.1947	0.1646	0.2561	0.9562	0.3031	0.2879	0.1095	0.1339	0.4765	0.8505	0.9521	0.4910	0.0380
DQB1*02:02	0.9638	0.3849	0.5825	0.6397	0.8431	0.2022	0.4393	0.9536	0.7782	0.7164	0.7901	0.4280	0.9521	0.8960	0.9342	0.7759
DQB1*03:01	0.6817	0.6614	0.5007	0.7424	0.5132	0.7179	0.0684	0.7366	0.9665	0.1993	0.8279	0.4673	0.0519	0.9816	0.8018	0.9995
DQB1*03:02	0.3117	0.0530	0.6501	0.0810	0.6727	0.4925	0.0100	0.4220	0.7802	0.4809	0.7063	0.9280	0.7082	0.8815	0.2755	0.0083
DQB1*03:03	0.4942	0.6890	0.0373	0.0319	0.9624	0.4195	0.6284	0.9973	0.5878	0.3277	0.9267	0.2788	0.3552	0.4291	0.4726	0.7296
DQB1*05:01	0.6100	0.2348	0.0436	0.5324	0.3914	0.3338	0.9722	0.9823	0.4455	0.0755	0.1659	0.3429	0.9086	0.1948	0.6231	0.9923
DQB1*06:02	0.4062	0.1890	0.9685	0.4501	0.4476	0.6319	0.3664	0.6378	0.0808	0.2828	0.7682	0.8413	0.9718	0.8417	0.5321	0.2557
DQB1*06:03	0.0972	0.7392	0.0770	0.0412	0.6686	0.3045	0.1359	0.7305	0.0772	0.1777	0.9990	0.2307	0.6346	0.8079	0.6373	0.5142
DRB1*01:01	0.3278	0.3544	0.3834	0.8374	0.3598	0.0441	0.7928	0.5249	0.4880	0.0398	0.4481	0.3768	0.6823	0.3787	0.8028	0.7182
DRB1*03:01	0.7997	0.4935	0.1164	0.0669	0.1152	0.1510	0.9018	0.2435	0.2795	0.2194	0.1362	0.5177	0.9331	0.9515	0.5090	0.0106
DRB1*04:01	0.7911	0.8232	0.4740	0.5334	0.6855	0.8313	0.5921	0.9354	0.8702	0.6362	0.4519	0.3555	0.9003	0.9935	0.5708	0.9309
DRB1*07:01	0.9193	0.4498	0.7831	0.6423	0.8976	0.0799	0.3159	0.8524	0.8428	0.2931	0.9926	0.4880	0.5763	0.5985	0.7781	0.8281
DRB1*13:01	0.0784	0.8721	0.0784	0.0406	0.5718	0.3781	0.1675	0.1849	0.0730	0.2278	0.9991	0.1983	0.6863	0.8452	0.6948	0.4713
DRB1*15:01	0.3214	0.4518	0.9041	0.3601	0.3826	0.5880	0.3496	0.4950	0.1530	0.2768	0.7275	0.8467	0.7052	0.8782	0.5328	0.1630
DRB3*01:01	0.5407	0.6383	0.9204	0.2075	0.1038	0.0719	0.8228	0.9749	0.1001	0.1340	0.0427	0.8711	0.4630	0.5448	0.4664	0.0755
DRB3*02:02	0.7123	0.8139	0.0558	0.8374	0.6405	0.8675	0.8490	0.5313	0.6243	0.8979	0.9192	0.6278	0.0077	0.1215	0.5280	0.7725
DRB4*01:01	0.9502	0.5086	0.3979	0.4688	0.9372	0.0098	0.5659	0.9326	0.8484	0.3027	0.4176	0.7845	0.7561	0.9754	0.8449	0.9339
DRB4*01:03	0.3424	0.2863	0.3959	0.3482	0.7677	0.7932	0.2458	0.5540	0.4370	0.9728	0.1515	0.1045	0.2584	0.9917	0.1815	0.5462
DRB5*01:01	0.2622	0.4561	0.9010	0.4694	0.2378	0.7016	0.3748	0.5032	0.1500	0.3281	0.6758	0.8483	0.7184	0.8803	0.5805	0.1731

Abbreviations: HLA, human leukocyte antigen

[a] To explore multiplicative interactions between each HLA allele and lifestyle on incident cancer risk for 16 cancer outcomes for men, we conducted the multiplicative interaction analyses in the multivariable-adjusted Cox regression model by incorporating an interaction term represented as cross-product of each HLA allele's binary carrier status and the three-level lifestyle group (ideal, intermediate and poor lifestyles), with P value of the interaction term reported based on Wald test.

* For each the 16 cancer outcomes for men, a significance level of $P<0.001$ (0.05/50) was considered statistical significance after applying Bonferroni correction. The statistically significant P values were shown in bold

Supplementary Table S17. P values of multiplicative interactions between HLA allele carrier status and lifestyle on the incident risk of all cancer outcomes among women [a]

HLA allele	Breast cancer	Colorectal cancer	Lung cancer	Skin cancer	Endometrial cancer	Ovarian cancer	non-Hodgkin lymphoma	Pancreatic cancer	Kidney cancer	Leukemia	Other cancers	Overall cancer
A*01:01	0.1310	0.3437	0.5916	0.0694	0.1051	0.0832	0.7461	0.0034	0.6747	0.4006	**0.0005***	0.1883
A*02:01	0.4684	0.8032	0.4257	0.3670	0.1235	0.4628	0.8665	0.1027	0.6930	0.7103	0.9031	0.4600
A*03:01	0.3580	0.6509	0.6943	0.2389	0.6377	0.6024	0.8956	0.7361	0.1703	0.3046	0.6225	0.7042
A*11:01	0.8564	0.3197	0.2980	0.3601	0.2005	0.9465	0.3003	0.8520	0.7200	0.5778	0.2577	0.6652
A*24:02	0.0598	0.2428	0.9631	0.7512	0.2711	0.1497	0.8937	0.6605	0.0516	0.8029	0.8212	0.2173
B*07:02	0.1343	0.2247	0.0461	0.6843	0.6995	0.9240	0.2747	0.9304	0.7391	0.6539	0.7429	0.4012
B*08:01	0.8365	0.7512	0.9496	0.5978	0.4063	0.4413	0.6458	0.2427	0.9269	0.3544	0.0673	0.6575
B*15:01	0.4653	0.0935	0.7951	0.5752	0.7525	0.4900	0.9972	0.3105	0.5840	0.6445	0.5537	0.2383
B*40:01	0.0379	0.6183	0.6226	0.3549	0.9781	0.2541	0.1121	0.4834	0.5695	0.5633	0.2018	0.0498
B*44:02	0.6819	0.8647	0.7878	0.1669	0.0736	0.4364	0.9579	0.7911	0.9435	0.3130	0.2605	0.2610
B*44:03	0.8114	0.2901	0.6519	0.3931	0.9838	0.2651	0.4380	0.2208	0.2119	0.2649	0.7943	0.8131
C*03:03	0.0292	0.6687	0.7195	0.7799	0.7683	0.3518	0.4533	0.3315	0.6374	0.3654	0.3066	0.3584
C*03:04	0.0327	0.8848	0.3676	0.3518	0.6095	0.5916	0.1043	0.6461	0.4314	0.5802	0.4715	0.0974
C*04:01	0.4370	0.8252	0.8289	0.3251	0.6693	0.9725	0.7850	0.4237	0.4307	0.6031	0.8455	0.2427
C*05:01	0.5628	0.3572	0.5382	0.4936	0.0841	0.8179	0.3236	0.9378	0.6516	0.8585	0.3045	0.1528
C*06:02	0.6271	0.5316	0.5544	0.7478	0.1547	0.3624	0.2300	0.3915	0.8006	0.3598	0.7442	0.0482
C*07:01	0.4323	0.7989	0.9626	0.2513	0.1163	0.3428	0.1666	0.3987	0.8510	0.4388	0.0728	0.2046
C*07:02	0.0923	0.1809	0.0680	0.6339	0.8469	0.8820	0.5426	0.9600	0.8851	0.5553	0.6513	0.2735
DPA1*01:03	0.3479	0.6202	0.6504	0.7141	0.7522	0.1777	0.6848	0.7406	0.9974	0.4163	0.8044	0.9119
DPA1*02:01	0.1618	0.9004	0.7298	0.2096	0.3353	0.4120	0.4944	0.4648	0.8566	0.9352	0.1835	0.0569
DPB1*01:01	0.2901	0.5252	0.2069	0.4228	0.6200	0.6567	0.4613	0.3651	0.4605	0.3351	0.5920	0.6292
DPB1*02:01	0.3742	0.2344	0.0742	0.3770	0.4569	0.4080	0.3576	0.2966	0.0598	0.2274	0.5276	0.2223
DPB1*03:01	0.4358	0.0998	0.4008	0.4730	0.1594	0.0746	0.4972	0.8914	0.6713	0.7214	0.8040	0.4969
DPB1*04:01	0.0431	0.5290	0.4539	0.3072	0.5752	0.0144	0.0190	0.8647	0.3542	0.3616	0.8771	0.0122
DPB1*04:02	0.8189	0.1287	0.5503	0.2190	0.2920	0.9351	0.7071	0.7851	0.8958	0.1981	0.6544	0.5833
DQA1*01:01	0.0163	0.7258	0.7763	0.9126	0.5649	0.8709	0.3863	0.1811	0.5384	0.7101	0.1788	0.1693
DQA1*01:02	0.7846	0.6014	0.9628	0.2114	0.7103	0.4472	0.4349	0.2764	0.3846	0.0896	0.7353	0.6993
DQA1*01:03	0.8812	0.4489	0.2761	0.2853	0.5213	0.6023	0.8031	0.1821	0.6449	0.4671	0.1964	0.2857
DQA1*02:01	0.7985	0.4413	0.2787	0.7770	0.3759	0.1258	0.6696	0.8429	0.4781	0.2980	0.8042	0.7732
DQA1*03:01	0.9520	0.8035	0.0928	0.7693	0.8667	0.7769	0.6409	0.5683	0.7397	0.3791	0.2337	0.4606
DQA1*05:01	0.9960	0.8876	0.1936	0.4766	0.4134	0.3885	0.5107	0.2658	0.5029	0.6048	0.1077	0.7535
DQB1*02:01	0.9453	0.6824	0.9046	0.5029	0.7635	0.5369	0.3421	0.1566	0.7698	0.5765	0.1178	0.4400

HLA allele	Breast cancer	Colorectal cancer	Lung cancer	Skin cancer	Endometrial cancer	Ovarian cancer	non-Hodgkin lymphoma	Pancreatic cancer	Kidney cancer	Leukemia	Other cancers	Overall cancer
DQB1*02:02	0.5228	0.9597	0.8865	0.6710	0.9300	0.2399	0.5616	0.9021	0.7383	0.4874	0.6726	0.8101
DQB1*03:01	0.1928	0.4318	0.2432	0.8368	0.0858	0.3157	0.8146	0.0852	0.9453	0.1563	0.0982	0.2752
DQB1*03:02	0.6047	0.0139	0.0331	0.2560	0.8629	0.2990	0.5646	0.7478	0.1531	0.4944	0.6506	0.4513
DQB1*03:03	0.5177	0.5652	0.6709	0.1246	0.0730	0.1581	0.9231	0.8345	0.3228	0.5944	0.5750	0.4550
DQB1*05:01	0.0036	0.7977	0.5684	0.9652	0.5543	0.5701	0.3959	0.6557	0.7578	0.7839	0.1757	0.0646
DQB1*06:02	0.4874	0.5071	0.8959	0.3941	0.4663	0.4226	0.1643	0.5919	0.0943	0.1469	0.4332	0.7758
DQB1*06:03	0.9284	0.8134	0.2548	0.3706	0.4002	0.6861	0.7660	0.2137	0.6816	0.1321	0.0893	0.3589
DRB1*01:01	0.0156	0.9137	0.5648	0.5118	0.5879	0.6659	0.2315	0.3300	0.7821	0.5712	0.7712	0.1492
DRB1*03:01	0.9356	0.8780	0.9014	0.5163	0.7381	0.6601	0.4432	0.1560	0.4496	0.6180	0.0842	0.3879
DRB1*04:01	0.2937	0.5490	0.3613	0.1332	0.6579	0.9311	0.8444	0.7385	0.1916	0.8922	0.2634	0.7120
DRB1*07:01	0.8515	0.4934	0.2499	0.7336	0.3953	0.1249	0.7427	0.8461	0.3677	0.2922	0.7922	0.7687
DRB1*13:01	0.9611	0.7941	0.2353	0.3658	0.6077	0.6935	0.5941	0.1880	0.7188	0.3465	0.1850	0.4910
DRB1*15:01	0.3993	0.6156	0.6695	0.4607	0.6579	0.4702	0.1624	0.3853	0.2220	0.3824	0.5349	0.7841
DRB3*01:01	0.4671	0.8812	0.6964	0.9728	0.9303	0.6169	0.6306	0.0278	0.9767	0.3150	0.5211	0.2123
DRB3*02:02	0.2497	0.1563	0.0548	0.6961	0.2331	0.9814	0.9316	0.2027	0.8381	0.6035	0.8673	0.5839
DRB4*01:01	0.6647	0.9221	0.6469	0.1521	0.9658	0.4108	0.6271	0.5664	0.7430	0.2853	0.6295	0.6344
DRB4*01:03	0.5595	0.7825	0.1545	0.4691	0.3628	0.3284	0.9006	0.5643	0.5751	0.2967	0.4727	0.1184
DRB5*01:01	0.3741	0.6524	0.7872	0.5480	0.5719	0.4422	0.1088	0.3795	0.2234	0.1904	0.4678	0.7845

Abbreviations: HLA, human leukocyte antigen

[a] To explore multiplicative interactions between each HLA allele and lifestyle on incident cancer risk for 12 cancer outcomes for women, we conducted the multiplicative interaction analyses in the multivariable-adjusted Cox regression model by incorporating an interaction term represented as cross-product of each HLA allele's binary carrier status and the three-level lifestyle group (ideal, intermediate and poor lifestyles), with P value of the interaction term reported based on Wald test.

* For each the 12 cancer outcomes for women, a significance level of P<0.001 (0.05/50) was considered statistical significance after applying Bonferroni correction. The statistically significant P values were shown in bold

Supplementary Table S18. *P* values of additive interactions between HLA allele carrier status and lifestyle on the incident risk of all cancer outcomes among men [a]

HLA allele	Prostate cancer	Colorectal cancer	Lung cancer	Skin cancer	non-Hodgkin lymphoma	Kidney cancer	Bladder cancer	Esophageal cancer	Leukemia	Pancreatic cancer	Gastric cancer	Brain cancer	Multiple myeloma	Liver cancer	Other cancers	Overall cancer
**A*01:01**	0.5202	0.5223	0.0528	0.9406	0.5463	0.7404	0.3789	0.6180	0.4211	0.4778	0.5289	0.0030	0.0764	0.6283	0.2735	0.9438
**A*02:01**	0.0011	0.7095	0.0552	0.4739	0.0026	**0.0002***	0.7787	0.6903	0.7373	0.1088	0.5750	0.8108	0.1230	0.5004	0.3538	0.1704
**A*03:01**	0.6342	0.8459	0.3974	0.0647	**0.0001***	0.1269	0.1130	0.6480	0.0494	0.2504	0.1585	0.3511	0.0010	0.6110	0.2773	0.6062
**A*11:01**	0.8167	0.0261	0.4513	0.3599	0.7680	0.0094	0.0092	0.0599	0.3952	0.4874	0.5975	0.9454	0.7077	0.5003	0.6801	0.2086
**A*24:02**	0.9694	0.0332	0.1230	0.7287	0.7610	0.3714	0.1475	0.1130	0.4304	0.3540	0.2579	0.0357	0.0019	0.2216	0.6881	0.8713
**B*07:02**	0.4175	0.2048	0.5853	0.4840	**2.40E-05***	0.3477	0.3344	0.6473	0.1249	0.3881	0.2218	0.4035	0.8800	0.0907	0.0674	0.7190
**B*08:01**	0.8408	0.3105	**0.0005***	**0.0002***	0.0327	0.0193	0.7136	0.2852	0.1825	0.4845	0.0580	0.8014	0.5058	0.3937	0.2477	**1.40E-05***
**B*15:01**	0.2784	0.5652	0.1738	0.1255	0.1280	0.5850	0.5522	0.1189	0.3809	0.2351	0.4376	0.5004	0.5920	0.3321	0.6390	0.3812
**B*40:01**	0.9831	0.5397	0.4990	0.8906	0.7933	0.3290	0.4293	0.1263	0.8550	0.5004	0.8669	0.8842	0.5003	0.4032	0.0073	0.9985
**B*44:02**	0.0281	0.4241	0.3286	0.3438	0.5302	0.8814	0.5353	0.4509	0.6738	0.1091	0.0031	0.0631	0.1466	0.0808	0.5720	0.1550
**B*44:03**	0.4077	0.0674	0.1994	0.4489	0.4984	0.2252	0.7930	0.4922	0.7433	0.1603	0.6114	0.2405	0.5004	0.4997	0.2258	0.6285
**C*03:03**	0.5349	0.6138	0.1804	0.0828	0.7341	0.2437	0.2398	0.4521	0.6728	0.0174	0.1398	0.6851	0.7076	0.5204	0.6185	0.4168
**C*03:04**	0.8846	0.4948	0.0868	0.8555	0.0127	0.3134	0.8713	0.0726	0.7279	0.5834	0.0545	0.7328	0.5004	0.4996	0.0109	0.9881
**C*04:01**	0.1001	0.3772	0.1054	0.5837	0.5985	0.0181	0.6682	0.4910	0.0207	0.0081	0.5403	0.7085	0.1994	0.7201	0.2337	0.3307
**C*05:01**	0.7907	0.7703	0.7080	0.8195	0.2302	0.0018	0.2081	0.2197	0.7869	0.7956	0.0597	0.9079	0.5514	0.0119	0.6064	0.5692
**C*06:02**	0.0415	0.9614	0.7045	0.0811	0.8863	0.1517	0.6498	0.1828	0.6148	0.5183	0.4865	0.7843	0.5004	0.0837	0.5502	0.2933
**C*07:01**	0.7926	0.4352	**0.0002***	**0.0006***	0.0199	0.7077	0.8162	0.4951	0.2525	0.3306	0.0771	0.0518	0.8697	0.5928	0.3267	**0.0004***
**C*07:02**	0.3542	0.2338	0.4109	0.4224	**0.0001***	0.2234	0.2142	0.5860	0.3104	0.3088	0.2232	0.4650	0.8677	0.1624	0.1097	0.6858
**DPA1*01:03**	0.7061	0.7452	0.5117	0.1311	0.7252	0.4995	0.1115	0.2972	0.4996	0.0527	0.2435	0.0705	0.5002	0.2500	0.2787	0.2721
**DPA1*02:01**	0.6076	0.0270	0.6089	0.3381	0.4210	0.6776	0.5222	0.1055	0.1838	0.7708	0.7163	0.2686	0.0505	0.5005	0.4367	0.5024
**DPB1*01:01**	0.3616	0.9049	0.1819	0.0857	0.4092	0.5616	0.2430	0.0160	0.0236	0.3762	0.5004	0.2403	0.6635	0.2269	0.3082	0.2597
**DPB1*02:01**	0.4199	0.3730	0.0533	0.0668	0.5283	0.0553	0.4652	0.6312	0.4077	0.5890	0.5836	0.0016	0.0063	0.6766	0.2842	0.5039
**DPB1*03:01**	0.9636	0.5662	0.2951	0.2233	0.8847	0.8532	0.2715	0.4086	0.4855	0.0638	0.7110	0.2217	0.6020	0.3241	0.4734	0.7981
**DPB1*04:01**	0.7093	0.0300	0.0987	0.2550	0.8152	0.7982	0.3240	0.5105	0.4154	0.7780	0.5190	0.2214	0.6685	0.3764	0.2500	0.4183
**DPB1*04:02**	0.8482	0.7002	0.4774	0.7662	0.6231	0.1761	0.1230	0.0054	0.3047	0.7819	0.3160	0.2046	0.8416	0.5004	0.8049	0.8116
**DQA1*01:01**	0.7212	0.7327	0.0020	0.5226	0.2221	0.0138	0.2089	0.2633	0.0209	0.8788	0.2635	0.0736	0.7459	0.5005	0.5713	0.6304
**DQA1*01:02**	0.2067	0.0159	0.3007	0.6278	0.1469	0.8645	0.1145	0.4318	0.8817	0.1677	0.6460	0.1942	0.6909	0.5472	0.7212	0.4569
**DQA1*01:03**	0.2197	0.5860	0.0481	**1.70E-05***	0.2342	0.0101	0.7104	0.0183	0.3257	0.0049	0.4208	0.8696	0.0757	0.6851	0.7058	0.2691
**DQA1*02:01**	0.7232	0.1183	0.1875	0.7414	0.6930	0.0030	0.0131	0.2170	0.4059	0.7646	0.4585	0.0893	0.5003	0.3871	0.2612	0.7508
**DQA1*03:01**	0.8859	0.8051	0.2423	0.0440	0.5586	0.7609	0.0034	0.4092	0.2578	0.1410	0.3926	0.0026	0.5034	0.2970	0.8963	0.9946
**DQA1*05:01**	0.8473	0.6608	**3.90E-05***	0.0060	0.3332	0.0773	0.3108	0.0462	0.4434	0.2079	0.7330	0.0721	0.8548	0.7655	0.5767	0.0045

HLA allele	Prostate cancer	Colorectal cancer	Lung cancer	Skin cancer	non-Hodgkin lymphoma	Kidney cancer	Bladder cancer	Esophageal cancer	Leukemia	Pancreatic cancer	Gastric cancer	Brain cancer	Multiple myeloma	Liver cancer	Other cancers	Overall cancer
**DQB1*02:01**	0.7815	0.8054	**0.0003***	0.0929	0.2158	0.0210	0.4417	0.0210	0.2412	0.6363	0.5424	0.8127	0.5897	0.4732	0.6219	0.0060
**DQB1*02:02**	0.4543	0.1021	0.3409	0.4932	0.3296	0.0366	0.0452	0.2763	0.6429	0.6842	0.6699	0.1029	0.5003	0.2206	0.5372	0.4167
**DQB1*03:01**	0.3562	0.5289	0.3318	0.7587	0.2454	0.7458	0.6081	0.1966	0.4928	0.0074	0.6085	0.1573	**0.0001***	0.5070	0.5062	0.5059
**DQB1*03:02**	0.8919	0.8498	0.0464	0.0041	0.7630	0.2967	0.9605	0.6872	0.2316	0.7363	0.0633	0.4836	0.5682	0.5004	0.9133	0.9978
**DQB1*03:03**	0.1573	0.7803	0.7371	0.0330	0.4508	0.0226	0.3880	0.3269	0.5251	0.1348	0.0318	0.0080	0.5003	0.5509	0.7428	0.1749
**DQB1*05:01**	0.8289	0.7169	**0.0002***	0.5220	0.4109	0.0774	0.5477	0.3853	0.0637	0.0017	0.7349	0.0228	0.5398	0.5004	0.5679	0.5200
**DQB1*06:02**	0.7440	0.9231	0.4662	0.5117	0.1342	0.7316	0.3677	0.2038	0.8708	0.1605	0.2236	0.2756	0.5264	0.0944	0.5533	0.4915
**DQB1*06:03**	0.0610	0.4863	0.0168	**0.0001***	0.1506	0.0138	0.6866	0.0615	0.3535	**0.0004***	0.5003	0.8886	0.0986	0.6261	0.7538	0.4626
**DRB1*01:01**	0.8820	0.7340	0.0041	0.5896	0.2078	0.0489	0.5536	0.1009	0.1346	0.0018	0.1036	0.0512	0.7639	0.5004	0.3716	0.7696
**DRB1*03:01**	0.7504	0.6805	**0.0003***	0.0376	0.2171	0.8818	0.3173	0.0121	0.1381	0.5928	0.5430	0.7996	0.4032	0.4698	0.6194	0.0014
**DRB1*04:01**	0.6210	0.5605	0.1986	0.5611	0.3232	0.2746	0.2181	0.1610	0.2745	0.5584	0.0373	0.0722	0.2489	0.0599	0.5942	0.6046
**DRB1*07:01**	0.6646	0.1397	0.1359	0.7584	0.6664	**0.0004***	0.0163	0.2429	0.3652	0.7655	0.4536	0.0902	0.5003	0.3335	0.2870	0.6934
**DRB1*13:01**	0.1257	0.5023	0.0104	**0.0001***	0.0999	0.0260	0.6909	**0.0003***	0.3514	0.0023	0.5003	0.8915	0.0990	0.6144	0.7202	0.2615
**DRB1*15:01**	0.7303	0.1172	0.3792	0.6061	0.1624	0.7398	0.2642	0.1582	0.8653	0.1201	0.2148	0.2707	0.7209	0.1506	0.5994	0.3541
**DRB3*01:01**	0.8655	0.6829	0.0161	0.5391	0.3067	**0.0006***	0.7007	0.5610	0.3571	0.2557	0.6210	0.6819	0.2620	0.1507	0.4260	0.0190
**DRB3*02:02**	0.3370	0.3477	0.0100	0.3575	0.5097	0.2677	0.5877	0.5447	0.7654	0.2709	0.2656	0.7689	0.9451	0.3015	0.6826	0.3505
**DRB4*01:01**	0.3877	0.1046	0.7050	0.0958	0.3830	**0.0001***	0.1982	0.2988	0.2414	0.0133	0.6374	0.3064	0.0013	0.2800	0.2358	0.3548
**DRB4*01:03**	0.4499	0.7494	0.0767	0.6557	0.6187	0.5553	0.8582	0.6053	0.1201	0.3844	0.5960	0.0013	0.8287	0.2074	0.9397	0.8217
**DRB5*01:01**	0.6960	0.1003	0.3991	0.5794	0.0938	0.6690	0.2359	0.1768	0.8664	0.0934	0.2216	0.2682	0.7168	0.1562	0.6264	0.3997

Abbreviations: HLA, human leukocyte antigen

[a] The additive interaction analysis was performed with the relative excess risk due to interaction with the 95%CIs estimated by delta method. In the additive interaction, the referent for each lifestyle was the favorable lifestyle group and the referent for each binary HLA allele carrier status was the allele carrier status that was associated with decreased cancer risk based on the results from the independent effects of each HLA allele on cancer outcomes.

* $P<0.001$ (0.05/50) was considered statistical significance after Bonferroni correction. The statistically significant P values were shown in bold.

.

Supplementary Table S19. P values of additive interactions between HLA allele carrier status and lifestyle on the incident risk of all cancer outcomes among women [a]

HLA allele	Breast cancer	Colorectal cancer	Lung cancer	Skin cancer	Endometrial cancer	Ovarian cancer	Non-Hodgkin's lymphoma	Pancreatic cancer	Kidney cancer	Leukemia	Other cancers	Overall cancer
**A*01:01**	0.0167	0.8096	0.5583	0.0064	0.0870	0.1950	0.6394	0.0245	0.1227	0.8129	**0.0001***	0.0283
**A*02:01**	0.4516	0.6665	0.1007	0.9058	0.9421	0.0776	0.2722	0.0087	0.3744	0.3852	0.6654	0.4473
**A*03:01**	0.0477	0.3574	0.3806	0.0379	0.4753	0.5602	0.3922	0.1645	0.6667	0.8914	0.2469	0.1914
**A*11:01**	0.3837	0.0965	0.7632	0.6879	0.0050	0.5397	0.0401	0.5567	0.1844	0.7538	0.9018	0.1667
**A*24:02**	0.2896	0.7738	0.4110	0.7130	0.0820	0.0091	0.6484	0.6174	**0.0009***	0.6782	0.3726	0.6759
**B*07:02**	0.0162	0.8845	0.0125	0.4412	0.0993	0.4697	0.0452	0.4515	0.1932	0.7848	0.5261	0.8050
**B*08:01**	0.4820	0.4945	0.3332	0.4790	0.7558	0.8519	0.7543	0.7327	0.2395	0.7627	0.0060	0.1451
**B*15:01**	0.8231	0.0893	0.4456	0.7600	0.6186	0.0685	0.4903	0.7827	0.5005	0.6665	0.0952	0.9022
**B*40:01**	0.9793	0.2247	0.1654	0.8574	0.4778	0.0306	0.8243	0.0838	0.0650	0.6310	0.3588	0.0058
**B*44:02**	0.5705	0.6780	0.4927	0.6153	0.4721	0.0932	0.5464	0.5143	0.4375	0.8068	0.9369	0.2310
**B*44:03**	0.6238	0.1182	0.4963	0.6803	0.4509	0.8061	0.8264	0.7797	0.0042	0.7727	0.5708	0.7060
**C*03:03**	0.4343	0.2182	0.6789	0.7531	0.6258	0.0748	0.7691	0.1420	0.4365	0.7590	0.0655	0.5443
**C*03:04**	0.9862	0.3120	0.1192	0.7828	0.4598	0.2346	0.8703	0.7567	0.0390	0.7370	0.7468	0.0153
**C*04:01**	0.7257	0.5240	0.5012	0.8650	0.7651	0.5376	0.2253	0.5765	0.0019	0.0916	0.6737	0.8655
**C*05:01**	0.2377	0.8893	0.5568	0.5199	0.3929	0.3756	0.0280	0.5892	0.4578	0.5007	0.0636	0.0197
**C*06:02**	0.5546	0.8199	0.1100	0.3399	0.3639	0.8263	0.8206	0.0983	0.1969	0.2289	0.6989	0.8392
**C*07:01**	0.7683	0.4981	0.2684	0.8783	0.8544	0.8684	0.9303	0.7792	0.1274	0.5368	0.0066	0.0300
**C*07:02**	0.0085	0.8934	0.9467	0.5803	0.1528	0.4843	0.1275	0.4732	0.6174	0.8238	0.6746	0.9072
**DPA1*01:03**	0.6268	0.6791	0.6323	0.7228	0.7023	0.7554	0.7878	0.5004	0.4996	0.7490	0.6934	0.5627
**DPA1*02:01**	0.3521	0.5930	0.2196	0.0304	0.8968	0.5560	0.2215	0.1089	0.4307	0.5425	0.7012	0.7882
**DPB1*01:01**	0.3144	0.2154	0.0168	0.3171	0.1940	0.7109	0.7039	0.1531	0.2100	0.4750	0.4160	0.7028
**DPB1*02:01**	0.3995	0.9065	0.8586	0.8716	0.0811	0.0287	0.0978	0.0240	0.0244	0.7729	0.1352	0.1076
**DPB1*03:01**	0.1544	0.0827	0.6548	0.1940	0.0065	0.0479	0.7329	0.4392	0.1310	0.2515	0.2299	0.1089
**DPB1*04:01**	0.9856	0.1220	0.0717	0.5715	0.7962	0.7558	0.0906	0.3492	0.1078	0.8398	0.6686	0.9968
**DPB1*04:02**	0.6109	0.9424	0.0483	0.8240	0.9072	0.4868	0.3782	0.6877	0.4314	0.2453	0.5898	0.5378
**DQA1*01:01**	0.2552	0.5909	0.1137	0.4140	0.2615	0.6026	0.8732	0.8548	0.1153	0.6784	0.0175	0.1281
**DQA1*01:02**	0.7019	0.3741	0.3632	0.1419	0.2130	0.1555	0.7927	0.3792	0.6839	0.0673	0.6364	0.4385
**DQA1*01:03**	0.4742	0.8220	0.8078	0.0297	0.7888	0.5925	0.6448	0.2362	0.6222	0.0812	0.9187	0.7519
**DQA1*02:01**	0.5411	0.8708	0.8296	0.4533	0.3545	0.9487	0.5426	0.6377	0.5251	0.3318	0.4827	0.7452
**DQA1*03:01**	0.3842	0.7254	0.0138	0.2383	0.1428	0.6329	0.7647	0.7741	0.4501	0.0996	0.8844	0.2029
**DQA1*05:01**	0.5246	0.3887	0.0857	0.8729	0.7729	0.8678	0.8218	0.8813	0.0281	0.7474	0.9678	0.7037
**DQB1*02:01**	0.3891	0.7594	0.3258	0.1845	0.6133	0.0988	0.8910	0.8540	0.3038	0.7540	0.9503	0.3950
**DQB1*02:02**	0.5365	0.5842	0.5681	0.7171	0.3830	0.8622	0.7663	0.4747	0.6135	0.4135	0.5524	0.5189
**DQB1*03:01**	0.8290	0.1303	0.7369	0.3274	0.3515	0.1410	0.6163	0.5787	0.5247	0.8959	0.0397	0.4706

**DQB1*03:02**	0.4083	0.9484	0.0212	0.4976	0.5091	0.0377	0.7836	0.3269	0.5866	0.8490	0.4330	0.6400
**DQB1*03:03**	0.5648	0.8441	0.1971	0.0042	0.1315	0.8366	0.6359	0.6588	0.8146	0.1989	0.7958	0.2163
**DQB1*05:01**	0.2024	0.2808	0.0286	0.4465	0.3590	0.7494	0.7698	0.7011	0.1874	0.7078	0.0180	0.1227
**DQB1*06:02**	0.1312	0.7887	0.3726	0.1570	0.2127	0.5919	0.7462	0.2155	0.7956	0.0263	0.1450	0.4592
**DQB1*06:03**	0.4843	0.6994	0.7734	0.0481	0.8514	0.4070	0.6425	0.1918	0.5719	0.0340	0.0060	0.1773
**DRB1*01:01**	0.1808	0.4298	0.1797	0.1551	0.5294	0.7339	0.8073	0.7161	0.2724	0.7371	0.2162	0.2512
**DRB1*03:01**	0.4831	0.6541	0.3469	0.1549	0.5839	0.1493	0.8606	0.8531	0.1960	0.7555	0.9634	0.4203
**DRB1*04:01**	0.8982	0.3126	0.3000	0.9428	0.6193	0.5921	0.4306	0.7154	0.0958	0.5927	0.0333	0.7140
**DRB1*07:01**	0.5518	0.8670	0.8470	0.4499	0.3188	0.9487	0.5910	0.6375	0.4618	0.3300	0.4280	0.7309
**DRB1*13:01**	0.3684	0.6921	0.7850	0.0472	0.7008	0.4859	0.6481	0.2329	0.5801	0.7847	0.0364	0.6434
**DRB1*15:01**	0.0803	0.7429	0.4045	0.1336	0.3241	0.3780	0.1990	0.1496	0.7465	0.2245	0.7902	0.4965
**DRB3*01:01**	0.1797	0.6427	0.2561	0.4590	0.6216	0.2945	0.7881	0.8686	0.3158	0.6865	0.8227	0.2611
**DRB3*02:02**	0.5084	0.0144	0.8910	0.7378	0.5255	0.5277	0.3518	0.2271	0.1404	0.7553	0.5681	0.4117
**DRB4*01:01**	0.4029	0.6368	0.3563	0.7793	0.3861	0.8498	0.7855	0.7083	0.5933	0.6157	0.6314	0.4516
**DRB4*01:03**	0.1448	0.2210	0.9161	0.7051	0.0613	0.8911	0.3444	0.4412	0.7444	0.0311	0.8461	0.0126
**DRB5*01:01**	0.0722	0.7047	0.3798	0.1782	0.3923	0.4264	0.1676	0.1502	0.7457	0.1025	0.8150	0.5667

Abbreviations: HLA, human leukocyte antigen

[a] The additive interaction analysis was performed with the relative excess risk due to interaction with the 95%CIs estimated by delta method. In the additive interaction, the referent for each lifestyle was the favorable lifestyle group and the referent for each binary HLA allele carrier status was the allele carrier status that was associated with decreased cancer risk based on the results from the independent effects of each HLA allele on cancer outcomes.

* P<0.001 (0.05/50) was considered statistical significance after Bonferroni correction. The statistically significant P values were shown in bold.

Supplementary Table S20. Joint effects of binary HLA allele carrier status and individual lifestyle factors on the incident risk of specific cancers among men and women based on the identified significant additive interactions

Cancer types [a]	Subgroup [b]	Smoking		Alcohol		Physical activity		Weight		Diet	
		Cases No. / Person-years	Multivariable-adjusted HR (95%CI) [c]	Cases No. / Person-years	Multivariable-adjusted HR (95%CI) [c]	Cases No. / Person-years	Multivariable-adjusted HR (95%CI) [c]	Cases No. / Person-years	Multivariable-adjusted HR (95%CI) [c]	Cases No. / Person-years	Multivariable-adjusted HR (95%CI) [c]
Men											
**Lung cancer**	**B*08:01 non-carrier**										
	Favorable	140 / 770683	Ref	15 / 22022	Ref	431 / 724888	Ref	721 / 1086469	Ref	129 / 289076	Ref
	Unfavorable	765 / 455184	7.84 (6.53-9.42)	927 / 1343887	0.76 (0.46-1.27)	324 / 443481	1.08 (0.93-1.24)	232 / 287573	0.84 (0.72-0.97)	830 / 1089403	1.44 (1.20-1.74)
	**B*08:01 carrier**										
	Favorable	42 / 284121	0.81 (0.58-1.15)	3 / 8868	0.50 (0.15-1.74)	170 / 265716	1.08 (0.90-1.29)	311 / 396014	1.18 (1.04-1.35)	56 / 105376	1.19 (0.87-1.63)
	Unfavorable	349 / 165766	9.87 (8.09-12.03)	408 / 493364	0.92 (0.55-1.54)	171 / 165100	1.57 (1.31-1.87)	102 / 108815	1.03 (0.84-1.27)	362 / 401106	1.73 (1.41-2.12)
**RERI (95%CI) [d]**		2.23 (0.99-3.48)		0.76 (0.17-1.35)		0.45 (0.12-0.78)		0.02 (-0.29-0.33)		0.14 (-0.28-0.56)	
***P* value [d]**		**<0.001**		**0.006**		**0.004**		0.45		0.26	
**Lung cancer**	**DQA1*05:01 non-carrier**										
	Favorable	119 / 624452	Ref	11 / 17852	Ref	336 / 585977	Ref	565 / 879054	Ref	111 / 233750	Ref
	Unfavorable	586 / 367772	7.10 (5.81-8.66)	722 / 1087916	0.77 (0.42-1.40)	258 / 358657	1.10 (0.93-1.29)	175 / 233160	0.82 (0.70-0.98)	635 / 882102	1.27 (1.04-1.56)
	**DQA1*05:01 carrier**										
	Favorable	63 / 430353	0.78 (0.57-1.05)	7 / 13039	0.79 (0.31-2.04)	265 / 404627	1.14 (0.97-1.34)	467 / 603429	1.22 (1.08-1.38)	74 / 160702	0.96 (0.71-1.29)
	Unfavorable	528 / 253178	9.39 (7.68-11.48)	613 / 749335	0.96 (0.53-1.75)	237 / 249924	1.48 (1.25-1.75)	159 / 163227	1.07 (0.89-1.27)	557 / 608407	1.64 (1.34-2.01)
**RERI (95%CI) [d]**		2.51 (1.45-3.57)		0.51 (-0.17-1.19)		0.25 (-0.05-0.55)		0.09 (-0.19-0.38)		0.48 (0.17-0.80)	
***P* value [d]**		**<0.001**		0.07		0.05		0.26		**0.001**	
**Lung cancer**	**DQB1*02:01 non-carrier**										
	Favorable	136 / 764688	Ref	15 / 21676	Ref	413 / 717461	Ref	691 / 1076404	Ref	130 / 286617	Ref
	Unfavorable	737 / 449660	7.84 (6.51-9.44)	893 / 1332276	0.73 (0.44-1.21)	315 / 439484	1.09 (0.94-1.26)	225 / 285604	0.86 (0.74-1.00)	792 / 1079789	1.37 (1.14-1.65)
	**DQB1*02:01 carrier**										
	Favorable	46 / 290116	0.90 (0.64-1.25)	3 / 9215	0.47 (0.14-1.62)	188 / 273142	1.18 (0.99-1.41)	341 / 406080	1.30 (1.14-1.48)	55 / 107835	1.11 (0.81-1.52)
	Unfavorable	377 / 171290	10.50 (8.61-12.81)	442 / 504975	0.94 (0.56-1.58)	180 / 169096	1.64 (1.37-1.95)	109 / 110784	1.08 (0.88-1.32)	455 / 410720	1.81 (1.48-2.21)
**RERI (95%CI) [d]**		2.78 (1.49-4.07)		0.91 (0.38-1.44)		0.40 (0.06-0.74)		-0.04 (-0.37-0.28)		0.42 (0.02-0.81)	
***P* value [d]**		**<0.001**		**<0.001**		**0.01**		0.60		**0.02**	
**Lung cancer**	**DRB1*03:01 non-carrier**										
	Favorable lifestyle	137 / 766051	Ref	15 / 21702	Ref	415 / 718874	Ref	693 / 1078048	Ref	131 / 286934	Ref
	Poor lifestyle	739 / 450068	7.80 (6.49-9.39)	895 / 1334374	0.73 (0.44-1.22)	315 / 440001	1.09 (0.94-1.26)	225 / 286066	0.85 (0.73-0.99)	793 / 1081580	1.36 (1.13-1.64)
	**DRB1*03:01 carrier**										
	Favorable lifestyle	45 / 288753	0.87 (0.62-1.22)	3 / 9189	0.47 (0.14-1.63)	186 / 271729	1.17 (0.98-1.39)	339 / 404435	1.29 (1.14-1.47)	54 / 107518	1.09 (0.79-1.49)
	Poor lifestyle	375 / 170882	10.40 (8.53-12.68)	440 / 502877	0.94 (0.56-1.58)	180 / 168580	1.63 (1.37-1.95)	109 / 110321	1.08 (0.88-1.33)	399 / 408929	1.80 (1.47-2.19)
**RERI (95%CI) [d]**		2.74 (1.47-4.02)		0.90 (0.37-1.44)		0.41 (0.07-0.75)		-0.03 (-0.36-0.29)		0.44 (0.05-0.83)	
***P* value [d]**		**<0.001**		**<0.001**		**0.009**		0.58		**0.01**	
Women											
**Kidney cancer**	**A*24:02 non-carrier**										
	Favorable lifestyle	143 / 1168169	Ref	11 / 77332	Ref	108 / 851214	Ref	178 / 1436880	Ref	80 / 634228	Ref
	Poor lifestyle	78 / 438538	1.43 (1.08-1.89)	231 / 1712736	1.11 (0.61-2.05)	80 / 577156	1.07 (0.79-1.43)	78 / 388905	1.43 (1.09-1.88)	176 / 1196674	1.16 (0.89-1.52)
	**A*24:02 carrier**										
	Favorable lifestyle	39 / 194973	1.66 (1.16-2.36)	0 / 12823	NA	13 / 140336	0.74 (0.41-1.31)	48 / 239273	1.62 (1.18-2.23)	24 / 106448	1.80 (1.14-2.84)
	Poor lifestyle	28 / 71568	3.14 (2.09-4.73)	71 / 283687	2.08 (1.10-3.94)	34 / 94834	2.78 (1.89-4.11)	24 / 62989	2.77 (1.80-4.25)	48 / 196715	1.94 (1.36-2.79)
**RERI (95%CI) [d]**		1.14 (-0.22-2.51)		1.99 (1.21-2.77)		2.10 (0.98-3.22)		0.81 (-0.51-2.13)		0.06 (-0.91-1.03)	
***P* value [d]**		0.05		**<0.001**		**<0.001**		0.11		0.45	

Abbreviations: CI, confidence interval; HLI, Healthy Lifestyle Index; HR, hazard ratio; RERI, relative excess risk due to interaction.

[a] The selection of cancer types and corresponding HLA alleles for men and women separately in this table was based on three criteria: 1) the additive interaction of binary HLA allele carriage status with lifestyle on incident cancer outcomes was significant (P <0.001 after Bonferroni correction); 2) the association between the HLA allele and the incident risk of the corresponding cancer was significant; 3) the association between the lifestyle assessed with HLI and the occurrence of the corresponding cancer was significant.

[b] As HLA allele carrier group increased the corresponding cancer risk, the favorable lifestyle in the HLA allele non-carrier subgroup was considered as the referent in each joint group.

[c] Multivariable-adjusted HRs and 95%CIs were measured by multivariable-adjusted Cox model, with baseline age, height, assessment center, Townsend deprivation index at recruitment, family history of cancer, and population structure (top 5 principal components of the genotypes) adjusted.

[d] The additive interaction analysis was performed with RERI and the 95%CIs were estimated by delta method. P <0.05 was considered statistical significance in this analysis, which were marked in bold.

Supplementary Table S21. Sensitivity analyses for the multivariable-adjusted associations between binary HLA allele carrier status and incident risk of site-specific cancers

Cancer types [a]	HLA alleles	AF (%) [b]	Multivariable-adjusted OR (95%CI) [c]	*P* value [d]
**Men**				
**Prostate cancer**	DQB1*02:01	14.89	0.91 (0.87-0.96)	**8.62E-04**
	DRB1*03:01	14.81	0.90 (0.86-0.95)	**1.54E-04**
**Lung cancer**	DQA1*05:01	23.14	1.22 (1.12-1.33)	**5.03E-06**
	DQB1*02:01	14.89	1.30 (1.18-1.44)	**7.25E-08**
	DRB1*01:01	9.31	0.79 (0.68-0.91)	**9.39E-04**
	DRB1*03:01	14.81	1.30 (1.18-1.43)	**1.22E-07**
	DRB3*01:01	16.71	1.23 (1.12-1.35)	**1.79E-05**
**Kidney cancer**	A*11:01	7.38	1.46 (1.17-1.81)	**6.51E-04**
**Women**				
**Endometrial cancer**	B*07:02	14.78	1.26 (1.11-1.45)	**6.31E-04**
**Kidney cancer**	A*24:02	7.38	1.70 (1.31-2.21)	**7.10E-05**

Abbreviations: AF, allele frequency; HLA, human leukocyte antigen; OR, odds ratio.

[a] Cancer types that were significantly associated with HLA alleles in the logistic regression model adjusted for age at recruitment, assessment center, and population structure (top 5 principal components of the genotypes).

[b] The allele frequency was calculated in men and women separately by simple counting methods where the sum of all allele frequencies at each locus was 1.

[c] The multivariable-adjusted ORs and 95%CIs were computed by considering each HLA allele carrier status as a binary variable (1 indicates the allele carrier and 0 indicates the allele non-carrier status) in the logistic model, treating the allele non-carrier status as the referent.

[d] For each cancer outcome, P<0.001 (0.05/50) was considered statistical significance after Bonferroni correction. All the significant results were included in this table.

Supplementary Table S22. Sensitivity analyses for the multivariable-adjusted associations between lifestyle assessed with six-factor Healthy Lifestyle Index and incident risk of cancer outcomes

Cancer type and lifestyle [a]	Incident cancer cases/ Total	Age-adjusted model [b]		Multivariable-adjusted model [c]	
		HR (95%CI)	*P* value	HR (95%CI)	*P* value
**Men [d]**					
**Prostate cancer**					
Ideal	2496 / 15621	Ref		Ref	
Intermediate	12772 / 133496	0.99 (0.92-1.06)	0.73	0.99 (0.92-1.07)	0.86
Poor	574 / 10627	0.89 (0.79-1.01)	0.06	0.92 (0.82-1.04)	0.18
**Colorectal cancer**					
Favorable	166 / 15621	Ref		Ref	
Intermediate	1950 / 133496	1.38 (1.18-1.62)	**<0.001**	1.38 (1.18-1.61)	**<0.001**
Poor	193 / 10627	1.94 (1.58-2.39)	**<0.001**	1.94 (1.57-2.38)	**<0.001**
**Lung cancer**					
Favorable	30 / 15621	Ref		Ref	
Intermediate	1122 / 133496	4.42 (3.07-6.35)	**<0.001**	4.18 (2.91-6.01)	**<0.001**
Poor	225 / 10627	13.19 (9.01-19.30)	**<0.001**	10.90 (7.44-15.97)	**<0.001**
**Skin cancer**					
Favorable	94 / 15621	Ref		Ref	
Intermediate	908 / 133496	1.14 (0.92-1.41)	0.23	1.16 (0.94-1.44)	0.17
Poor	44 / 10627	0.75 (0.53-1.08)	0.12	0.79 (0.55-1.13)	0.19
**non-Hodgin lymphoma**					
Favorable	71 / 15621	Ref		Ref	
Intermediate	594 / 133496	0.99 (0.77-1.26)	0.91	0.98 (0.77-1.26)	0.90
Poor	50 / 10627	1.17 (0.81-1.68)	0.40	1.15 (0.80-1.65)	0.46
**Kidney cancer**					
Favorable	43 / 15621	Ref		Ref	
Intermediate	527 / 133496	1.44 (1.06-1.97)	0.02	1.44 (1.06-1.97)	0.02
Poor	50 / 10627	1.90 (1.26-2.85)	**0.002**	1.89 (1.26-2.85)	**0.002**
**Bladder cancer**					
Favorable	44 / 15621	Ref		Ref	
Intermediate	507 / 133496	1.36 (0.99-1.85)	0.05	1.35 (0.99-1.84)	0.06
Poor	54 / 10627	2.14 (1.43-3.18)	**<0.001**	2.12 (1.42-3.16)	**<0.001**
**Esophageal cancer**					
Favorable	27 / 15621	Ref		Ref	
Intermediate	452 / 133496	1.97 (1.34-2.91)	**<0.001**	1.94 (1.31-2.85)	**<0.001**
Poor	56 / 10627	3.55 (2.24-5.62)	**<0.001**	3.34 (2.11-5.29)	**<0.001**
**Leukemia**					
Favorable	44 / 15621	Ref		Ref	
Intermediate	442 / 133496	1.18 (0.87-1.61)	0.29	1.19 (0.87-1.62)	0.28
Poor	35 / 10627	1.32 (0.85-2.06)	0.22	1.33 (0.85-2.08)	0.21
**Pancreatic cancer**					
Favorable	27 / 15621	Ref		Ref	
Intermediate	396 / 133496	1.73 (1.17-2.56)	0.006	1.72 (1.17-2.55)	0.006
Poor	33 / 10627	2.08 (1.25-3.46)	0.005	1.99 (1.19-3.33)	0.009
**Gastric cancer**					
Favorable	13 / 15621	Ref		Ref	
Intermediate	287 / 133496	2.60 (1.49-4.53)	**<0.001**	2.52 (1.44-4.39)	**0.001**
Poor	31 / 10627	4.05 (2.12-7.74)	**<0.001**	3.66 (1.91-7.02)	**<0.001**
**Brain cancer**					
Favorable	35 / 15621	Ref		Ref	
Intermediate	269 / 133496	0.91 (0.64-1.29)	0.58	0.90 (0.63-1.28)	0.56
Poor	17 / 10627	0.77 (0.43-1.37)	0.37	0.76 (0.43-1.37)	0.36
**Multiple myeloma**					

Cancer type and lifestyle [a]	Incident cancer cases/ Total	Age-adjusted model [b]		Multivariable-adjusted model [c]	
		HR (95%CI)	*P* value	HR (95%CI)	*P* value
Favorable	27 / 15621	Ref		Ref	
Intermediate	272 / 133496	1.19 (0.80-1.77)	0.39	1.20 (0.81-1.79)	0.36
Poor	14 / 10627	0.87 (0.46-1.66)	0.67	0.90 (0.47-1.72)	0.75
**Liver cancer**					
Favorable	9 / 15621	Ref		Ref	
Intermediate	224 / 133496	2.94 (1.51-5.73)	**0.002**	2.87 (1.47-5.58)	**0.002**
Poor	47 / 10627	8.96 (4.39-18.29)	**<0.001**	8.02 (3.92-16.42)	**<0.001**
**Other cancers [e]**					
Favorable	145 / 15621	Ref		Ref	
Intermediate	1659 / 133496	1.35 (1.14-1.60)	**<0.001**	1.32 (1.11-1.56)	**0.001**
Poor	211 / 10627	2.56 (1.91-2.91)	**<0.001**	2.20 (1.78-2.72)	**<0.001**
**Overall cancer [f]**					
Favorable	1568 / 15621	Ref		Ref	
Intermediate	16247 / 133496	1.22 (1.16-1.29)	**<0.001**	1.22 (1.15-1.28)	**<0.001**
Poor	1475 / 10627	1.58 (1.47-1.70)	**<0.001**	1.56 (1.45-1.68)	**<0.001**
**Women [d]**					
**Breast cancer**					
Favorable	929 / 31641	Ref		Ref	
Intermediate	4990 / 137875	1.24 (1.15-1.33)	**<0.001**	1.25 (1.16-1.34)	**<0.001**
Poor	212 / 5521	1.37 (1.18-1.59)	**<0.001**	1.40 (1.21-1.63)	**<0.001**
**Colorectal cancer**					
Favorable	300 / 31641	Ref		Ref	
Intermediate	1290 / 137875	0.99 (0.87-1.12)	0.82	0.99 (0.87-1.12)	0.85
Poor	50 / 5521	1.13 (0.83-1.52)	0.44	1.13 (0.83-1.52)	0.44
**Lung cancer**					
Favorable	97 / 31641	Ref		Ref	
Intermediate	979 / 137875	2.31 (1.87-2.84)	**<0.001**	2.18 (1.77-2.68)	**<0.001**
Poor	101 / 5521	7.66 (5.79-10.12)	**<0.001**	6.34 (4.78-8.40)	**<0.001**
**Skin cancer**					
Favorable	197 / 31641	Ref		Ref	
Intermediate	772 / 137875	0.90 (0.77-1.05)	0.20	0.92 (0.79-1.08)	0.29
Poor	25 / 5521	0.78 (0.51-1.18)	0.24	0.83 (0.54-1.25)	0.37
**Endometrial cancer**					
Favorable	141 / 31641	Ref		Ref	
Intermediate	805 / 137875	1.31 (1.09-1.57)	**0.003**	1.31 (1.09-1.57)	**0.003**
Poor	39 / 5521	1.81 (1.27-2.58)	**0.001**	1.79 (1.25-2.55)	**0.001**
**Ovarian cancer**					
Favorable	118 / 31641	Ref		Ref	
Intermediate	512 / 137875	1.00 (0.82-1.22)	0.97	1.00 (0.82-1.22)	0.97
Poor	8 / 5521	0.44 (0.21-0.89)	0.02	0.43 (0.21-0.89)	0.02
**non-Hodgin lymphoma**					
Favorable	122 / 31641	Ref		Ref	
Intermediate	472 / 137875	0.89 (0.73-1.08)	0.23	0.89 (0.73-1.08)	0.24
Poor	12 / 5521	0.67 (0.37-1.21)	0.19	0.68 (0.37-1.23)	0.20
**Pancreatic cancer**					
Favorable	57 / 31641	Ref		Ref	
Intermediate	291 / 137875	1.17 (0.88-1.55)	0.28	1.15 (0.87-1.53)	0.34
Poor	20 / 5521	2.50 (1.50-4.16)	**<0.001**	2.40 (1.44-4.01)	**<0.001**
**Kidney cancer**					
Favorable	37 / 31641	Ref		Ref	
Intermediate	280 / 137875	1.74 (1.23-2.44)	**0.002**	1.72 (1.22-2.43)	**0.002**

Cancer type and lifestyle [a]	Incident cancer cases/ Total	Age-adjusted model [b]		Multivariable-adjusted model [c]	
		HR (95%CI)	*P* value	HR (95%CI)	*P* value
Poor	11 / 5521	2.01 (1.02-3.94)	0.04	1.97 (1.00-3.88)	0.05
**Leukemia**					
Favorable	53 / 31641	Ref		Ref	
Intermediate	265 / 137875	1.14 (0.85-1.54)	0.37	1.15 (0.86-1.54)	0.36
Poor	8 / 5521	1.05 (0.50-2.22)	0.89	1.05 (0.50-2.22)	0.89
**Other cancers [e]**					
Favorable	445 / 31641	Ref		Ref	
Intermediate	2116 / 137875	1.09 (0.99-1.21)	0.10	1.08 (0.97-1.19)	0.151
Poor	88 / 5521	1.32 (1.05-1.66)	0.02	1.27 (1.01-1.60)	0.04
**Overall cancer [f]**					
Favorable	2496 / 31641	Ref		Ref	
Intermediate	12772 / 137875	1.17 (1.13-1.23)	**<0.001**	1.17 (1.12-1.22)	**<0.001**
Poor	574 / 5521	1.47 (1.35-1.61)	**<0.001**	1.46 (1.33-1.59)	**<0.001**

Abbreviations: HLI, Healthy Lifestyle Index; CI, confidence interval; HR, hazard ratio.

[a] Participants were classified into having favorable lifestyle (at least 4 ideal lifestyle factors), intermediate lifestyle (between the favorable and the poor), or poor lifestyle (at least 4 poor lifestyle factors) according to the grading criteria in the sensitivity analysis (Supplementary Table S1). All of the studied cancer outcomes were included.

[b] Age-adjusted Cox model was adjusted for the baseline age, treating the favorable lifestyle group as the referent.

[c] Multivariable-adjusted Cox model was adjusted for baseline age, height, Townsend deprivation index at recruitment, and family history of cancer, treating the favorable lifestyle group as the referent.

[d] $P < 0.003125$ (0.05/16) for men and $P < 0.004167$ (0.05/12) for women were applied to determine statistical significance in the analyses respectively after Bonferroni correction. The significant P values were marked in bold.

[e] Among men, other cancers included all malignant cancers except for the 14 listed in this table, of which the top three were mesothelioma (12.21%), oral cancer (11.81%), and tonsil cancer (6.50%). Among women, other cancers included all malignant cancers excluding the 10 in this table, with the top three being multiple myeloma (8.42%), brain cancer (8.23%), and esophageal cancer (7.17%).

[f] Overall cancer was referred to having any one of 342 types of malignant cancers in the UK Biobank.

Supplementary Table S23. Sensitivity analyses for the multivariable-adjusted associations between individual lifestyle factors included in six-factor Healthy Lifestyle Index and incident risk of cancer outcomes among men

Cancer types	Smoking [a]			Alcohol [a]			Physical activity [a]		
	Ideal	Intermediate	Poor	Ideal	Intermediate	Poor	Ideal	Intermediate	Poor
**Prostate cancer**									
Multivariable-adjusted HR (95%CI) [b]	Ref	0.88 (0.83-0.94)	0.83 (0.77-0.91)	Ref	1.17 (0.98-1.39)	1.18 (0.99-1.41)	Ref	1.01 (0.93-1.09)	0.99 (0.93-1.05)
P value		**2.93E-05**	**2.13E-05**		0.08	0.07		0.90	0.72
**Colorectal cancer**									
Multivariable-adjusted HR (95%CI) [b]	Ref	1.25 (1.13-1.38)	1.11 (0.97-1.28)	Ref	1.11 (0.79-1.56)	1.43 (1.01-2.02)	Ref	1.08 (0.94-1.25)	1.17 (1.05-1.29)
P value		**1.21E-05**	0.14		0.56	0.04		0.29	**0.003**
**Lung cancer**									
Multivariable-adjusted HR (95%CI) [b]	Ref	6.30 (5.30-7.47)	14.21 (11.96-16.89)	Ref	0.93 (0.58-1.49)	0.95 (0.59-1.53)	Ref	1.09 (0.90-1.33)	1.15 (1.01-1.31)
P value		**5.80E-98**	**5.26E-199**		0.77	0.84		0.37	0.04
**Skin cancer**									
Multivariable-adjusted HR (95%CI) [b]	Ref	0.87 (0.74-1.02)	0.61 (0.47-0.79)	Ref	1.36 (0.80-2.31)	1.26 (0.73-2.16)	Ref	0.83 (0.66-1.05)	1.01 (0.86-1.17)
P value		0.08	**0.0002**		0.26	0.40		0.12	0.94
**non-Hodgkin lymphoma**									
Multivariable-adjusted HR (95%CI) [b]	Ref	0.90 (0.74-1.08)	1.17 (0.91-1.48)	Ref	0.80 (0.49-1.30)	0.75 (0.45-1.24)	Ref	1.11 (0.86-1.43)	1.00 (0.83-1.21)
P value		0.26	0.21		0.37	0.26		0.43	0.99
**Kidney cancer**									
Multivariable-adjusted HR (95%CI) [b]	Ref	1.15 (0.94-1.40)	1.61 (1.27-2.05)	Ref	1.55 (0.73-3.29)	1.31 (0.61-2.80)	Ref	1.06 (0.81-1.41)	0.99 (0.81-1.21)
P value		0.18	**9.46E-05**		0.25	0.49		0.66	0.89
**Bladder cancer**									
Multivariable-adjusted HR (95%CI) [b]	Ref	1.92 (1.57-2.34)	3.00 (2.38-3.77)	Ref	1.20 (0.59-2.42)	1.24 (0.61-2.52)	Ref	0.92 (0.69-1.23)	0.94 (0.77-1.15)
P value		**1.15E-10**	**8.61E-21**		0.61	0.56		0.60	0.56
**Esophageal cancer**									
Multivariable-adjusted HR (95%CI) [b]	Ref	2.07 (1.68-2.55)	2.73 (2.13-3.51)	Ref	0.96 (0.49-1.87)	0.97 (0.49-1.90)	Ref	0.95 (0.69-1.31)	1.19 (0.97-1.46)
P value		**8.05E-12**	**3.13E-15**		0.91	0.93		0.78	0.10
**Leukemia**									
Multivariable-adjusted HR (95%CI) [b]	Ref	1.27 (1.02-1.58)	1.64 (1.25-2.14)	Ref	0.74 (0.41-1.32)	0.67 (0.37-1.21)	Ref	0.91 (0.67-1.25)	1.04 (0.84-1.29)
P value		0.03	**0.0003**		0.30	0.18		0.57	0.71
**Pancreatic cancer**									
Multivariable-adjusted HR (95%CI) [b]	Ref	1.30 (1.04-1.62)	1.87 (1.43-2.46)	Ref	0.90 (0.44-1.83)	1.13 (0.55-2.31)	Ref	0.94 (0.67-1.31)	1.02 (0.82-1.29)
P value		0.02	**5.54E-06**		0.77	0.74		0.71	0.83
**Gastric cancer**									
Multivariable-adjusted HR (95%CI) [b]	Ref	1.71 (1.31-2.21)	2.12 (1.54-2.92)	Ref	0.82 (0.38-1.75)	0.79 (0.37-1.72)	Ref	1.00 (0.67-1.49)	1.13 (0.86-1.47)
P value		**6.27E-05**	**4.04E-06**		0.60	0.56		0.99	0.37
**Brain cancer**									
Multivariable-adjusted HR (95%CI) [b]	Ref	0.91 (0.68-1.22)	1.00 (0.68-1.45)	Ref	2.69 (0.67-10.87)	2.45 (0.60-9.99)	Ref	1.05 (0.72-1.53)	0.92 (0.69-1.22)
P value		0.54	0.98		0.16	0.21		0.82	0.56

Cancer types	Smoking [a]			Alcohol [a]			Physical activity [a]		
	Ideal	Intermediate	Poor	Ideal	Intermediate	Poor	Ideal	Intermediate	Poor
**Multiple myeloma**									
Multivariable-adjusted HR (95%CI) [b]	Ref	0.73 (0.54-0.99)	0.87 (0.58-1.30)	Ref	0.61 (0.32-1.16)	0.50 (0.26-0.97)	Ref	0.96 (0.65-1.41)	0.96 (0.73-1.27)
P value		0.05	0.49		0.13	0.04		0.83	0.78
**Liver cancer**									
Multivariable-adjusted HR (95%CI) [b]	Ref	1.70 (1.28-2.25)	2.02 (1.42-2.87)	Ref	0.55 (0.25-1.18)	0.88 (0.41-1.91)	Ref	0.87 (0.53-1.43)	1.52 (1.14-2.03)
P value		**0.0003**	**9.35E-05**		0.13	0.76		0.58	0.004
**Other cancers [c]**									
Multivariable-adjusted HR (95%CI) [b]	Ref	1.34 (1.20-1.50)	1.92 (1.70-2.18)	Ref	1.19 (0.82-1.73)	1.30 (0.89-1.90)	Ref	0.96 (0.82-1.13)	1.16 (1.04-1.29)
P value		**2.03E-07**	**1.06E-24**		0.36	0.18		0.65	0.007
**Overall cancer [d]**									
Multivariable-adjusted HR (95%CI) [b]	Ref	1.17 (1.13-1.22)	1.47 (1.40-1.53)	Ref	1.09 (0.98-1.22)	1.13 (1.01-1.27)	Ref	1.00 (0.95-1.05)	1.05 (1.02-1.09)
P value		**6.66E-19**	**3.84E-64**		0.13	0.03		0.99	0.005

Cancer types	Weight [a]			Fruits and vegetables [a]			Red meat and processed meat [a]		
	Ideal	Intermediate	Poor	Ideal	Intermediate	Poor	Ideal	Intermediate	Poor
**Prostate cancer**									
Multivariable-adjusted HR (95%CI) [b]	Ref	1.00 (0.95-1.06)	0.88 (0.82-0.95)	Ref	1.05 (0.99-1.10)	1.02 (0.96-1.09)	Ref	0.99 (0.92-1.07)	1.04 (0.99-1.09)
P value		0.99	**0.0006**		0.06	0.46		0.80	0.15
**Colorectal cancer**									
Multivariable-adjusted HR (95%CI) [b]	Ref	1.18 (1.05-1.32)	1.30 (1.14-1.49)	Ref	1.02 (0.92-1.12)	1.12 (0.99-1.26)	Ref	1.13 (0.98-1.30)	1.17 (1.05-1.29)
P value		0.005	**7.25E-05**		0.72	0.06		0.09	**0.003**
**Lung cancer**									
Multivariable-adjusted HR (95%CI) [b]	Ref	0.88 (0.77-1.01)	0.88 (0.75-1.03)	Ref	1.16 (1.01-1.33)	1.64 (1.42-1.90)	Ref	1.13 (0.94-1.36)	1.18 (1.03-1.36)
P value		0.08	0.11		0.03	**3.26E-11**		0.18	0.01
**Skin cancer**									
Multivariable-adjusted HR (95%CI) [b]	Ref	1.35 (1.14-1.59)	1.38 (1.13-1.68)	Ref	0.96 (0.84-1.10)	0.97 (0.81-1.16)	Ref	0.99 (0.82-1.21)	0.93 (0.81-1.07)
P value		**0.0005**	**0.002**		0.55	0.73		0.95	0.29
**non-Hodgkin lymphoma**									
Multivariable-adjusted HR (95%CI) [b]	Ref	1.11 (0.92-1.35)	1.28 (1.02-1.61)	Ref	0.86 (0.73-1.02)	0.87 (0.71-1.08)	Ref	0.88 (0.69-1.14)	0.99 (0.83-1.17)
P value		0.28	0.03		0.08	0.21		0.33	0.90
**Kidney cancer**									
Multivariable-adjusted HR (95%CI) [b]	Ref	1.38 (1.09-1.75)	2.24 (1.74-2.89)	Ref	1.18 (0.98-1.42)	1.24 (0.99-1.56)	Ref	0.78 (0.59-1.04)	1.04 (0.86-1.25)
P value		0.007	**4.99E-10**		0.09	0.06		0.09	0.69
**Bladder cancer**									
Multivariable-adjusted HR (95%CI) [b]	Ref	1.07 (0.86-1.33)	1.31 (1.02-1.68)	Ref	1.13 (0.94-1.36)	1.05 (0.83-1.33)	Ref	1.00 (0.76-1.32)	1.06 (0.87-1.28)
P value		0.56	0.03		0.21	0.69		0.99	0.57
**Esophageal cancer**									

Cancer types	Weight [a]			Fruits and vegetables [a]			Red meat and processed meat [a]		
	Ideal	Intermediate	Poor	Ideal	Intermediate	Poor	Ideal	Intermediate	Poor
Multivariable-adjusted HR (95%CI) [b]	Ref	1.38 (1.07-1.79)	2.01 (1.52-2.67)	Ref	1.12 (0.92-1.37)	1.23 (0.97-1.56)	Ref	0.87 (0.64-1.19)	1.15 (0.93-1.41)
P value		0.01	**9.06E-07**		0.27	0.09		0.39	0.19
**Leukemia**									
Multivariable-adjusted HR (95%CI) [b]	Ref	1.10 (0.88-1.38)	1.07 (0.82-1.41)	Ref	0.92 (0.75-1.12)	1.13 (0.89-1.44)	Ref	0.92 (0.69-1.23)	1.00 (0.82-1.22)
P value		0.42	0.62		0.40	0.32		0.57	0.98
**Pancreatic cancer**									
Multivariable-adjusted HR (95%CI) [b]	Ref	1.18 (0.91-1.53)	1.55 (1.16-2.07)	Ref	0.93 (0.75-1.14)	0.98 (0.75-1.27)	Ref	1.49 (1.11-2.02)	1.24 (0.99-1.57)
P value		0.21	**0.003**		0.47	0.86		0.009	0.07
**Gastric cancer**									
Multivariable-adjusted HR (95%CI) [b]	Ref	1.70 (1.22-2.38)	1.86 (1.28-2.70)	Ref	0.69 (0.54-0.90)	1.08 (0.81-1.43)	Ref	1.14 (0.80-1.62)	1.06 (0.82-1.37)
P value		**0.002**	**0.001**		0.006	0.61		0.48	0.67
**Brain cancer**									
Multivariable-adjusted HR (95%CI) [b]	Ref	0.95 (0.73-1.25)	0.85 (0.60-1.20)	Ref	1.08 (0.84-1.39)	0.91 (0.66-1.27)	Ref	0.82 (0.56-1.20)	1.02 (0.79-1.31)
P value		0.73	0.36		0.53	0.59		0.29	0.87
**Multiple myeloma**									
Multivariable-adjusted HR (95%CI) [b]	Ref	1.31 (0.97-1.77)	1.33 (0.93-1.91)	Ref	1.09 (0.85-1.40)	1.08 (0.78-1.49)	Ref	1.48 (1.05-2.09)	1.10 (0.84-1.44)
P value [c]		0.07	0.12		0.50	0.65		0.03	0.50
**Liver cancer**									
Multivariable-adjusted HR (95%CI) [b]	Ref	1.18 (0.81-1.73)	2.72 (1.85-3.99)	Ref	0.97 (0.73-1.28)	1.13 (0.82-1.56)	Ref	1.09 (0.72-1.65)	1.26 (0.93-1.69)
P value		0.39	**3.45E-07**		0.82	0.46		0.70	0.13
**Other cancers [c]**									
Multivariable-adjusted HR (95%CI) [b]	Ref	0.92 (0.82-1.03)	1.01 (0.88-1.15)	Ref	1.00 (0.90-1.11)	1.22 (1.08-1.38)	Ref	0.83 (0.71-0.97)	1.05 (0.95-1.17)
P value		0.15	0.92		0.97	**0.001**		0.02	0.35
**Overall cancer [d]**									
Multivariable-adjusted HR (95%CI) [b]	Ref	1.05 (1.02-1.09)	1.10 (1.05-1.14)	Ref	1.03 (0.99-1.06)	1.11 (1.06-1.15)	Ref	1.00 (0.95-1.05)	1.07 (1.03-1.10)
P value		0.006	**5.98E-05**		0.12	**1.09E-06**		0.95	**0.0002**

Abbreviations: CI, confidence interval; HR, hazard ratio.

[a] Each individual lifestyle factor (smoking, alcohol, physical activity, weight status, fruits and vegetables, and red meat and processed meat) was classified into ideal, intermediate, and poor according to the criteria shown in Supplementary Table S1.

[b] Multivariable-adjusted Cox model was adjusted for baseline age, height, Townsend deprivation index at recruitment, and family history of cancer, treating the ideal lifestyle factor as the referent. P <0.003125 (=0.05/16) was considered statistical significance after Bonferroni correction and marked in bold.

[c] Other cancers included all malignant cancers except for the 14 listed in the table, of which the top three were mesothelioma (12.21%), oral cancer (11.81%), and tonsil cancer (6.50%).

[d] Overall cancer was referred to having any one of 342 types of malignant cancer in the UK Biobank.

Supplementary Table S24. Sensitivity analyses for the multivariable-adjusted associations between individual lifestyle factors included in six-factor Healthy Lifestyle Index and incident risk of cancer outcomes among women

Cancer types	Smoking [a]			Alcohol [a]			Physical activity [a]		
	Ideal	Intermediate	Poor	Ideal	Ideal	Intermediate	Poor	Intermediate	Ideal
**Breast cancer**									
Multivariable-adjusted HR (95%CI) [b]	Ref	1.04 (0.97-1.11)	1.12 (1.02-1.23)	Ref	1.05 (0.92-1.20)	1.19 (1.04-1.36)	Ref	1.04 (0.95-1.14)	1.08 (1.01-1.15)
P value		0.30	0.02		0.47	0.01		0.35	0.03
**Colorectal cancer**									
Multivariable-adjusted HR (95%CI) [b]	Ref	1.31 (1.15-1.49)	1.15 (0.95-1.39)	Ref	0.86 (0.69-1.07)	0.86 (0.68-1.09)	Ref	1.17 (0.99-1.38)	1.05 (0.92-1.20)
P value		**4.98E-05**	0.14		0.17	0.20		0.07	0.46
**Lung cancer**									
Multivariable-adjusted HR (95%CI) [b]	Ref	5.93 (5.04-6.96)	14.29 (12.14-16.83)	Ref	1.05 (0.78-1.41)	1.02 (0.75-1.38)	Ref	0.84 (0.67-1.06)	1.09 (0.94-1.27)
P value		**1.10E-103**	**1.71E-224**		0.75	0.92		0.14	0.26
**Skin cancer**									
Multivariable-adjusted HR (95%CI) [b]	Ref	0.77 (0.63-0.94)	0.71 (0.53-0.94)	Ref	1.23 (0.87-1.72)	1.21 (0.85-1.72)	Ref	0.85 (0.67-1.07)	0.95 (0.80-1.12)
P value		0.009	0.02		0.24	0.30		0.17	0.52
**Endometrial cancer**									
Multivariable-adjusted HR (95%CI) [b]	Ref	0.78 (0.65-0.94)	0.58 (0.43-0.78)	Ref	0.77 (0.59-1.00)	0.77 (0.58-1.03)	Ref	1.13 (0.91-1.41)	1.14 (0.96-1.34)
P value		0.009	**0.0004**		0.05	0.08		0.26	0.13
**Ovarian cancer**									
Multivariable-adjusted HR (95%CI) [b]	Ref	1.17 (0.94-1.45)	1.07 (0.79-1.45)	Ref	0.85 (0.59-1.22)	0.91 (0.62-1.33)	Ref	1.17 (0.90-1.52)	0.87 (0.70-1.08)
P value		0.16	0.66		0.38	0.61		0.24	0.20
**non-Hodgkin lymphoma**									
Multivariable-adjusted HR (95%CI) [b]	Ref	0.92 (0.73-1.16)	0.89 (0.63-1.24)	Ref	1.09 (0.74-1.59)	0.82 (0.55-1.24)	Ref	0.98 (0.73-1.32)	1.12 (0.90-1.38)
P value		0.48	0.48		0.67	0.35		0.89	0.30
**Pancreatic cancer**									
Multivariable-adjusted HR (95%CI) [b]	Ref	1.16 (0.86-1.55)	2.53 (1.85-3.46)	Ref	0.85 (0.54-1.33)	0.89 (0.55-1.43)	Ref	1.36 (0.97-1.92)	1.29 (0.99-1.68)
P value		0.33	**5.49E-09**		0.48	0.62		0.08	0.06
**Kidney cancer**									
Multivariable-adjusted HR (95%CI) [b]	Ref	1.50 (1.13-1.99)	1.83 (1.27-2.63)	Ref	1.40 (0.75-2.59)	1.04 (0.54-1.99)	Ref	1.13 (0.75-1.69)	1.40 (1.05-1.85)
P value		0.005	**0.0011**		0.29	0.91		0.57	0.02
**Leukemia**									
Multivariable-adjusted HR (95%CI) [b]	Ref	1.25 (0.93-1.67)	1.01 (0.65-1.57)	Ref	1.18 (0.68-2.06)	1.09 (0.61-1.94)	Ref	1.42 (0.99-2.02)	1.02 (0.75-1.39)
P value		0.14	0.97		0.55	0.78		0.06	0.88
**Other cancers [c]**									
Multivariable-adjusted HR (95%CI) [b]	Ref	1.31 (1.18-1.46)	1.62 (1.43-1.85)	Ref	0.84 (0.70-1.00)	0.79 (0.66-0.95)	Ref	0.95 (0.83-1.10)	1.02 (0.92-1.13)
P value [c]		**2.66E-07**	**2.24E-13**		0.04	0.01		0.48	0.76
**Overall cancer [d]**									
Multivariable-adjusted HR (95%CI) [b]	Ref	1.22 (1.17-1.28)	1.52 (1.45-1.61)	Ref	0.96 (0.89-1.04)	0.99 (0.91-1.07)	Ref	1.04 (0.98-1.10)	1.06 (1.02-1.10)

Cancer types	Smoking [a]			Alcohol [a]			Physical activity [a]		
	Ideal	Intermediate	Poor	Ideal	Ideal	Intermediate	Poor	Intermediate	Ideal
P value [c]		**1.66E-20**	**1.00E-53**		0.34	0.72		0.21	0.006

Cancer types	Weight [a]			Fruits and vegetables [a]			Red meat and processed meat [a]		
	Ideal	Intermediate	Poor	Ideal	Ideal	Intermediate	Poor	Intermediate	Ideal
**Breast cancer**									
Multivariable-adjusted HR (95%CI) [b]	Ref	1.16 (1.09-1.23)	1.26 (1.17-1.35)	Ref	1.11 (1.05-1.17)	1.09 (1.00-1.18)	Ref	0.97 (0.88-1.08)	1.07 (1.02-1.13)
P value		**2.94E-06**	**1.15E-09**		**0.0003**	0.05		0.61	0.01
**Colorectal cancer**									
Multivariable-adjusted HR (95%CI) [b]	Ref	1.01 (0.90-1.13)	1.00 (0.87-1.15)	Ref	1.03 (0.93-1.15)	1.02 (0.86-1.20)	Ref	0.96 (0.79-1.16)	1.00 (0.90-1.11)
P value		0.89	0.99		0.58	0.85		0.65	0.98
**Lung cancer**									
Multivariable-adjusted HR (95%CI) [b]	Ref	1.14 (0.98-1.32)	1.09 (0.92-1.30)	Ref	1.07 (0.94-1.22)	1.18 (0.99-1.41)	Ref	1.26 (1.02-1.56)	1.18 (1.04-1.34)
P value		0.08	0.31		0.31	0.05		0.03	0.01
**Skin cancer**									
Multivariable-adjusted HR (95%CI) [b]	Ref	1.01 (0.87-1.16)	0.97 (0.81-1.17)	Ref	0.85 (0.74-0.97)	0.86 (0.69-1.07)	Ref	0.96 (0.75-1.24)	1.05 (0.92-1.20)
P value		0.93	0.77		0.02	0.18		0.76	0.45
**Endometrial cancer**									
Multivariable-adjusted HR (95%CI) [b]	Ref	1.56 (1.29-1.88)	3.75 (3.09-4.54)	Ref	1.09 (0.95-1.25)	1.02 (0.82-1.27)	Ref	0.87 (0.67-1.12)	1.03 (0.90-1.18)
P value		**4.79E-06**	**1.07E-41**		0.20	0.87		0.28	0.63
**Ovarian cancer**									
Multivariable-adjusted HR (95%CI) [b]	Ref	1.00 (0.82-1.20)	1.22 (0.98-1.52)	Ref	0.93 (0.79-1.11)	0.96 (0.73-1.26)	Ref	0.98 (0.72-1.32)	0.93 (0.79-1.10)
P value		0.96	0.08		0.44	0.76		0.88	0.41
**non-Hodgkin lymphoma**									
Multivariable-adjusted HR (95%CI) [b]	Ref	0.97 (0.80-1.17)	1.13 (0.90-1.42)	Ref	1.02 (0.85-1.21)	1.19 (0.91-1.56)	Ref	0.82 (0.58-1.15)	0.97 (0.82-1.15)
P value		0.74	0.29		0.85	0.20		0.24	0.75
**Pancreatic cancer**									
Multivariable-adjusted HR (95%CI) [b]	Ref	0.96 (0.74-1.23)	1.37 (1.03-1.82)	Ref	0.89 (0.71-1.12)	0.83 (0.58-1.20)	Ref	1.37 (0.94-1.98)	1.09 (0.88-1.37)
P value		0.73	0.03		0.32	0.32		0.10	0.43
**Kidney cancer**									
Multivariable-adjusted HR (95%CI) [b]	Ref	1.42 (1.06-1.90)	1.83 (1.32-2.53)	Ref	1.23 (0.97-1.56)	1.19 (0.83-1.70)	Ref	0.97 (0.64-1.48)	0.97 (0.77-1.22)
P value		0.02	**0.0003**		0.08	0.34		0.89	0.82
**Leukemia**									
Multivariable-adjusted HR (95%CI) [b]	Ref	1.01 (0.78-1.31)	1.00 (0.73-1.38)	Ref	0.94 (0.73-1.19)	1.12 (0.77-1.63)	Ref	0.97 (0.63-1.50)	0.99 (0.78-1.24)
P value		0.93	0.98		0.59	0.54		0.89	0.91
**Other cancers [d]**									
Multivariable-adjusted HR (95%CI) [b]	Ref	0.99 (0.90-1.08)	1.04 (0.93-1.16)	Ref	1.00 (0.92-1.09)	1.15 (1.01-1.30)	Ref	1.00 (0.86-1.16)	0.98 (0.90-1.06)
P value		0.76	0.47		0.94	0.03		0.95	0.57

**Cancer types**	**Weight** [a]			**Fruits and vegetables** [a]			**Red meat and processed meat** [a]		
	**Ideal**	**Intermediate**	**Poor**	**Ideal**	**Ideal**	**Intermediate**	**Poor**	**Intermediate**	**Ideal**
**Overall cancer** [e]									
Multivariable-adjusted HR (95%CI) [b]	Ref	1.09 (1.05-1.13)	1.24 (1.19-1.30)	Ref	1.05 (1.01-1.08)	1.08 (1.03-1.14)	Ref	0.99 (0.93-1.06)	1.04 (1.01-1.07)
P value [c]		**9.86E-06**	**9.17E-21**		0.01	**0.003**		0.81	0.02

Abbreviations: CI, confidence interval; HR, hazard ratio.

[a] Each individual lifestyle factor (smoking, alcohol, physical activity, weight status, fruits and vegetables, and red meat and processed meat) was classified into ideal, intermediate, and poor according to the criteria shown in Supplementary Table S1.

[b] Multivariable-adjusted Cox model was adjusted for baseline age, height, Townsend deprivation index at recruitment, and family history of cancer, treating the ideal lifestyle factor as the referent. P <0.004167 (=0.05/12) was considered statistical significance after Bonferroni correction and marked in bold.

[c] Other cancers included all malignant cancers excluding the 10 listed in the table, with the top three being multiple myeloma (8.42%), brain cancer (8.23%), and esophageal cancer (7.17%).

[d] Overall cancer was referred to having any one of 342 types of malignant cancer in the UK Biobank.

Supplementary Table S25. Significant interactions between HLA-DQB1*05:01 and lifestyle as well as individual lifestyle factors on the incident risk of lung cancer among men

HLA allele carriage status [a]	Lifestyle groups [b]	Cases No.	Incidence rates per 1000 person-years	Multivariable-adjusted HR (95%CI) [c]	Additive interaction [d]	
					RERI (95%CI)	*P* value
**Lifestyle**						
DQB1*05:01 carrier	Favorable	8	0.25	Ref		
	Intermediate	120	0.44	1.86 (0.91-3.80)		
	Poor	142	1.25	4.58 (2.25-9.35)		
DQB1*05:01 non-carrier	Favorable	15	0.14	0.57 (0.24-1.34)		
	Intermediate	538	0.57	2.43 (1.21-4.88)	1.01 (0.52-1.49)	**<0.001**
	Poor	689	1.70	6.20 (3.09-12.45)	2.05 (0.87-3.23)	**<0.001**
**Smoking**						
DQB1*05:01 carrier	Favorable	50	0.21	Ref		
	Unfavorable	203	1.49	6.16 (4.50-8.42)		
DQB1*05:01 non-carrier	Favorable	160	0.20	0.95 (0.69-1.31)		
	Unfavorable	1006	2.09	8.58 (6.43-11.44)	2.51 (1.40-3.62)	**<0.001**
**Alcohol**						
DQB1*05:01 carrier	Favorable	4	0.55	Ref		
	Unfavorable	262	0.64	0.90 (0.33-2.41)		
DQB1*05:01 non-carrier	Favorable	18	0.77	1.41 (0.48-4.16)		
	Unfavorable	1198	0.84	1.16 (0.44-3.11)	0.07 (-1.07-1.21)	0.45
**Physical activity**						
DQB1*05:01 carrier	Favorable	118	0.53	Ref		
	Unfavorable	103	0.78	1.30 (0.99-1.69)		
DQB1*05:01 non-carrier	Favorable	546	0.72	1.32 (1.09-1.62)		
	Unfavorable	441	0.93	1.53 (1.25-1.88)	-0.07 (-0.46-0.32)	0.63
**Weight**						
DQB1*05:01 carrier	Favorable	211	0.64	Ref		
	Unfavorable	55	0.63	0.71 (0.53-0.95)		
DQB1*05:01 non-carrier	Favorable	921	0.80	1.25 (1.08-1.45)		
	Unfavorable	314	1.02	1.11 (0.93-1.32)	0.25 (-0.06-0.55)	0.06
**Diet**						
DQB1*05:01 carrier	Favorable	37	0.42	Ref		
	Unfavorable	233	0.70	1.44 (1.02-2.04)		
DQB1*05:01 non-carrier	Favorable	165	0.54	1.28 (0.89-1.83)		
	Unfavorable	1077	0.93	1.89 (1.36-2.62)	0.27 (-0.14-0.68)	0.10

Abbreviations: CI, confidence interval; HR, hazard ratio; RERI, relative excess risk due to interaction.

[a] As DQB1*05:01 increased lung cancer risk, the favorable lifestyle group in DQB1*05:01 carrier group was considered as the referent in the joint group.

[b] Participants were categorized as favorable lifestyle, intermediate lifestyle, or poor lifestyle based on Healthy Lifestyle Index score. Each individual lifestyle factor (smoking, alcohol, physical activity, weight, and diet) was classified into favorable and unfavorable according to the criteria shown in Supplementary Table S1.

[c] Multivariable-adjusted HRs and 95%CIs were measured by multivariable-adjusted Cox model, with baseline age, height, assessment center, Townsend deprivation index at recruitment, family history of cancer and population structure (top 5 principal components of the genotypes) adjusted.

[d] The additive interaction was performed with RERI, with 95%CIs estimated by the delta method. P<0.05 for overall lifestyle and individual lifestyle factors were considered statistical significance and marked in bold.

Supplementary Table S26. Significant associations between HLA allele dosage and incident site-specific cancer risk estimated using Cox proportional hazards models

**Cancer types** [a]	**HLA allele**	**AF (%)** [b]	**Multivariable-adjusted HR (95%CI)**[c]	***P* value** [d]
**Men**				
**Prostate cancer**	C*03:04	8.03	1.10 (1.04-1.17)	**4.40E-04**
	C*04:01	8.24	0.90 (0.85-0.96)	**7.51E-05**
	DQB1*02:01	14.89	0.92 (0.88-0.97)	**6.37E-04**
	DRB1*03:01	14.81	0.91 (0.87-0.96)	**1.31E-04**
**Lung cancer**	B*08:01	14.47	1.19 (1.08-1.32)	**6.35E-04**
	DQA1*05:01	23.14	1.22 (1.12-1.33)	**4.78E-06**
	DQB1*02:01	14.89	1.30 (1.18-1.44)	**5.56E-08**
	DRB1*01:01	9.31	0.79 (0.68-0.91)	**9.39E-04**
	DRB1*03:01	14.81	1.30 (1.18-1.43)	**9.45E-08**
	DRB3*01:01	16.71	1.23 (1.12-1.35)	**2.01E-05**
**non-Hodgkin lymphoma**	DRB1*01:01	9.31	1.36 (1.16-1.59)	**1.69E-04**
**Women**				
**Endometrial cancer**	B*07:02	14.78	1.23 (1.09-1.38)	**5.27E-04**
**Kidney cancer**	A*24:02	7.38	1.60 (1.25-2.03)	**1.59E-04**
	DPB1*04:01	43.8	1.30 (1.11-1.52)	**8.95E-04**

Abbreviations: AF, allele frequency; CI, confidence interval; HLA, human leukocyte antigen; HR, hazard ratio.

[a] Cancer types that were significantly associated with HLA allele dosage in the Cox model adjusted for age at recruitment, assessment center, and population structure (top 5 principal components of the genotypes).

[b] The allele frequency was calculated in men and women separately by simple counting methods where the sum of all allele frequencies at each locus was 1.

[c] The multivariable-adjusted HRs and 95%CIs were computed by using the dosage of each HLA allele as a continuous variable in the Cox regression model.

[d] For each cancer outcome, $P < 0.001$ (=0.05/50) was considered statistical significance in this analysis after Bonferroni correction. All significant associations were included in this table.

Supplementary Table S27. Stratified bootstrap validation results of significant additive interactions identified in the primary analyses

Cancer types	Joint group	Additive interaction RERI (95%CI) [a]
**Men**		
**Lung cancer**	**B*08:01 carrier**	
	Intermediate lifestyle	0.72 (-0.19-1.49)
	Poor lifestyle	**2.82 (1.35-5.01)**
	**DQA1*05:01 carrier**	
	Intermediate lifestyle	0.70 (-0.10-1.23)
	Poor lifestyle	**3.00 (1.84-4.90)**
	**DQB1*02:01 carrier**	
	Intermediate lifestyle	0.88 (-0.06-1.66)
	Poor lifestyle	**3.34 (1.83-5.62)**
	**DRB1*03:01 carrier**	
	Intermediate lifestyle	0.84 (-0.08-1.59)
	Poor lifestyle	**3.36 (1.85-5.65)**
**Women**		
**Kidney cancer**	**A*24:02 carrier**	
	Intermediate lifestyle	**1.20 (0.23-2.15)**
	Poor lifestyle	**3.38 (1.57-6.07)**

Abbreviations: CI, confidence interval; RERI, relative excess risk due to interaction.

[a] Non-parametric bootstrap with 1,000 resamples was used to obtain percentile-based 95% CIs for the RERI. No *P* values were calculated in the bootstrap analyses. RERI estimates with 95% CIs excluding 0 are shown in bold.

Supplementary Table S28. Assessment of model influence and sparse-cell robustness for significant HLA–lifestyle interactions identified in the primary analyses

Cancer type	Joint-exposure group	No. cases removed[a]	Maximum |DFBETA| in sparsest cell [b]	Influence threshold [b]	Original RERI (95% CI)[c]	RERI (95% CI) after sparse-cell case exclusion [d]
Men						
**Lung cancer**	**B*08:01**	3	0.000713	0.005004		
	Intermediate vs favorable				0.72 (-0.19-1.49)	**1.20 (0.65-1.75)**
	Poor vs favorable				**2.82 (1.35-5.01)**	**3.12 (1.49-4.76)**
**Lung cancer**	**DQA1*05:01**	5	0.000804	0.005004		
	Intermediate vs favorable				0.70 (-0.10-1.23)	**1.19 (0.74-1.65)**
	Poor vs favorable				**3.00 (1.84-4.90)**	**3.42 (1.83-5.00)**
**Lung cancer**	**DQB1*02:01**	4	0.000598	0.005004		
	Intermediate vs favorable				0.88 (-0.06-1.66)	**1.53 (0.90-2.16)**
	Poor vs favorable				**3.34 (1.83-5.62)**	**3.93 (1.96-5.90)**
**Lung cancer**	**DRB1*03:01**	4	0.000597	0.005004		
	Intermediate vs favorable				0.84 (-0.08-1.59)	**1.49 (0.87-2.11)**
	Poor vs favorable				**3.36 (1.85-5.65)**	**3.95 (1.97-5.93)**
Women						
**Kidney cancer**	**A*24:02**	2	0.000236	0.00478		
	Intermediate vs favorable				**1.20 (0.23-2.15)**	**1.70 (0.97-2.43)**
	Poor vs favorable				**3.38 (1.57-6.07)**	**3.74 (1.72-5.77)**

Abbreviations: CI, confidence interval; HLA, human leukocyte antigen; RERI, relative excess risk due to interaction.

[a] The sparsest joint-exposure cell was defined as the HLA-carrier/favorable-lifestyle stratum for each significant HLA–lifestyle interaction shown in Figure 3

[b] Dfbetas are standardized regression-deletion diagnostics estimated from the corresponding multivariable-adjusted Cox model, the maximum absolute DFBETA among cases in the sparsest joint-exposure cell is reported. The influence threshold was calculated as $2/\sqrt{n}$, where n is the total sample size.

[c] The original RERI (95%CI) was reported in Figure 3

[d] The corresponding RERI (95%CI) was estimated after removing all cases from the sparsest joint-exposure cell for each reported significant interaction. RERI estimates with 95% CIs excluding 0 are shown in bold.

Supplementary Table S29. Sensitivity analysis of HLA–lifestyle interactions on lung cancer risk in men after additional adjustment for smoking-related variables

Joint group[a]	Multivariable-adjusted HR (95%CI) [b]	Additive interaction	
		RERI (95%CI) [c]	*P* value[c]
**B*08:01 non-carrier**			
Favorable lifestyle	Ref		
Intermediate lifestyle	1.09 (0.67-1.77)		
Poor lifestyle	1.29 (0.78-2.13)		
**B*08:01 carrier**			
Favorable lifestyle	0.46 (0.14-1.56)		
Intermediate lifestyle	1.14 (0.69-1.89)	0.59 (0.00-1.19)	**0.025**
Poor lifestyle	1.75 (1.05-2.91)	1.00 (0.41-1.58)	**<0.001**
**DQA1*05:01 non-carrier**			
Favorable lifestyle	Ref		
Intermediate lifestyle	0.98 (0.58-1.64)		
Poor lifestyle	1.11 (0.65-1.88)		
**DQA1*05:01 carrier**			
Favorable lifestyle	0.45 (0.17-1.24)		
Intermediate lifestyle	1.04 (0.61-1.75)	0.61 (0.13-1.08)	**0.006**
Poor lifestyle	1.53 (0.90-2.60)	0.97 (0.51-1.42)	**<0.001**
**DQB1*02:01 non-carrier**			
Favorable lifestyle	Ref		
Intermediate lifestyle	1.13 (0.68-1.86)		
Poor lifestyle	1.35 (0.81-2.25)		
**DQB1*02:01 carrier**			
Favorable lifestyle	0.64 (0.21-1.89)		
Intermediate lifestyle	1.25 (0.75-2.10)	0.49 (-0.22-1.19)	0.088
Poor lifestyle	1.86 (1.10-3.12)	0.87 (0.18-1.56)	**0.006**
**DRB1*03:01 non-carrier**			
Favorable lifestyle	Ref		
Intermediate lifestyle	1.13 (0.69-1.87)		
Poor lifestyle	1.35 (0.81-2.25)		
**DRB1*03:01 carrier**			
Favorable lifestyle	0.64 (0.21-1.90)		
Intermediate lifestyle	1.24 (0.74-2.08)	0.47 (-0.24-1.18)	0.097
Poor lifestyle	1.86 (1.11-3.13)	0.87 (0.19-1.56)	**0.006**

Abbreviations: CI, confidence interval; HLA, human leukocyte antigen; HR, hazard ration; RERI, relative excess risk due to interaction.

[a]The listed HLA alleles are same as those in Figure 3. For each allele, the carriers significantly increased lung cancer risk compared to non-carriers.

[b] Multivariable-adjusted HRs and 95% CIs were estimated using Cox proportional hazards models, with the non-carrier/favorable-lifestyle group as the reference category. In addition to the covariates included in the primary analyses (age, assessment center, height, Townsend deprivation index, family history of cancer, and the first five principal components of genetic ancestry), these models were further adjusted for detailed smoking-related variables, including pack-years, age at smoking cessation, and smoking cessation duration.

[c] Additive interactions were quantified by the RERI with 95% CIs estimated using the delta method. The statistically significant *P* values are shown in bold (P<0.05).

Supplementary Table S30. Sensitivity analysis of the associations between lifestyle and female-specific cancer risk with additional adjustment for reproductive and hormone-related variables

Cancer types	Multivariable-adjusted HR (95% CI)[a]	$HR_{continuous}$[b]	*P*-trend[c]
**Breast cancer**		1.09 (1.06-1.12)	**<0.001**
Intermediate vs. Favorable	1.21 (1.10-1.33)		
Poor vs. Favorable	1.36 (1.23-1.51)		
**Endometrial cancer**		1.25 (1.17-1.33)	**<0.001**
Intermediate vs. Favorable	1.29 (1.03-1.62)		
Poor vs. Favorable	1.99 (1.56-2.54)		
**Ovarian cancer**		1.08 (1.00-1.17)	0.01
Intermediate vs. Favorable	1.31 (0.99-1.74)		
Poor vs. Favorable	1.48 (1.08-2.03)		

Abbreviations: CI, confidence interval; HLI, healthy lifestyle index; HR, hazard ratio; HRT, hormone replacement therapy

[a] HRs and 95%CIs were estimated from multivariable-adjusted Cox proportional hazards models with the favorable lifestyle category as the reference group. In addition to the standard covariates (baseline age, height, Townsend deprivation index, family history of cancer), models were additionally adjusted for reproductive and hormonal factors: age at menarche (continuous), menopausal status (yes, no, not sure), number of live births (continuous), age at first live birth (continuous), ever use of oral contraceptive pills (yes, no) and ever use of HRT (yes, no).

[b] $HR_{continuous}$ represents the HR per one-unit decrease in the HLI

[c] P-trend was assessed using the median HLI value within each lifestyle category in the corresponding Cox model. Statistically significant P values are shown in bold. A Bonferroni-corrected significance threshold of $P < 0.004167$ (0.05/12) was applied to all analyses.

Supplementary Table S31. Complete-case sensitivity analysis of the joint effects of cancer-related HLA alleles and lifestyle categories on cancer risk

Cancer types[a]	Joint group[a]	Case / Total	Incidence rates per 1000 person-years	Multivariable-adjusted HR (95%CI)[b]	Absolute risk difference (%)-5 years (95% CI)[c]	Additive interaction[d]	
						RERI (95%CI)	*P* value
Men							
**Lung cancer**	**B*08:01 non-carrier**						
	Favorable lifestyle	18/8808	0.17	Ref	Ref		
	Intermediate lifestyle	423/74981	0.48	2.86 (1.79-4.59)*	0.14 (0.09-0.18)		
	Poor lifestyle	514/32692	1.36	7.07 (4.42-11.32)*	0.54 (0.48-0.62)		
	**B*08:01 carrier**						
	Favorable lifestyle	3/3100	0.08	0.45 (0.13-1.53)	-0.04 (-0.09-0.03)		
	Intermediate lifestyle	167/27523	0.51	3.06 (1.88-4.98)*	0.15 (0.12-0.18)	0.75 (0.00-1.50)	**0.03**
	Poor lifestyle	245/12068	1.76	9.19 (5.69-14.84)*	0.73 (0.61-0.82)	2.67 (1.07-4.27)	**0.001**
**Lung cancer**	**DQA1*05:01 non-carrier**						
	Favorable lifestyle	16/7110	0.19	Ref	Ref		
	Intermediate lifestyle	339/60528	0.47	2.60 (1.57-4.29)*	0.13 (0.10-0.17)		
	Poor lifestyle	387/26478	1.26	6.03 (3.66-9.95)*	0.49 (0.39-0.55)		
	**DQA1*05:01 carrier**						
	Favorable lifestyle	5/4798	0.09	0.46 (0.17-1.26)	-0.05 (-0.09--0.01)		
	Intermediate lifestyle	251/41976	0.50	2.79 (1.68-4.63)*	0.14 (0.10-0.18)	0.73 (0.12-1.35)	**0.01**
	Poor lifestyle	372/18282	1.76	8.44 (5.12-13.93)*	0.72 (0.57-0.84)	2.95 (1.47-4.42)	**<0.001**
**Lung cancer**	**DQB1*02:01 non-carrier**						
	Favorable lifestyle	17/8711	0.16	Ref	Ref		
	Intermediate lifestyle	407/74279	0.46	2.94 (1.81-4.77)*	0.14 (0.11-0.17)		
	Poor lifestyle	494/32408	1.31	7.27 (4.48-11.80)*	0.53 (0.49-0.61)		
	**DQB1*02:01 carrier**						
	Favorable lifestyle	4/3197	0.10	0.63 (0.21-1.88)	-0.03 (-0.09-0.01)		
	Intermediate lifestyle	183/28225	0.54	3.46 (2.11-5.69)*	0.17 (0.12-0.21)	0.89 (0.04-1.74)	**0.02**
	Poor lifestyle	265/12352	1.86	10.19 (6.24-16.65)*	0.78 (0.68-0.86)	3.29 (1.42-5.16)	**<0.001**
**Lung cancer**	**DRB1*03:01 non-carrier**						
	Favorable lifestyle	17/8732	0.16	Ref	Ref		
	Intermediate lifestyle	409/74411	0.46	2.95 (1.82-4.79)*	0.14 (0.09-0.17)		
	Poor lifestyle	494/32440	1.31	7.27 (4.48-11.80)*	0.53 (0.48-0.57)		

	**DRB1*03:01 carrier**						
	Favorable lifestyle	4/3176	0.10	0.64 (0.21-1.89)	-0.03 (-0.09-0.02)		
	Intermediate lifestyle	181/28093	0.54	3.44 (2.09-5.65)*	0.17 (0.13-0.22)	0.85 (0.00-1.71)	**0.03**
	Poor lifestyle	265/12320	1.87	10.22 (6.25-16.70)*	0.78 (0.70-0.89)	3.31 (1.43-5.19)	**<0.001**
Women							
**Kidney cancer**	**A*24:02 non-carrier**						
	Favorable lifestyle	23/17850	0.11	Ref	Ref		
	Intermediate lifestyle	155/97407	0.13	1.33 (0.86-2.06)	0.01 (-0.01-0.03)		
	Poor lifestyle	77/34453	0.19	1.89 (1.19-3.03)*	0.04 (0.02-0.06)		
	**A*24:02 carrier**						
	Favorable lifestyle	2/3083	0.05	0.50 (0.12-2.13)	-0.02 (-0.04-0.02)		
	Intermediate lifestyle	39/16080	0.20	2.03 (1.21-3.40)*	0.04 (0.02-0.08)	1.20 (0.23-2.17)	**0.008**
	Poor lifestyle	31/5638	0.46	4.64 (2.70-7.98)*	0.16 (0.09-0.21)	3.24 (1.20-5.29)	**0.001**

Abbreviations: CI, confidence interval; HLA, human leukocyte antigen; HR, hazard ratio; RERI, relative excess risk due to interaction;

[a]HLA allele–cancer pairs were selected using the same criteria as in the primary analyses after excluding participants with missing values for any covariates.

[b] HRs and 95% CIs were estimated from multivariable-adjusted Cox models with adjustment for baseline age, height, assessment center, Townsend deprivation index at recruitment, family history of cancer, and population structure (top 5 principal components), with HLA allele non-carriers with a favorable lifestyle as the reference group.

[c]Absolute risk differences in 5-year event rates and corresponding 95% CIs were estimated using bootstrap resampling (1,000 resamples).

[d]Additive interactions were quantified using the RERI, with 95% CIs estimated by the delta method. Statistically significant P values ($P < 0.05$) are shown in bold.

* Indicates statistical significance in the joint effect analyses ($P < 0.05$)
